# Computational study on palladium-catalyzed alkenylation of remote δ-C(sp^3^)–H bonds with alkynes: a new understanding of mechanistic insight and origins of site-selectivity†

**DOI:** 10.1039/c8ra06077k

**Published:** 2018-08-28

**Authors:** Hui-Min Yan, Ye Tian, Niu Li, Rong Chang, Zhu-Xia Zhang, Xiao-Yun Zhang, Wen-Jing Yang, Zhen Guo, Yan-Rong Li

**Affiliations:** College of Material Science & Engineering, Key Laboratory of Interface Science and Engineering in Advanced Materials, Ministry of Education, Taiyuan University of Technology Shanxi 030024 P. R. China guozhen@tyut.edu.cn; Department of Earth Science and Engineering, Taiyuan University of Technology Shanxi 030024 P. R. China li.dennis@hotmail.com

## Abstract

Palladium-catalyzed alkenylation of δ-C(sp^3^)–H bonds with alkynes was conducted by density functional theory calculations. The present study shows that the dimeric Pd_2_(OAc)_4_ mechanism reproduces experimental observations well, including regioselectivity and provides a deep mechanistic insight complementing the monomeric Pd(OAc)_2_ mechanism recently reported by Chen's group. In addition, the economical heterodimeric Ni–Pd(OAc)_4_ was predicted to be a potential species for such alkenylation of δ-C(sp^3^)–H bonds.

## Abstract 2

Palladium-catalyzed alkenylation of δ-C(sp^3^)–H bonds with alkynes was conducted by DFT calculations, showing that the dimeric Pd_2_(OAc)_4_ mechanism reproduces experimental observations well.

## Graphical abstract

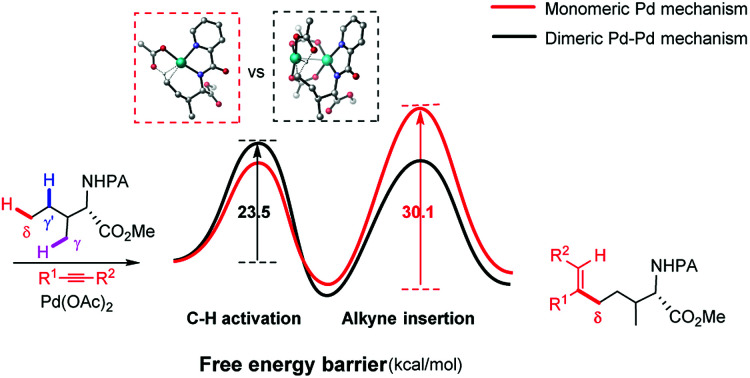

## 1. Introduction

**Scheme 1:**
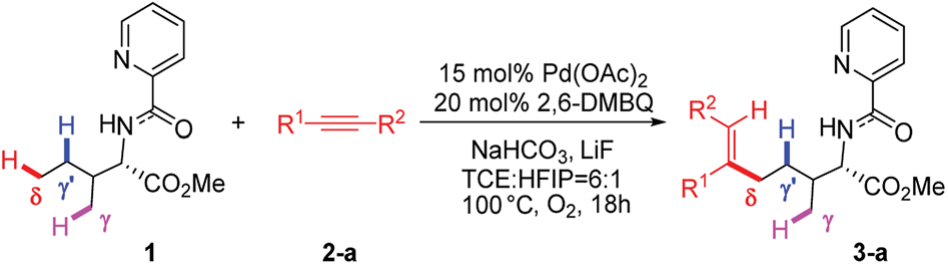
Pd-catalyzed site-selective alkenylation of δ-(sp^3^)–H with alkynes.

Transition metal-catalyzed C–H bond activations have received considerable attention in the past two decades due to their role in forming diverse atom economic C–C and C-heteroatom bonds in organic synthesis.^1^ Moreover, efficient and highly site-selective transformation of aliphatic C–H bonds is extremely useful for constructing biologically important compounds and building blocks in organic synthesis.^2^ In this regard, a variety of palladium-catalyzed functionalizations including arylation, alkylation, alkenylation, amination, borylation and carbonylation of aliphatic C–H bonds have been well achieved.^3^ Among these transformations, the C–H bond activations preferentially occur at the γ-methyl site *via* the kinetically more stable five-membered metallacycle. However, remote and inert δ-C(sp^3^)–H bond activation, especially in the presence of more reactive γ-C(sp^3^)–H bonds, is a significant challenge in this hot research field and remains in its infancy.^3*c*,3*f*^ One of the breakthroughs of direct δ-C(sp^3^)–H bond functionalization came from Shi's group, which exhibited the first palladium-catalyzed site-selective δ-C(sp^3^)–H bond alkenylation of aliphatic amines bearing more kinetically accessible γ-C(sp^3^)–H bonds (Scheme 1).^4^ The protocol is distinguished by its high functional-group tolerance and high site-selectivity with δ-C(sp^3^)–H bond activation. For this reaction, the crucial challenges are to understand the underlying factors controlling C–H bond activation and site-selectivity; more importantly, why the reaction regioselectively occurs at δ-methyl rather than kinetically more accessible γ-C–H sites. Intrigued by the unique site-selectivity in C–H bond activation, we have conducted computational studies to elucidate the titled reaction mechanism and origins of the site-selectivity.

It is worth mentioning that Chen *et al.* reported a computational study on the same reaction of Pd-catalyzed site-selective alkenylation of δ-C(sp^3^)–H bond with alkynes in 2017.^5^ The calculations indicated that the C–H activation step proceed *via* a six-membered palladacycle and the migratory insertion process was found to be both the rate-limiting and selectivity-determining step, where a monomer Pd(OAc)_2_ as the sole active species was involved in the whole reaction. The reaction mechanism involving dimeric Pd_2_(OAc)_4_ active species was suggested to be disfavored because the authors pointed out that N–H bond deprotonation within dimeric catalyst–reactant complex is prohibited by an extremely high barrier. However, for this reaction, we located a different transition-state configuration of N–H activation catalyzed by dimeric Pd_2_(OAc)_4_ and our calculated results show that N–H bond cleavage is relatively facile, associated with a lower reaction barrier that could be accessible in view of experimental conditions (1 atm, 100 °C). Therefore, mechanism involving dimeric Pd_2_(OAc)_4_ catalyst could not be simply eliminated and should be reconsidered. We herein report the results of our computational study to make deeper understanding to complement the newly-reported monomeric Pd(OAc)_2_ mechanism. To our delight, we could derive an unprecedented mechanism through dimeric Pd_2_(OAc)_4_ catalyst that the dimeric Pd_2_(OAc)_4_ mechanism may also explain the site-selectivity observed experimentally. In this dimeric Pd_2_(OAc)_4_ mechanism, the experimental site-selectivity originates exclusively from C–H activation process.

## 2. Computational details

All of the calculations were carried out with the Gaussian 09 program package.^6^ Geometry optimizations were performed at the B3LYP/BS1 level.^7^ BS1 designates a mixed basis set of LANL2DZ used for Pd and Ni,^8^ and the 6-31G (d,p) basis set was used for other atoms. Frequency analysis was conducted at the same level of theory to confirm the stationary points to be minima or saddle points. Intrinsic reaction coordination (IRC) calculations were used to verify the connections among the transition states and its reactant and product.^9^ Thermal correction to Gibbs free energy were obtained at the B3LYP/BS1 level. Single-point energies were calculated with M06 (ref. 10)/SDD^11^-6-311++G(d,p),^12^ which is generally considered to be more accurate for energetics. Single-point solvation energies were calculated by using SMD solvation model (solvent = tetrachloroethene (TCE)).^13^ All discussed energies in the follow refer to solvation free energy (Δ*G*_solv_, kcal mol^−1^) values, which was estimated as Δ*G*_solv_ = Δ*E*_solv_ (SMD) + Δ*G*(gas)_corre_, where Δ*E*_solv_ (SMD) refers to the calculated solvation single point energy and Δ*G*(gas)_corre_ refers to the calculated thermal correction in the gas phase for Gibbs free energies. Additional geometry optimizations were conducted in solvent at the B3LYP/BS1 level of theory employing the SMD solvation model, which suggested that these calculations give an identical conclusion to that of geometry optimization in gas phase. The computed free energy profile was given in ESI (Fig. S1†). Natural Bond Orbital (NBO) calculations were performed by using the GenNBO 5.0 program^14^ and the wave-function was obtained from the B3LYP/BS1 level. 3D structures for the optimized stationary points were prepared with CYL view.^15^

## 3. Results and discussion

### 3.1. Mechanism and site-selectivity in dimeric Pd_2_(OAc)_4_ catalyzed alkenylation of aliphatic amines

**Scheme 2:**
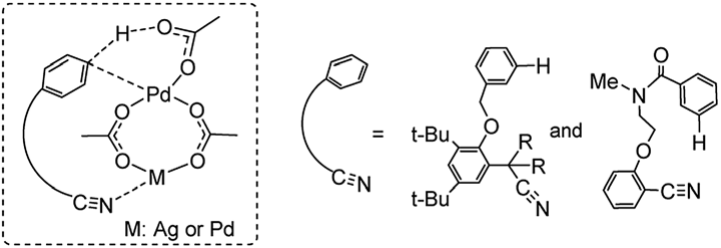
Dimeric metal catalysed C–H activation model.

Previous experimental and theoretical studies showed that acetate-bridged Pd–Pd(Ag) dimer complexes were crucial active species for remote C–H bond activations.^16^ In such a dimeric metal activation model (Scheme 2), one Pd(Ag) could bind to the directing group while another Pd is ideally positioned to the remote C–H bond. The unique coordination between catalyst and substrate may predistort the reactant into a transition-state-like conformation and benefit the formation of a larger-membered transition state with the relatively less ring strain. Inspired by this, the mechanism involving dimeric Pd_2_(OAc)_4_ catalyst was computationally investigated for the titled reaction.

**Fig. 1:**
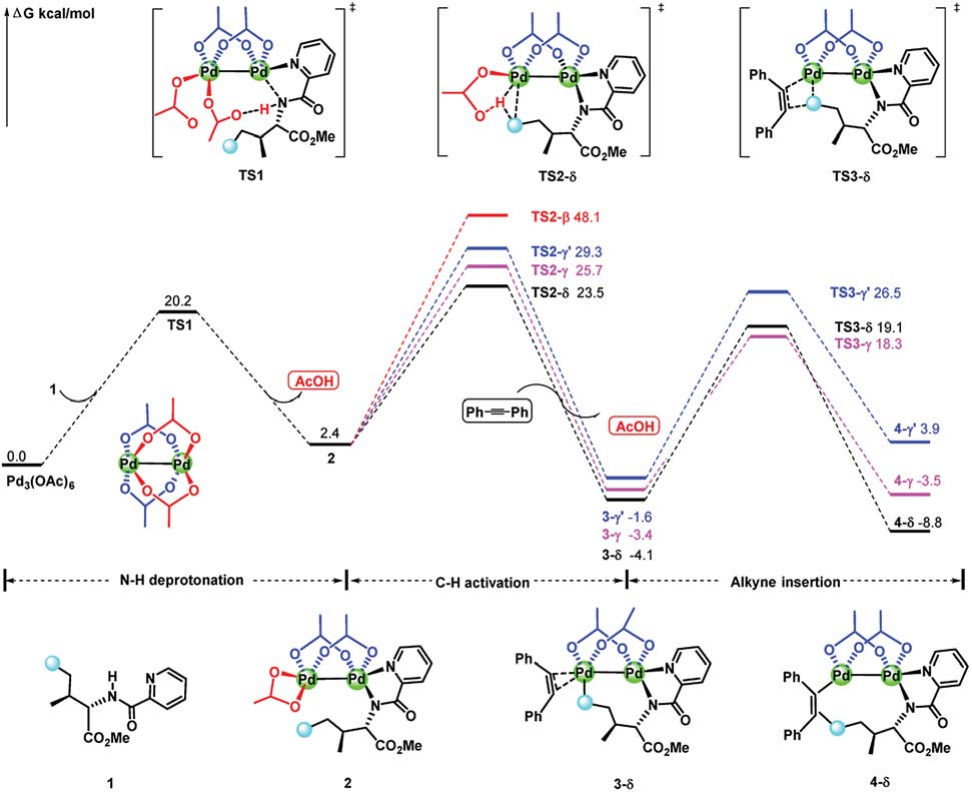
The simplified reaction potential energy surface of Pd_2_(OAc)_4_-catalyzed alkenylation of C(sp^3^)–H bond with alkynes. Energies are relative to Pd_3_(OAc)_6_ + 1 and are mass balanced.

For saving space, the calculated reaction potential energy surfaces (PESs) was simplified and depicted in Fig. 1 and the full PESs were included in ESI (Fig. S2–S6†). The reaction begins with N–H bond deprotonation assisted by the acetate ligand in Pd_2_(OAc)_4_ dimer to release the AcOH and generate intermediate 2. This N–H bond cleavage is thermodynamically and kinetically feasible with an accessible activation free energy of 20.2 kcal mol^−1^ at 100 °C, which is much lower in energy than that (50.3 kcal mol^−1^) reported by Chen and co-workers (see ref. 5). In parallel, the transformation of separated reactants to species 2 is slightly endergonic by 2.4 kcal mol^−1^. Subsequent C–H bond cleavage could be alternatively occur at several sites of dimeric Pd-amine intermediate 2 including β, γ, γ′ or δ-C(sp^3^)–H positions whereby the acetate ligand can act as an intramolecular base to promote C–H bond activation. The calculated results show that transition state TS2-δ for δ-C(sp^3^)–H bond activation lies lower in energy than those for β, γ, γ′-C(sp^3^)–H bond activations, as revealed by the order of the calculated energy barriers, 23.5 kcal mol^−1^ (TS2-δ) < 25.7 kcal mol^−1^ (TS2-γ) < 29.3 kcal mol^−1^ (TS2-γ′) < 48.1 kcal mol^−1^ (TS2-β). On the other hand, the process of δ-C(sp^3^)–H bond cleavage *via* distinct concerted metalation-deprotonation (CMD) mechanism, followed by the exchange coordination between AcOH and alkyne, is much more exergonic and generates the most stable Pd(ii)–Pd(ii)–π-alkyne species 3-δ. Thus, δ-C(sp^3^)–H bond activation is preferred in Pd(ii)–Pd(ii) complex 2, both kinetically and thermodynamically, likely owing to the less ring repulsion of transition state TS2-δ with a large-membered ring (eight-membered). Inspired by Schaefer's work,^17^ the alternative dimeric models (TS2-a, see Fig. S7†), in which PA (picolinamide) and the target C–H binding to the same Pd atom, was also considered. The active barrier of C–H activation for TS2-a is 37.0 kcal mol^−1^, being disfavored with respect to the TS2-δ. The next step corresponds to the migratory insertion of the alkyne into Pd–C bond from the different π-alkyne complexes (3-δ, 3-γ and 3-γ′) giving intermediates (4-δ, 4-γ and 4-γ′). As shown in Fig. 1, although γ-insertion *via* TS3-γ (Δ*G*^‡^ = 18.3 kcal mol^−1^) is slightly favorable than δ-insertion *via* TS3-δ (Δ*G*^‡^ = 19.1 kcal mol^−1^), the site-selective alkenylation of δ-C(sp^3^)–H would exclusively occur and give 4-δ due to the reasons: (i) δ-C(sp^3^)–H bond activation to yield the species 3-δ is kinetically and thermodynamically more accessible than its counterparts; (ii) starting from 3-δ, the reversible reaction of δ-C(sp^3^)–H bond activation requires much higher reaction barrier than the migratory insertion of alkyne. As such, once the species 3-δ is formed, the reaction could proceed ultimately to give the δ-alkenylation product. The final steps are two consecutive protonation processes to afford the desired products, which were calculated to be kinetically facile and not discussed in details (see ESI Fig. S6†).

### 3.2. Origins of site-selectivity and regioselectivity

**Fig. 2:**
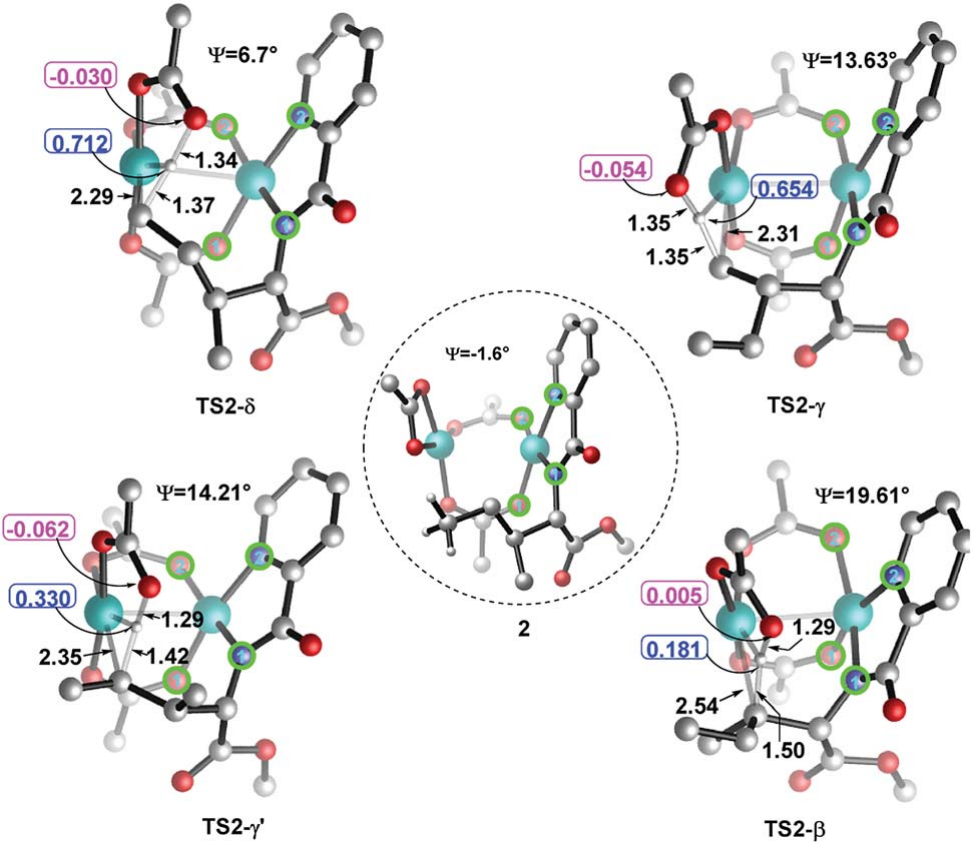
The dihedral angles (*Ψ*) of N1–N2–O2–O1 and NPA charges of selected atoms and bond lengths in transition states. The bond lengths are in angstrom.

Considering that the site-selectivity for alkenylation of δ-C(sp^3^)–H bond was determined by the C–H bond activation step, we compared the selected parameter (N1–N2–O2–O1 dihedral angles (*Ψ*) with green circle, Fig. 2) of the related transition states. As shown in Fig. 2, the dihedral angles of N1–N2–O2–O1 were arranged in the following order from biggest to smallest: 19.61° (TS2-β) > 14.21° (TS2-γ′) > 13.63° (TS2-γ) > 6.70° (TS2-δ). As such, with respect to the dihedral angles (−1.64°) of N1–N2–O2–O1 in species 2, the δ-structure has the smallest distortion among these transition states. This indicates that TS2-δ adopts a more similar conformation as in the reactant, which might translate into the lowest energy barrier (Δ*G*^‡^ = 23.5 kcal mol^−1^) in the δ-C(sp^3^)–H bond activation event. In addition, natural population analysis (NPA) shows that the NPA charge difference (Δ*q*, Fig. 2) between the leaving positive H atom and the negative O atom of acetate ligand in TS2-δ is much larger than its counterparts. For instance, Δ*q* is 0.74, 0.71, 0.39 and 0.19 in TS2-δ, TS2-γ, TS2-γ′ and TS2-β, respectively. Therefore, the δ-site H atom is more facile to be transferred by the electrostatic interaction with the acetate ligand.

Shi's experimental results show that the regioselectivity of the palladium-catalyzed alkenylation of δ-C(sp^3^)–H bond was sensitive to changes in the substituents of internal alkyne. As seen in Table 1, asymmetric alkyne MeC≡CPh exhibited excellent regioselectivity, whereas asymmetric alkyne MeC≡C^i^Pr achieved much poor selectivity. To shed light on experimentally observed regioselectivity, we calculated the two competing insertion steps with substrates of MeC≡CPh or MeC≡C^i^Pr. The results reveal that the energy barrier difference between two transition states connecting to two isomers of products (A and B) in substrate of MeC≡CPh (ΔΔ*G*^‡^ = 1.74 kcal mol^−1^) is much larger than that in substrate of MeC≡C^i^Pr (ΔΔ*G*^‡^ = 0.26 kcal mol^−1^), indicating a high regioselectivity in case of the former (see Table 1 and Fig. S8†). This well reproduces the regioselectivity observed experimentally (10 : 1 for MeC≡CPh *vs.* 1.8 : 1 for MeC≡C^i^Pr). The Mulliken charge analysis (Fig. S9†) indicates that as the reaction goes from 3-δ to transition state TS3-δ and then to 4-δ, the total NPA charges of alkyne change to be more negative, revealing that the alkyne participates in the insertion step as an electrophile with its unoccupied π* orbital accepting the electrons from the occupied σ-orbital of dimeric Pd-amine species. Therefore, the alkenylation reaction will be mainly controlled by the orbital interaction between the LUMO of alkynes and σ-orbital of dimeric Pd-amine. As such, asymmetric alkynes prefer to use the alkyne carbon with the larger LUMO coefficient to form the C–C bond for the stronger orbital interaction. To verify this hypothesis, we calculated the LUMO coefficients for alkynes (MeC≡CPh and MeC≡C^i^Pr) and compared with experimental regioselectivity (Table 1). Indeed, methyl substituted carbon in MeC≡CPh has a much larger LUMO coefficient than phenyl substituted carbon (15.98 *vs.* 4.87 for C^α^*vs.* C^β^), while this difference is lost and a comparable LUMO distribution occurs in MeC≡C^i^Pr (28.51 *vs.* 26.76 for C^α^*vs.* C^β^). That is why the high regioselectivity was observed in substrate MeC≡CPh rather than MeC≡C^i^Pr. Thus, the Frontier molecular orbital rationale could be well applicable in support of the experimentally observed regioselectivity as found in previous theoretical studies.^18^

**Table 1:** Pd_2_(OAc)_4_-catalyzed asymmetric alkynes insertion: regioselectivity, free energy barrier difference and LUMO π* orbital coefficients of the alkynes

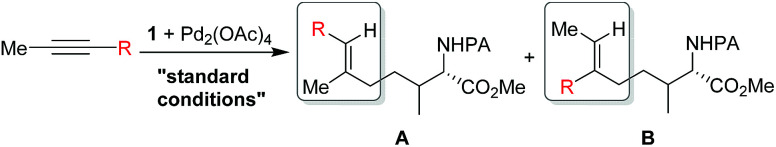		
Compound	R = Ph	R = ^i^Pr
Regioselectivity (A : B)	10 : 1	1.8 : 1
ΔΔ*G*^‡^ (kcal mol^−1^)	1.74	0.26
Orbital coefficient (%)	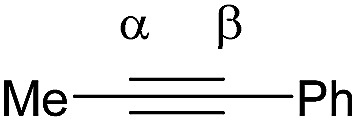	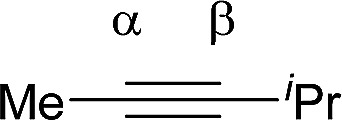
LUMO	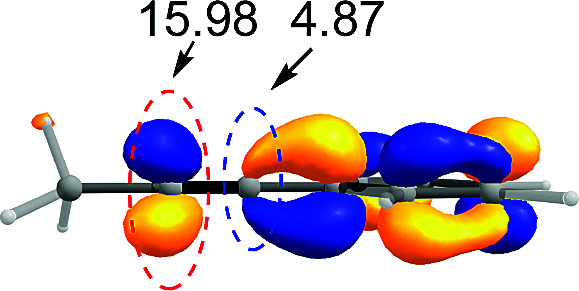	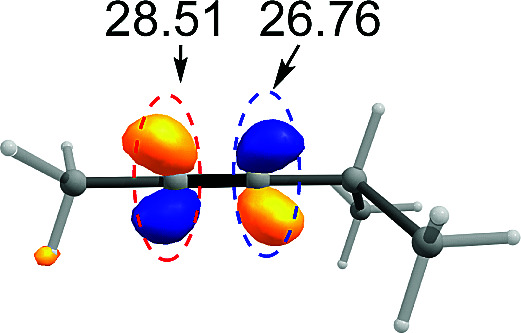

### 3.3. Summary of dimeric Pd_2_(OAc)_4_ mechanism and monomeric Pd(OAc)_2_ mechanism

In the previous mechanistic study on palladium-catalyzed alkenylation of δ-C(sp^3^)–H bond with alkynes, it was proposed that this reaction proceeds *via* Pd(OAc)_2_ monomer.^5^ In order to eliminate the influence of computational method on conclusion, alkenylation of δ-C(sp^3^)–H bond with alkynes mediated by monomeric Pd(OAc)_2_ was re-calculated at the level of theory used in this study. The conclusions drawn from our calculated results were consistent with that reported by Chen's group (see Fig. S10†). In the monomeric Pd(OAc)_2_ mechanism, γ and γ′-C(sp^3^)–H bond activations *via* five-membered palladacycles are kinetically favoured kcal mol^−1^ compared to the δ-C(sp^3^)–H bond activation where migratory insertion is both the rate-limiting and the selectivity-determining step. By contrast, our dimeric Pd_2_(OAc)_4_ mechanism revealed that δ-C(sp^3^)–H bond activation requires the smaller activation free energy than other sites and reversible δ-C(sp^3^)–H bond activation is unlikely due to the smaller reaction barrier needed in the subsequent alkyne insertion (23.5 *vs.* 19.1 kcal mol^−1^ for TS2-δ *vs.* TS3-δ, Fig. 1), thus, in dimeric Pd_2_(OAc)_4_ mechanism, C(sp^3^)–H bond activation and migratory insertion are exclusively arisen from the δ-site of substrates.

Previous literature has demonstrated that homogeneous Pd(OAc)_2_ can exist in various aggregated forms in solvent including monomer Pd(OAc)_2_, dimer Pd_2_(OAc)_4_, or trimer Pd_3_(OAc)_6_ species.^19^ Thus, we conceived that both monomeric and dimeric Pd active species could be co-exist in the titled reaction mixture. However, the equilibrium between monomeric Pd(OAc)_2_ and dimeric Pd_2_(OAc)_4_ is highly dependent on Pd-catalyst concentration experimentally used. At this stage, we cannot conclude whether monomeric Pd(OAc)_2_ or both monomeric Pd(OAc)_2_ and dimeric Pd_2_(OAc)_4_ are responsible for such alkenylation reaction of δ-C(sp^3^)–H bond.

### 3.4. Can heterodimeric Ni–Pd(OAc)_4_ catalyst be used in the alkenylation of the δ-C(sp^3^)–H bond with alkynes?

Yu *et al.* proposed and Houk *et al.* computationally showed that some C–H bond functionalizations prefer to take place *via* heterodimeric metal species compared to homodimeric metal species such as PdAg-(OAc)_3_*versus* Pd_2_(OAc)_4_.^16^ Illuminated by these closely relevant precedents and the fact that Ni–Pd(OAc)_4_ species has similar dimeric structure with Pd_2_(OAc)_4_,^20^ we envisioned that heterodimeric Ni–Pd(OAc)_4_ could be utilized to complete the catalytic cycle in a manner like dimeric Pd_2_(OAc)_4_ catalyst. The computational results are given in Fig. S11,† which show that heterodimeric Ni–Pd(OAc)_4_ could promote alkenylation reaction of δ-C(sp^3^)–H bond with alkynes and behave similarly to Pd_2_(OAc)_4_ dimer. We expect that such a good alternative for more expensive palladium catalyst can be used in development of new relevant C–H bond functionalizations.

## 4. Conclusions

In conclusion, we have investigated the mechanism and origins of site-selectivity for alkenylation of δ-C(sp^3^)–H bond with alkynes catalyzed by dimeric Pd_2_(OAc)_4_, which could be complementary to or collaborate with the monomeric Pd pathway to understand the titled reactions. In dimeric Pd_2_(OAc)_4_ mechanism, the δ-C(sp^3^)–H bond activation step requires much lower activation free energy than other sites due to less ring strain in a larger membered-ring transition state and exclusively responsible for the origins of site-selectivity. This is in contrast to monomeric mechanism where alkyne insertion is the rate-limiting and selectivity-determining step. We also extended the mechanistic computations to probe new possibilities involving the cheaper heterodimeric Ni–Pd(OAc)_4_, demonstrating that this species has the great potential in developing new remote C–H bond functionalizations.

## Conflicts of interest

There are no conflicts to declare.

## Supplementary Material

RA-008-C8RA06077K-s001
