# Supplementary material for: Computational study on palladium-catalyzed alkenylation of remote δ-C(sp3)–H bonds with alkynes: a new understanding of mechanistic insight and origins of site-selectivity
Source: RSC Adv. 2018 Aug 28;8(53):30186–90. doi: 10.1039/c8ra06077k (PMC9085394; doi:10.1039/c8ra06077k)
Supplement: RA-008-C8RA06077K-s001 [file RA-008-C8RA06077K-s001.pdf]

Electronic Supplementary Material (ESI) for RSC Advances.
This journal is © The Royal Society of Chemistry 2018

Supporting Information

Computational Study on Palladium-Catalyzed Alkenylation of Remote δ-C(sp^3)-H Bond with Alkynes: A New Understanding of Mechanistic Insight and Origins of Site-Selectivity

Hui-Min Yan,[a] Niu Li,[a] Ye, Tian,[a] Rong Chang,[a] Zhu-Xia Zhang,[a] Xiao-Yun Zhang,[a] Wen-Jing Yang,[a] Zhen Guo*[a] and Yan-Rong Li*[b]

[a]*College of Material Science & Engineering, Key Laboratory of Interface Science and Engineering in Advanced Materials, Ministry of Education, Taiyuan University of Technology, Shanxi 030024 P. R. China.*

[b]*Department of Earth Science and Engineering, Taiyuan University of Technology, Shanxi 030024, P. R. China.*

Email: guozhen@tyut.edu.cn; li.dennis@hotmail.com

Table of Contents

1. Computed simplifiled reaction energy profile of $Pd_2(OAc)_4$-catalyzed alkenylation of δ-C(sp^3)-H bond with alkynes with the geometry optimization being performed at the B3LYP/BS1 level in solvents employing the SMD solvation model..........3

2. Complete free energy profiles for the dimeric $Pd_2(OAc)_4$ catalyzed C(sp^3)-H bond alkenylation of aliphatic amines..........4

3. Alternative dimeric $Pd_2(OAc)_4$ catalyzed δ-C(sp^3)-H bond activation model 6

4. Origin of regioselectivity...........7

5. Monomer $Pd(OAc)_2$ catalyzed C(sp^3)-H bond alkenylation of aliphatic amines..........8

6. Heterodimer Ni-Pd$(OAc)_4$ catalyzed δ-C(sp^3)-H bond activation and PhC≡CPh migratory insertion into the C-Pd bond..........9

7. Cartesian coordinates of all structures..........10

8. Cartesian coordinates of structures in Figure S1..........61

1. Computed reaction energy profile of $Pd_2(OAc)_4$-catalyzed alkenylation of δ-C(sp^3)-H bond with alkynes with the geometry optimization being performed at the B3LYP/BS1 level in solvents employing the SMD solvation model

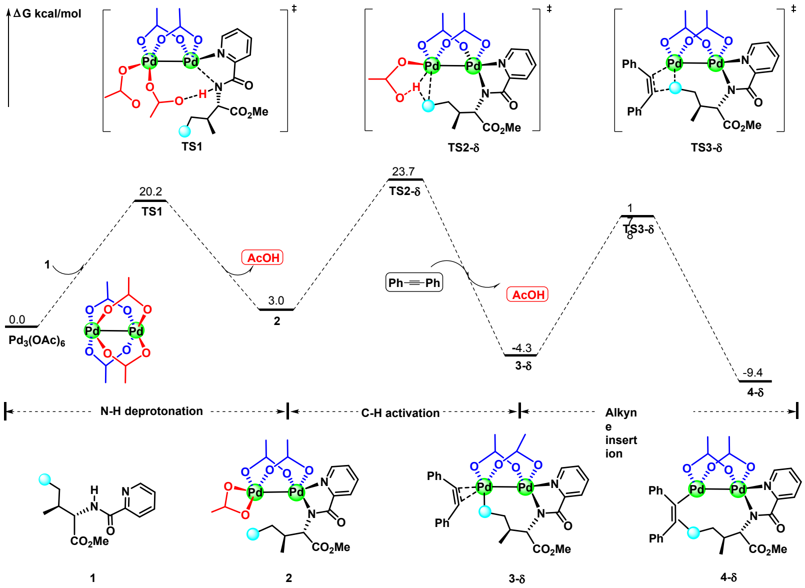

Figure S1 Computed reaction energy profile of $Pd_2(OAc)_4$-catalyzed alkenylation of δ-C(sp^3)-H bond with alkynes were performed at SMD(TCE)-M06/SDD/6–311++G(d,p)//SMD(TCE)-B3LYP/LANL2DZ/6-31G(d,p).

2. Complete free energy profiles for the dimeric $Pd_2(OAc)_4$ catalyzed C(sp^3)-H bond alkenylation of aliphatic amines

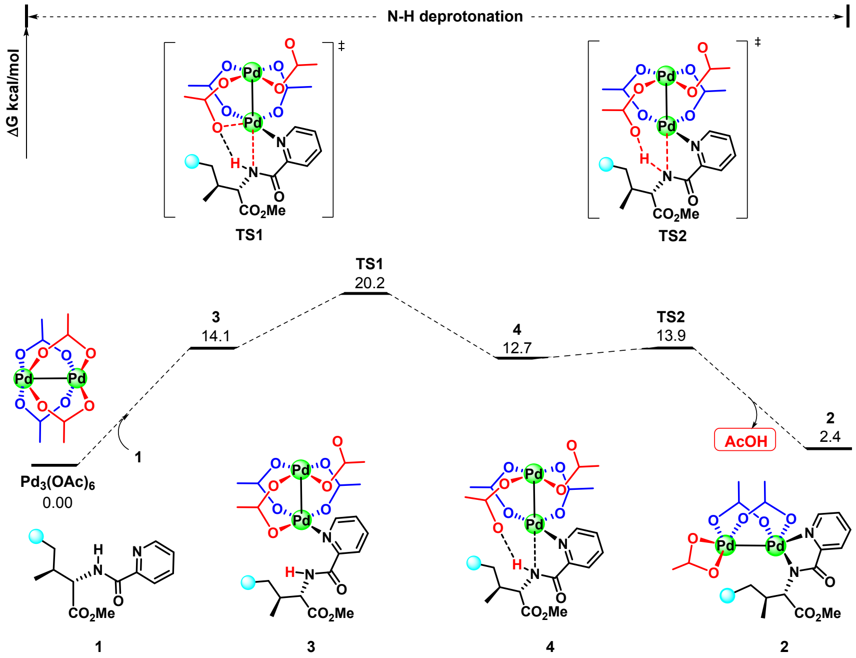

Fig. S2 Reaction energy profile of the N-H bond activation catalyzed by dimeric $Pd_2(OAc)_4$. All energies are relative to the $Pd_3(OAc)_6$ + 1 and are mass balanced (similarly hereinafter).

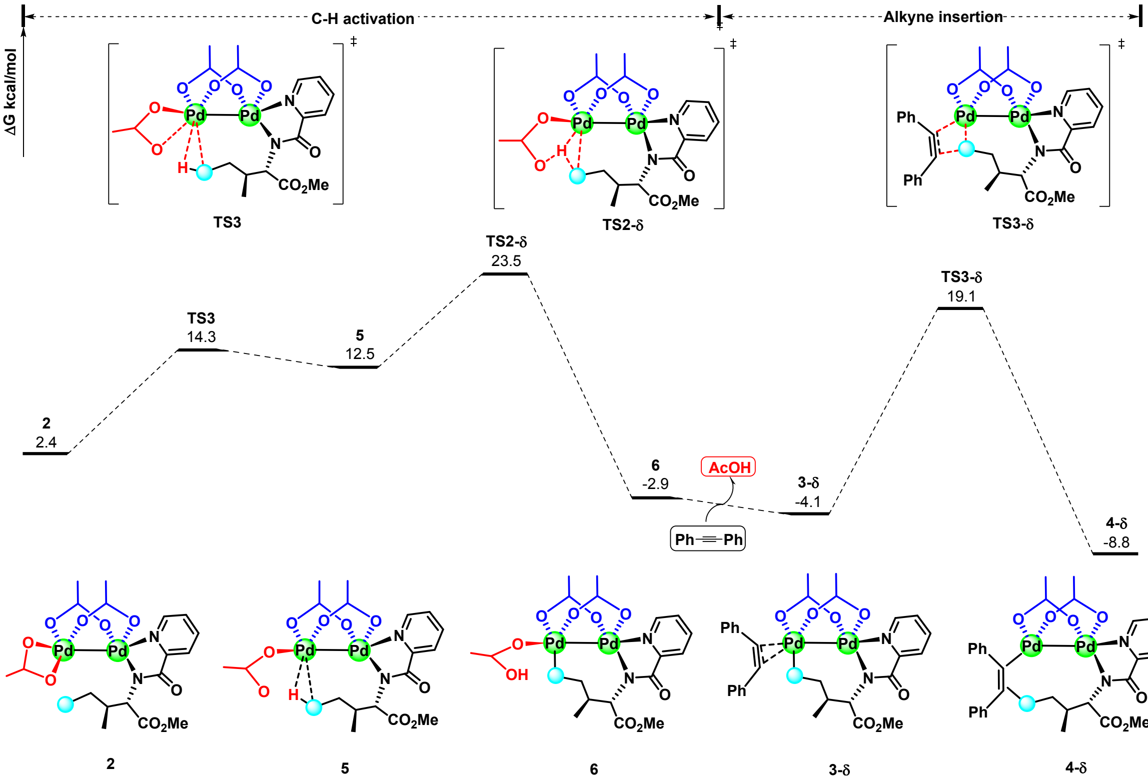

Fig. S3 Reaction energy profile of the dimeric $Pd_2(OAc)_4$ catalyzed δ-C(sp^3)-H bond activation and PhC≡CPh migratory insertion into the C-Pd bond.

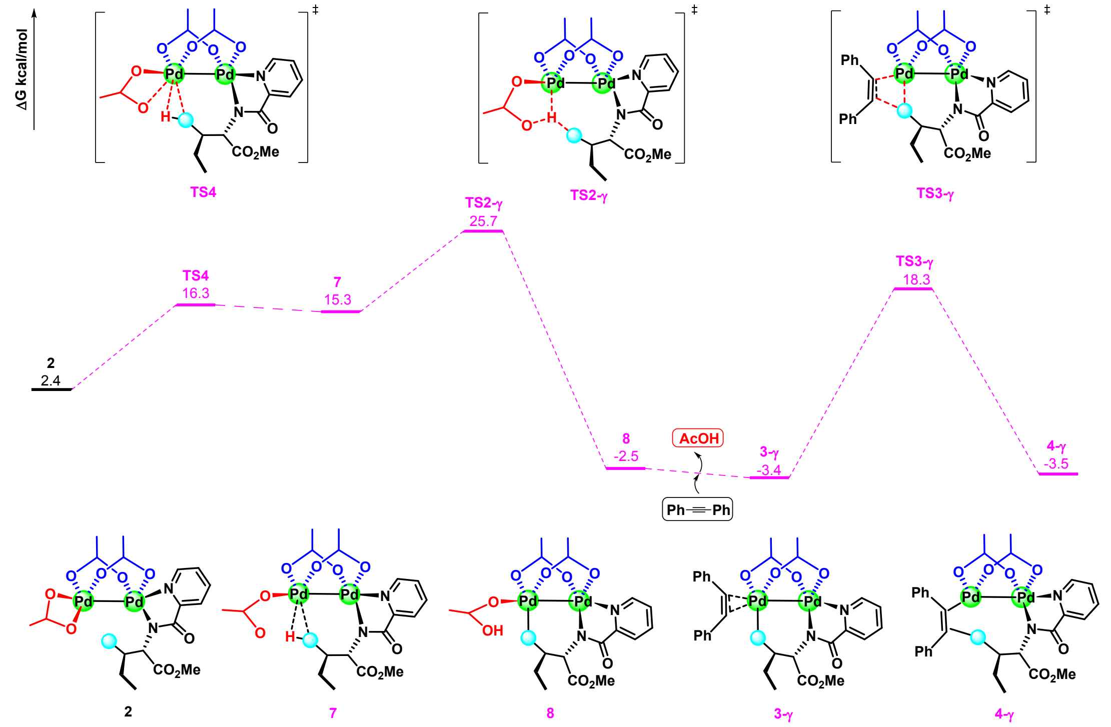

Fig. S4 Reaction energy profile of the dimeric $Pd_2(OAc)_4$ catalyzed γ-C(sp^3)-H bond activation and PhC≡CPh migratory insertion into the C-Pd bond.

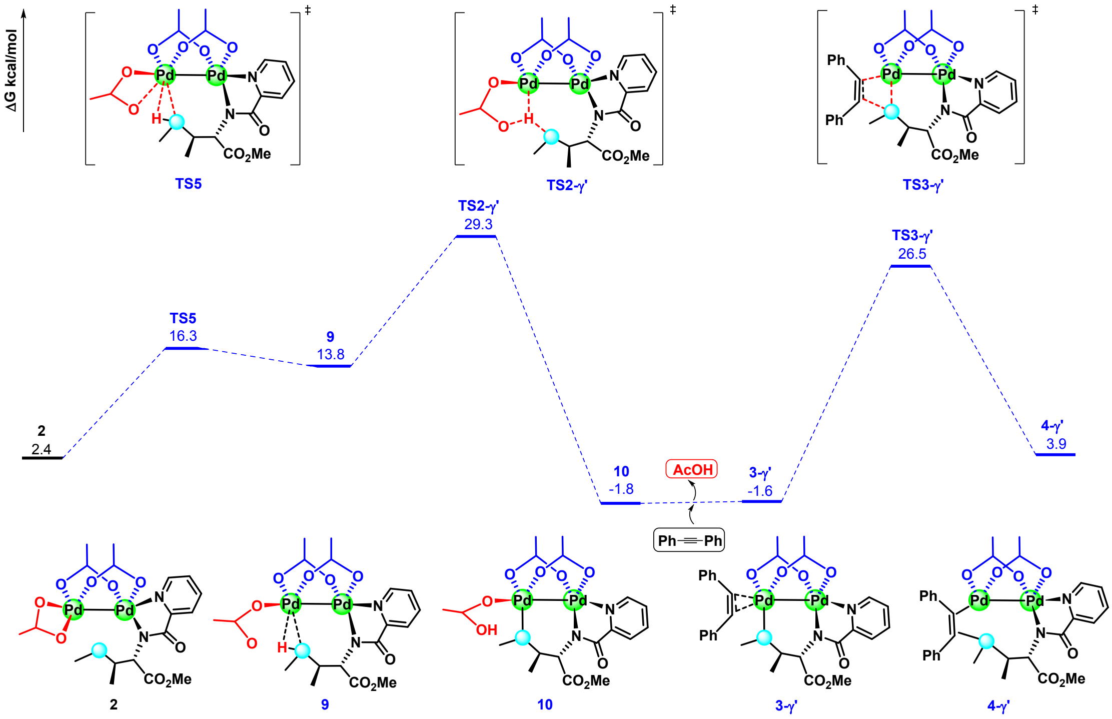

Fig. S5 Reaction energy profile of the dimeric $Pd_2(OAc)_4$ catalyzed γ'-C(sp^3)-H bond activation and PhC≡CPh migratory insertion into the C-Pd bond.

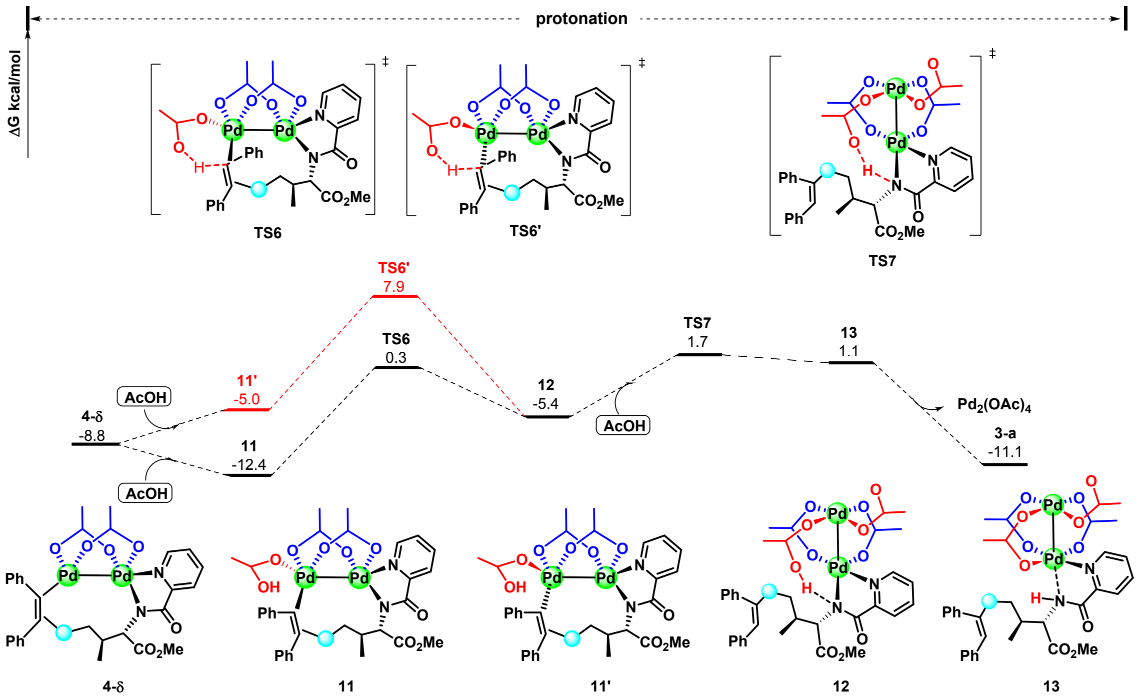

Fig. S6 Reaction energy profile of two protonation steps.

3. Alternative dimeric $Pd_2(OAc)_4$ catalyzed δ-C(sp^3)-H bond activation model

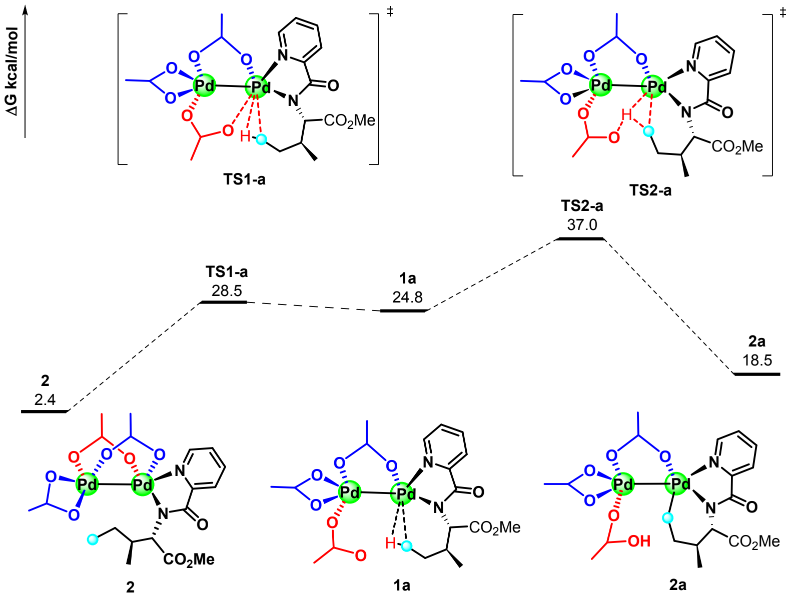

Fig. S7 Free energy profiles for alternative dimeric $Pd_2(OAc)_4$ catalyzed δ-C(sp^3)-H bond activation.

4. Origin of regioselectivity.

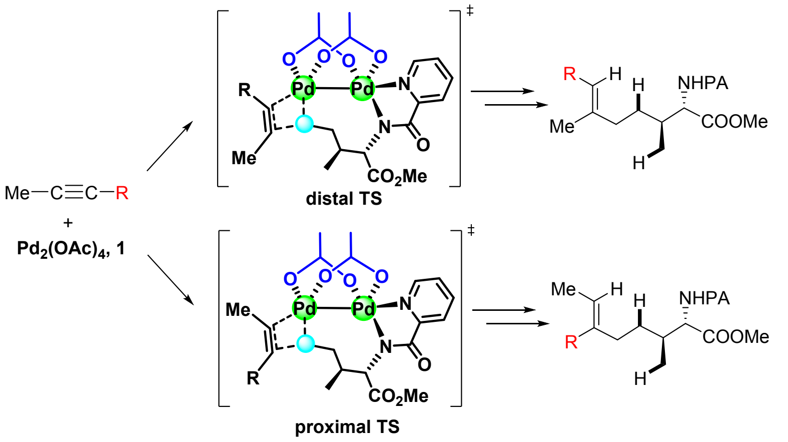

Free energy barrier	Me—C≡C—Ph	Me—C≡C—^{i}Pr
distal TS (kcal/mol)	22.9	24.0
proximal TS (kcal/mol)	24.6	24.3

Fig. S8 Relative free energies of $Pd_2(OAc)_4$ catalyzed asymmetric alkynes insertion.

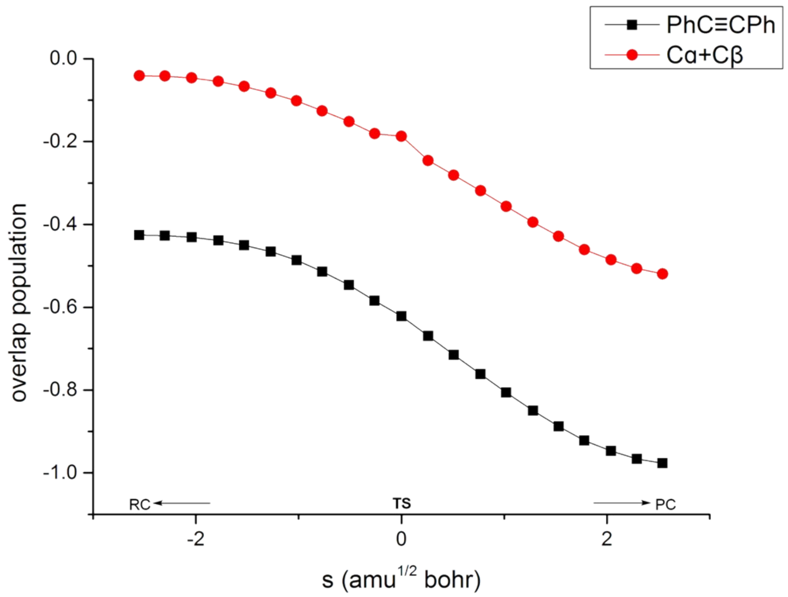

Fig. S9 Change in Mulliken charge along the IRC of PhC≡CPh

5. Monomer $Pd(OAc)_2$ catalyzed $C(sp^3)$-H bond alkenylation of aliphatic amines

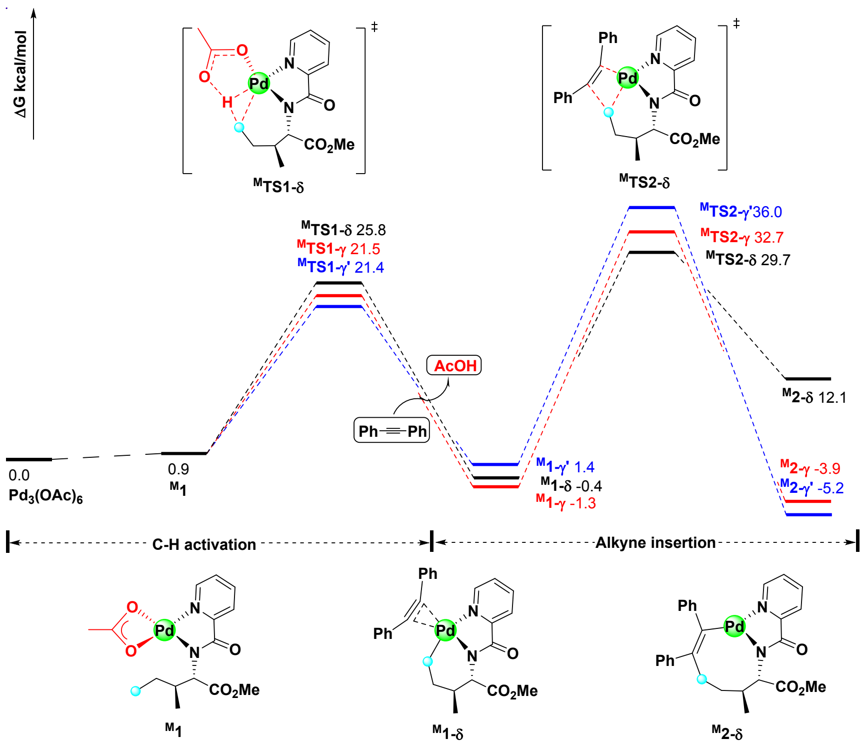

Fig. S10 Reaction energy profile of monomer $Pd(OAc)_2$ catalyzed δ-$C(sp^3)$-H bond alkenylation of aliphatic amines.

6. Heterodimer Ni-Pd(OAc)$_4$ catalyzed δ-C(sp^3)-H bond activation and PhC≡CPh migratory insertion into the C-Pd bond

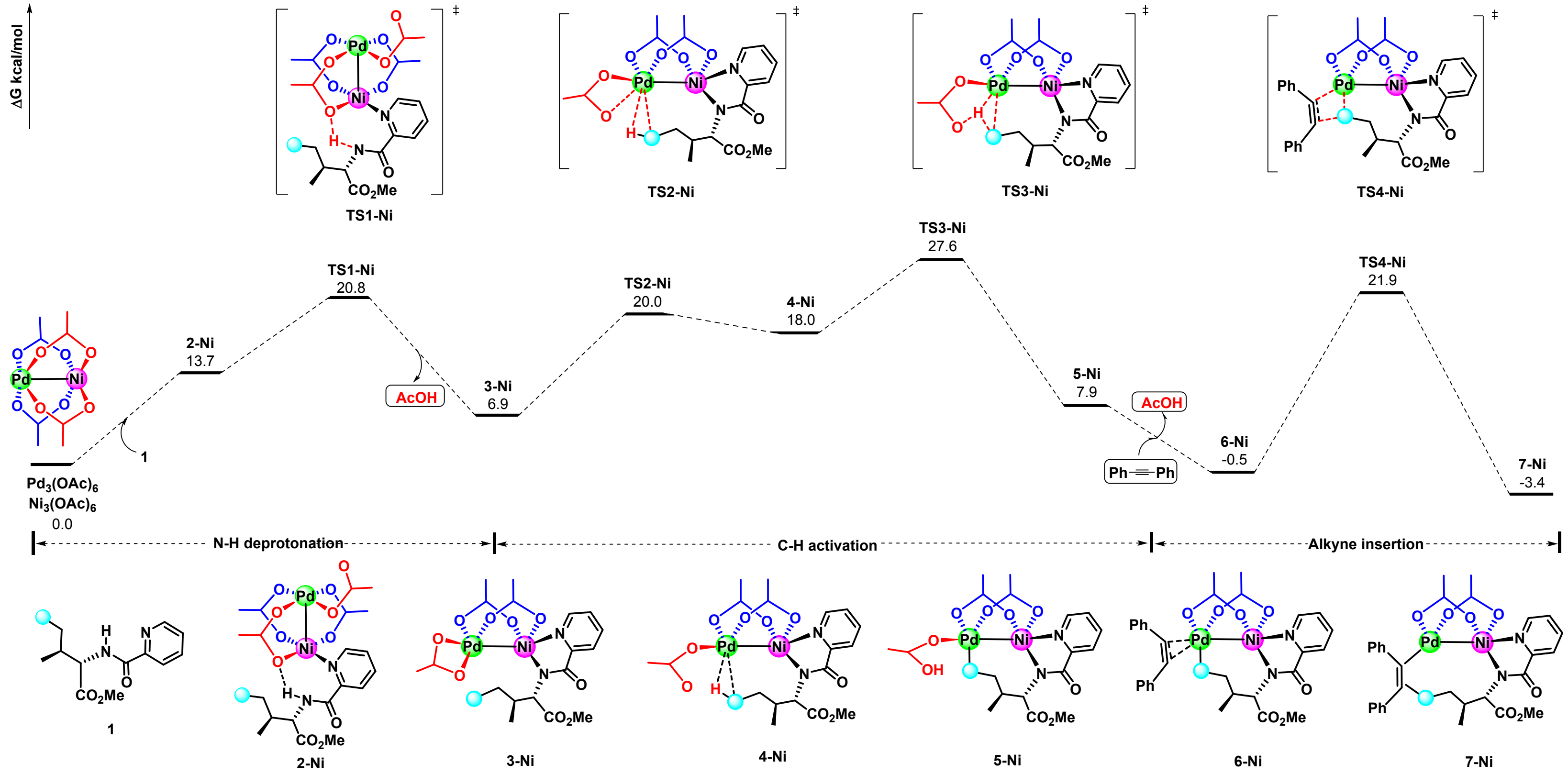

Fig. S11 Reaction energy profile of the heterodimer Ni-Pd(OAc)$_4$ catalyzed δ-C(sp^3)-H bond activation and PhC≡CPh migratory insertion into the C-Pd bond. All energies are relative to $Pd_3(OAc)_6$ + $Ni_3(OAc)_6$ + 1 and are mass balanced.

7. Cartesian coordinates of all structures

$[Pd(OAc)_2]_3$

Thermal correction to Gibbs Free Energy= 0.241592 Hartree

SCF Done: E(RM06) = -1754.45109902 Hartree

0 1

Pd	1.61303700	0.92936600	0.00099800
C	2.24684300	-1.35660600	1.87412900
O	2.63339200	-0.30430700	1.28580300
O	1.20187400	-2.03559400	1.64066900
C	-2.24800000	-1.35410900	-1.87420300
O	-2.63344000	-0.30134800	-1.28597100
O	-1.20386900	-2.03432100	-1.64059000
C	-3.15782700	-1.88273100	-2.96523000
H	-2.58512400	-2.47359900	-3.68106700
H	-3.91015000	-2.53214500	-2.50491900
H	-3.67135200	-1.05825300	-3.46147300
C	3.15591000	-1.88602400	2.96539800
H	3.66984200	-1.06198000	3.46194800
H	2.58256800	-2.47661500	3.68095100
H	3.90796000	-2.53584600	2.50522400
Pd	-1.61233800	0.93126900	-0.00086400
C	-2.29897000	-1.26736300	1.87268200
O	-1.57973300	-2.12725200	1.28437500
O	-2.36628400	-0.02297100	1.63927800
C	2.29760300	-1.26943100	-1.87293300
O	2.36602100	-0.02512000	-1.63947500
O	1.57783900	-2.12870500	-1.28439500
C	3.20923900	-1.79375200	-2.96452800
H	2.75510700	-2.65571700	-3.45485900
H	3.42749700	-1.00462400	-3.68512800
H	4.15153500	-2.11236900	-2.50609000
C	-3.21170900	-1.79076100	2.96379700
H	-2.76161400	-2.65690100	3.45041800
H	-3.42447800	-1.00293100	3.68749500
H	-4.15662000	-2.10215200	2.50577300
Pd	-0.00081600	-1.85995800	0.00003700
C	-0.04685200	2.62320400	-1.87347300
O	1.05667100	2.43056600	-1.28348900
O	-1.15846700	2.05934000	-1.64158500
C	-0.04759200	3.67506000	-2.96489700
H	0.92272200	3.70402700	-3.46231300
H	-0.84717800	3.47702100	-3.67969900
H	-0.22963900	4.65213300	-2.50468600
C	0.04938400	2.62318400	1.87354200
O	1.16020700	2.05760800	1.64185900
O	-1.05447100	2.43171500	1.28385500
C	0.05243300	3.67556100	2.96445700
H	0.25661100	4.64925200	2.50636000
H	-0.92314600	3.71995800	3.45011200
H	0.84018800	3.46533200	3.68897400

$[Ni(OAc)_2]_3$

Thermal correction to Gibbs Free Energy= 0.248125 Hartree

SCF Done: E(RM06) = -1883.74787442 Hartree

0 1

C	2.46292400	-0.48380700	-1.74530200
O	2.04647800	-1.51444800	-1.14041600
O	2.11215400	0.71590700	-1.52962300
C	-0.72617100	2.40149400	1.74518000
O	-1.71049000	1.88713100	1.13853100
O	0.50224800	2.16805900	1.53213300
C	-1.04313600	3.42315100	2.81615800
H	-0.27375500	3.40725900	3.58959200
H	-1.04231400	4.41815100	2.35769600
H	-2.02910800	3.23666500	3.24293900
C	3.50989300	-0.69840800	-2.81724100
H	3.41888900	-1.69670800	-3.24639900
H	3.41971700	0.06774300	-3.58878400
H	4.50042400	-0.60306500	-2.35902200
C	-0.81095800	2.37554700	-1.74329400
O	0.28889800	2.52996100	-1.13659600
O	-1.67452100	1.47128500	-1.53003200
C	2.44524200	-0.57407100	1.74353100
O	1.63063300	-1.52226400	1.52840300
O	2.49057200	0.53672000	1.13875900
C	3.48837300	-0.81060400	2.81459000
H	3.81833400	0.13636400	3.24288300
H	3.09037400	-1.47053600	3.58692500
H	4.35055900	-1.30704800	2.35592500
C	-1.14738200	3.39055800	-2.81467200
H	-0.23684700	3.81146800	-3.24225500
H	-1.76468800	2.92998600	-3.58746700
H	-1.72599300	4.20015500	-2.35645700
C	-1.71798200	-1.82961200	1.74428200
O	-0.77865900	-2.42306100	1.13843400
O	-2.13340000	-0.65049000	1.52989600

C	-2.44219700	-2.61581500	2.81604700
H	-1.78434200	-3.37160800	3.24627600
H	-2.81834700	-1.94144300	3.58683000
H	-3.30050500	-3.11887100	2.35730700
C	-1.65293300	-1.88946400	-1.74428900
O	-0.43762800	-2.18332900	-1.53077000
O	-2.33766600	-1.01425200	-1.13873200
C	-2.36270200	-2.69066300	-2.81459300
H	-2.77045400	-3.59805600	-2.35569300
H	-3.18517300	-2.11550700	-3.24094800
H	-1.65531500	-2.99255800	-3.58847200
Ni	-1.78952600	0.38572400	-0.00012200
Ni	0.56039900	-1.74041700	-0.00098100
Ni	1.22847600	1.35497900	0.00099000

1

Thermal correction to Gibbs Free Energy= 0.252206 Hartree

SCF Done: E(RM06) = -841.116994337 Hartree

0 1

C	2.59876500	-0.20032900	-0.23659900
C	3.66362600	-0.95354700	-0.73630700
C	4.95728200	-0.59482400	-0.36454400
C	5.13377400	0.49599700	0.48708100
C	4.00285100	1.18476000	0.93225800
H	3.44978800	-1.78790800	-1.39404900
H	5.81274300	-1.15469900	-0.73134600
H	6.12340700	0.81074500	0.80266100
H	4.10277100	2.04055000	1.59703000
N	2.75709100	0.85044800	0.58293300
C	1.19014200	-0.57673900	-0.63308700
O	0.96440900	-1.52480500	-1.38647200
N	0.22787200	0.20825900	-0.09168600
H	0.50943800	0.99702100	0.48030100
C	-1.17559400	0.02716900	-0.40828100
C	-1.81534600	-1.13985000	0.41683900
H	-1.26113300	-0.23699900	-1.46780100
C	-3.18412700	-1.56949300	-0.14558000
C	-3.68501900	-2.90769500	0.41139400
H	-3.92866100	-0.78829900	0.04766200
H	-3.10575600	-1.64910900	-1.23746800
H	-4.62555200	-3.19927000	-0.06733500
H	-2.95865200	-3.70823200	0.23071800
H	-3.86852900	-2.86183100	1.48959800
C	-1.86134100	1.36768400	-0.17473300
O	-1.42092500	2.24945800	0.53438300
O	-3.02888900	1.45627900	-0.83774100
C	-3.75976200	2.68096900	-0.64018300
H	-4.66484900	2.58105500	-1.23842500
H	-3.16831400	3.53604400	-0.97563800
H	-4.00782600	2.81470700	0.41541600
H	-1.11960100	-1.97221500	0.25279900
C	-1.85191100	-0.82257300	1.91787000
H	-2.12993300	-1.70634000	2.49864300
H	-2.57817200	-0.03337900	2.14636700
H	-0.87270800	-0.48942600	2.27401400

3

Thermal correction to Gibbs Free Energy= 0.426642 Hartree

SCF Done: E(RM06) = -2010.74196813 Hartree

0 1

Pd	2.58272400	-0.93871600	-0.49321000
O	1.07203100	0.10759900	2.30337200
C	2.27989000	-0.29981500	2.44329200
O	3.06551200	-0.75985000	1.56741200
C	2.81048500	-0.23960600	3.86460100
H	3.90002000	-0.19735000	3.85845200
H	2.38974100	0.61778100	4.39267500
H	2.49926900	-1.14822800	4.39102900
Pd	0.12505900	-0.01390100	0.46896500
C	4.70966500	0.63825300	-1.31348000
O	3.48162800	0.81568700	-0.85702400
O	5.21763400	-0.45746100	-1.50934200
C	5.43810400	1.94888300	-1.57062500
H	6.41660300	1.74314400	-2.00567500
H	4.85566000	2.58099300	-2.24761800
H	5.56416200	2.49628100	-0.63088900
C	0.32999300	-0.49631700	-2.46313300
O	-0.49019400	-0.15762300	-1.52741400
O	1.53572400	-0.83324500	-2.34982900
C	0.63473100	-2.95295500	0.38318300
O	1.76357100	-2.79217400	-0.15682000
O	-0.16355100	-2.02974700	0.74857100
C	0.18362000	-4.37310800	0.64892400
H	0.29896200	-4.58574500	1.71683900
H	-0.87311100	-4.48374000	0.39657500
H	0.79120900	-5.07633800	0.07950500
C	-0.26228200	-0.50381000	-3.85997600
H	-0.83050900	0.41340200	-4.03272900

H	0.52723400	-0.60951500	-4.60349900
H	-0.95660100	-1.34568500	-3.94735100
C	-1.02661900	2.80027000	0.45611500
C	-0.97349400	4.19583100	0.48344400
C	0.24447000	4.84532200	0.31839900
C	1.39203100	4.07548200	0.14635400
C	1.28705900	2.68863100	0.15847700
H	-1.89916200	4.73356100	0.64772100
H	0.29583300	5.92974900	0.32712800
H	2.36793700	4.52782900	0.00959500
H	2.16092400	2.05938500	0.02157900
N	0.10003900	2.06392000	0.30970100
C	-2.39953500	2.18526600	0.66992800
O	-3.15262400	2.72724000	1.47396400
C	-4.00229400	0.41557200	0.12361700
C	-4.73844200	0.12786800	-1.20912100
H	-4.60909700	1.11124900	0.71575900
C	-6.23677000	-0.15909900	-0.96790300
H	-4.67564400	1.07628500	-1.76227500
C	-7.08045100	-0.17191100	-2.24772400
H	-6.33769700	-1.11347000	-0.44246900
H	-6.63407800	0.61236200	-0.29421100
H	-6.79626800	-0.98819100	-2.91906400
C	-3.81031400	-0.81784400	1.02753100
O	-4.42164100	-1.86112900	0.92630600
O	-2.91657300	-0.56814400	1.99877400
C	-4.05434900	-0.96191000	-2.05287000
H	-4.17589500	-1.94311300	-1.58718000
H	-2.98208500	-0.77458700	-2.17054000
H	-4.48795400	-1.00160900	-3.05610800
C	-2.68821900	-1.63605300	2.93781000
H	-2.06833500	-1.20251200	3.72160100
H	-2.15688900	-2.45105900	2.44397200
H	-3.63631800	-1.99722700	3.34171900
H	-8.14085300	-0.30206200	-2.00900000
H	-6.97939300	0.76802100	-2.80356500
N	-2.73720800	1.11442400	-0.09132500
H	-2.08131000	0.73941600	-0.77015400

TS1

Thermal correction to Gibbs Free Energy= 0.431800 Hartree

SCF Done: E(RM06) = -2010.73743710 Hartree

0 1

Pd	2.10079400	-1.13272100	-0.58626900
O	1.36978200	0.03794800	2.33465200
C	2.30942900	-0.82526900	2.33301100
O	2.70852000	-1.52139800	1.35988700
C	3.00136800	-1.04689600	3.66599800
H	3.95584200	-1.55234500	3.51884800
H	3.14425100	-0.09287900	4.17772000
H	2.35778900	-1.66959300	4.29610400
Pd	-0.06717600	0.20936800	0.76895000
C	4.60981600	0.04048500	-1.06320800
O	3.41005700	0.40083700	-0.64483800
O	4.92638800	-1.09290700	-1.39272100
C	5.58856400	1.20985500	-1.09820400
H	6.56066100	0.86294500	-1.45002100
H	5.21435400	1.99388200	-1.76395400
H	5.69426200	1.64548800	-0.09934600
C	0.48975500	-0.10440600	-2.75290700
O	-0.28541900	0.36413100	-1.87851500
O	1.55881100	-0.76176300	-2.52673900
C	-0.26483400	-2.69047400	0.32240700
O	0.71548300	-2.68969500	-0.46267700
O	-0.69031000	-1.69853000	1.02010400
C	-1.03141800	-3.98167600	0.50799800
H	-0.61785900	-4.51131300	1.37326700
H	-2.08474900	-3.77175700	0.70166800
H	-0.91120500	-4.61510200	-0.37162200
C	0.14152200	0.08241300	-4.22096900
H	-0.25377900	1.08757000	-4.38560800
H	1.00909900	-0.09615800	-4.85650000
H	-0.64361300	-0.63322700	-4.48801200
C	-0.68963500	3.00981100	0.34566200
C	-0.51206400	4.38104300	0.19485200
C	0.78623800	4.88814600	0.15499400
C	1.85987100	4.00843400	0.27467900
C	1.61702300	2.64676200	0.45290600
H	-1.38803600	5.01412700	0.11781500
H	0.95527800	5.95282100	0.02816100
H	2.88503100	4.35863000	0.23073900
H	2.41558500	1.91454800	0.51550200
N	0.36105100	2.17458000	0.49360900
C	-2.07538600	2.42323200	0.41223100
O	-2.99285100	3.06121000	0.89561300
C	-3.52229500	0.47018200	0.04294400
C	-4.06502400	-0.01732300	-1.32246900
H	-4.18791700	1.26194900	0.40685800

C	-5.59199500	-0.24507500	-1.24076500
H	-3.90630500	0.83444300	-2.00162600
C	-6.26566700	-0.43919800	-2.60385500
H	-5.78891600	-1.11075900	-0.60061500
H	-6.05005200	0.62202200	-0.74540200
H	-5.92473600	-1.35021500	-3.10468100
C	-3.52329500	-0.58424800	1.16168200
O	-4.02868900	-1.68445200	1.09359000
O	-2.95812700	-0.08578800	2.27395900
C	-3.31514800	-1.23036900	-1.89866700
H	-3.50744100	-2.12232300	-1.29863200
H	-2.23666100	-1.05735500	-1.93683100
H	-3.64637800	-1.42781300	-2.92211700
C	-2.88211100	-0.98513100	3.39723400
H	-2.48223400	-0.39078200	4.21792100
H	-2.20999500	-1.81018700	3.15594600
H	-3.87354100	-1.37142500	3.64317600
H	-7.35127100	-0.51824500	-2.48798100
H	-6.06479200	0.40563300	-3.27337100
N	-2.18712100	1.10992700	-0.06764900
H	-1.68406800	0.93159500	-0.95428900

4

Thermal correction to Gibbs Free Energy= 0.430326 Hartree

SCF Done: E(RM06) = -2010.74788084 Hartree

0 1

Pd	1.63493400	-0.89320000	-1.08409600
O	1.43431400	-1.21257700	2.19707200
C	2.26466100	-1.99906200	1.61802300
O	2.45348800	-2.12059400	0.38281400
C	3.08089300	-2.88164600	2.54119300
H	3.97968000	-3.23022000	2.03225400
H	3.33595800	-2.34254300	3.45569600
H	2.47306000	-3.74806600	2.82269100
Pd	-0.08093500	-0.22728500	1.19559100
C	4.18321200	0.27523200	-1.39889400
O	3.06734800	0.47591800	-0.72506800
O	4.39515600	-0.65324200	-2.16488100
C	5.22207000	1.36112000	-1.12498900
H	6.14976600	1.11964800	-1.64497000
H	4.84836300	2.32946400	-1.47266200
H	5.41279800	1.44841700	-0.05065600
C	0.31858100	1.25072600	-2.68952200
O	-0.33039100	1.72763600	-1.72342900
O	1.02919300	0.19080900	-2.70113400
C	-0.72364600	-2.63061500	-0.40998300
O	0.09410500	-2.30627300	-1.30771900
O	-0.94262700	-2.01695600	0.69894900
C	-1.55995000	-3.87483200	-0.62101600
H	-1.20709800	-4.65695800	0.05944000
H	-2.60280600	-3.66077300	-0.37623600
H	-1.46367700	-4.22420900	-1.64878600
C	0.25759600	1.99049900	-4.01781700
H	0.53883900	3.03530200	-3.85702300
H	0.91538200	1.53579800	-4.75805900
H	-0.77374100	1.98363800	-4.38425200
C	-0.13780000	2.63334000	1.47947000
C	0.24943000	3.94791400	1.70915100
C	1.58220400	4.19687100	2.03672500
C	2.46916400	3.12594200	2.13205300
C	2.00585100	1.82931600	1.91204400
H	-0.48984900	4.73552100	1.62451500
H	1.92372800	5.21223000	2.21058500
H	3.51557600	3.28074800	2.36948600
H	2.64566200	0.95987900	1.96528200
N	0.72246000	1.60357200	1.60119800
C	-1.54442800	2.27392400	1.12062400
O	-2.48160500	2.97955300	1.42342200
C	-3.07613500	0.61462000	0.15753700
C	-3.45954600	0.62244500	-1.34371200
H	-3.67645700	1.39552700	0.63798800
C	-4.99564300	0.72497400	-1.49045600
H	-3.03326800	1.55864600	-1.73001700
C	-5.46506000	1.02882300	-2.91775000
H	-5.45188800	-0.20656300	-1.13954300
H	-5.36014000	1.52202600	-0.82780600
H	-5.22581600	0.21671500	-3.61064200
C	-3.43572500	-0.65568700	0.93355600
O	-4.08696200	-1.58309600	0.50380300
O	-3.02611800	-0.55603600	2.21239100
C	-2.86810800	-0.53261800	-2.16494100
H	-3.32759200	-1.48335600	-1.88572000
H	-1.78720000	-0.61788300	-2.04088700
H	-3.04981100	-0.36809200	-3.23073600
C	-3.27775200	-1.70938800	3.03863100
H	-2.94079200	-1.43185400	4.03680100
H	-2.70525900	-2.55805800	2.65997500
H	-4.34266300	-1.95115300	3.04151800

H	-6.55033800	1.17005300	-2.94340100
H	-5.00216100	1.94484600	-3.30331800
N	-1.65321800	1.03074100	0.40137900
H	-1.10333500	1.18089700	-0.52764300

TS2

Thermal correction to Gibbs Free Energy= 0.427975 Hartree

SCF Done: E(RM06) = -2010.74329028 Hartree

0 1

Pd	-1.60090400	-1.02516700	1.00284600
O	-1.47140400	-1.02888200	-2.27076600
C	-2.30728500	-1.84878900	-1.75844500
O	-2.49558600	-2.07751100	-0.53418700
C	-3.14371500	-2.64268800	-2.74228800
H	-2.55474900	-3.49866900	-3.08847900
H	-4.04953500	-3.01045300	-2.25992800
H	-3.38717800	-2.03122000	-3.61331100
Pd	0.05565500	-0.10468700	-1.20670800
C	-4.08813700	0.17590700	1.55794600
O	-3.00785200	0.40262200	0.83539800
O	-4.27087400	-0.79696000	2.27518100
C	-5.12393700	1.28729400	1.40560000
H	-5.99586800	1.06116800	2.02028900
H	-4.69508600	2.24690900	1.71033000
H	-5.42746200	1.38088100	0.35799900
C	-0.20978700	0.99292500	2.78138500
O	0.45510600	1.59142600	1.86851300
O	-0.87444700	-0.07209100	2.67900500
C	0.72888500	-2.67957900	0.13270700
O	-0.10329400	-2.48146300	1.05791600
O	0.94460700	-1.93038500	-0.88427800
C	1.57694200	-3.93158500	0.19942200
H	1.22066000	-4.63885800	-0.55706500
H	2.61426100	-3.68182600	-0.03417900
H	1.49682600	-4.39201500	1.18398000
C	-0.17138800	1.63866300	4.15203600
H	-0.86757900	1.14855600	4.83163500
H	0.84575600	1.57084200	4.55088400
H	-0.41441300	2.70059800	4.05830900
C	0.08782500	2.76047500	-1.23129200
C	-0.30265600	4.08758100	-1.36796900
C	-1.61870000	4.35677500	-1.74127400
C	-2.49015400	3.29286800	-1.97377400
C	-2.02566800	1.98617600	-1.83865000
H	0.42699100	4.86648000	-1.18076100
H	-1.96066000	5.38140700	-1.84735100
H	-3.52340500	3.46209400	-2.25529300
H	-2.64906900	1.11902300	-2.00720400
N	-0.75740900	1.74213800	-1.48014000
C	1.48145200	2.37725900	-0.83549000
O	2.40320300	3.16140200	-0.96417300
C	3.00484500	0.63170200	-0.11323200
C	3.43038000	0.46698800	1.36883100
H	3.59739900	1.46692900	-0.50475800
C	4.96933700	0.56676500	1.48925000
H	3.01314700	1.34960900	1.87149500
C	5.47254500	0.71617100	2.92964300
H	5.42415300	-0.31526300	1.02672800
H	5.31098800	1.43599600	0.91073000
H	5.25580800	-0.16908000	3.53511800
C	3.37417600	-0.53382600	-1.03455300
O	4.04430800	-1.49555900	-0.72371400
O	2.95414700	-0.29490600	-2.29294400
C	2.87311500	-0.78189800	2.06778400
H	3.33892800	-1.68709300	1.67190400
H	1.79134200	-0.87500500	1.95578300
H	3.07719400	-0.73867200	3.14167300
C	3.22434100	-1.33965600	-3.24590500
H	2.86537000	-0.96196700	-4.20283900
H	2.68204000	-2.24294600	-2.96000200
H	4.29519000	-1.55003200	-3.28942200
H	6.55731300	0.86341400	2.94516600
H	5.01397300	1.58044600	3.42468500
N	1.57824200	1.06694500	-0.29110200
H	0.95545600	1.16971800	0.80223600

2

Thermal correction to Gibbs Free Energy= 0.375102 Hartree

SCF Done: E(RM06) = -1781.70773891 Hartree

0 1

Pd	-1.72008700	-1.20103000	0.69371700
O	-1.41808800	-0.40829100	-2.44725800
C	-2.27840300	-1.30863100	-2.19820900
O	-2.52322000	-1.83931200	-1.07605500
C	-3.08520800	-1.82213800	-3.37433000
H	-2.47270700	-2.53966000	-3.93039300
H	-3.99163300	-2.32048300	-3.03068300
H	-3.32750500	-0.99894500	-4.04961500

Pd	0.15691900	0.20517100	-1.20285100
C	-4.20872500	-0.09338300	1.40645500
O	-3.09724600	0.25485300	0.78450800
O	-4.41260900	-1.17835200	1.93199300
C	-5.25021000	1.02098400	1.39009900
H	-6.07260200	0.76180000	2.05796800
H	-4.80546400	1.97370100	1.69083800
H	-5.63780300	1.14492000	0.37347100
C	-0.57684500	0.27604800	3.11677700
O	0.14772200	1.11729500	2.40271700
O	-1.15424500	-0.71929200	2.67144500
C	0.64172500	-2.66008300	-0.37354300
O	-0.25101100	-2.65241400	0.52382000
O	0.92848200	-1.71736400	-1.18247700
C	1.46673600	-3.92080500	-0.51919600
H	1.16473800	-4.43361800	-1.43863700
H	2.52147300	-3.65262100	-0.61275000
H	1.30341500	-4.58503000	0.32930100
C	-0.63121900	0.62378800	4.57612900
H	-1.32413600	-0.04126800	5.08857900
H	0.37020900	0.53082200	5.00735100
H	-0.94328900	1.66533500	4.69230600
C	0.34602700	3.00385700	-0.64267700
C	0.03456200	4.35485700	-0.53875800
C	-1.20281000	4.79401100	-1.00507600
C	-2.08740400	3.86933400	-1.56353000
C	-1.71016900	2.53185000	-1.64551100
H	0.77542600	5.01368300	-0.10062800
H	-1.47649100	5.84228000	-0.93504600
H	-3.06104600	4.17116000	-1.93318900
H	-2.34338200	1.76295000	-2.06880300
N	-0.51361900	2.12578600	-1.19369600
C	1.66602600	2.45006900	-0.18227100
O	2.56106600	3.21737100	0.17829900
C	3.08799000	0.54370800	-0.00479600
C	3.39399900	0.16764500	1.47110800
H	3.76182100	1.38005600	-0.23891200
C	4.91874100	0.16072200	1.72200400
H	2.98032100	1.00692100	2.04594800
C	5.30900600	0.07723500	3.20232700
H	5.36932900	-0.67241200	1.17200500
H	5.34330400	1.08251100	1.30232000
H	5.00296300	-0.87065200	3.65613600
C	3.45764200	-0.52360600	-1.03572200
O	4.03699000	-1.56536100	-0.80205800
O	3.15723700	-0.10989200	-2.28301100
C	2.72096300	-1.12410600	1.95636900
H	3.19589100	-2.00116700	1.51106400
H	1.65876500	-1.15640200	1.70420500
H	2.79665800	-1.21365400	3.04463700
C	3.44484500	-1.04843500	-3.33250800
H	3.20348100	-0.53207300	-4.26142900
H	2.81927700	-1.93571300	-3.21381200
H	4.49807800	-1.33799300	-3.31363500
H	6.39438400	0.15909700	3.32152400
H	4.85108800	0.88868500	3.78066000
N	1.72977700	1.08492300	-0.21704500
H	0.29382100	0.81088300	1.47834400

TS3

Thermal correction to Gibbs Free Energy= 0.375961 Hartree

SCF Done: E(RM06) = -1781.68951106 Hartree

0 1

C	-0.50983600	2.95221300	-0.21773900
C	-1.15002800	4.17685800	-0.06421000
C	-2.39291900	4.36381400	-0.66334500
C	-2.95787900	3.32057400	-1.40081700
C	-2.26351200	2.12014300	-1.51598400
H	-0.64824500	4.93685100	0.52366100
H	-2.91737800	5.30882000	-0.55966100
H	-3.92304000	3.42751600	-1.88360000
H	-2.63743500	1.27173700	-2.07680200
N	-1.06761500	1.95683600	-0.93042900
C	0.82845800	2.66885100	0.40589200
O	1.38871200	3.53446600	1.08450500
N	1.27678900	1.41530200	0.14487100
Pd	0.06981100	0.27491300	-1.01076300
C	-1.88782000	-1.82621300	-2.08337900
O	-1.92435500	-2.50831800	-1.01215500
O	-1.30937500	-0.71643400	-2.27819300
C	-2.60727500	-2.43768300	-3.27294500
H	-3.32110900	-3.19438900	-2.94813900
H	-1.86225900	-2.90493800	-3.92526900
H	-3.10774500	-1.65451700	-3.84613500
C	2.52957800	1.01004400	0.77792100
C	2.36180800	0.00265400	1.95461700
H	2.96153000	1.93727400	1.18108800
C	1.17089000	0.35693800	2.87832300

H	2.16450300	-0.97887200	1.51244000
C	0.45608300	-0.87465700	3.43684700
H	1.52941200	0.96933500	3.71591700
H	0.42502000	0.95253600	2.35417000
H	1.13523400	-1.59922400	3.89965500
C	3.52625500	0.51329100	-0.27739800
O	4.22897200	-0.47474700	-0.18608300
O	3.57612200	1.34815000	-1.33204000
C	3.65749300	-0.10700400	2.77849200
H	3.93046000	0.86965300	3.19701200
H	4.49049400	-0.47508900	2.17862800
H	3.51440000	-0.79379700	3.62013000
C	4.48361700	0.96574200	-2.37713300
H	4.41745300	1.75462400	-3.12615000
H	4.18601900	0.00525500	-2.80519600
H	5.50379500	0.88406300	-1.99347800
H	-0.32045900	-0.60245900	4.15586500
C	1.45878200	-2.35959800	-0.57778000
O	0.64408800	-2.71622600	0.33303800
O	1.40805600	-1.28465000	-1.24586900
C	2.61361600	-3.28702600	-0.87028900
H	2.64403600	-3.49940900	-1.94246600
H	2.52164300	-4.21388200	-0.30470900
H	3.53732500	-2.76377100	-0.60628500
Pd	-1.11894600	-1.78280500	0.77145000
C	-2.97204700	0.01573900	1.80566600
O	-2.95118800	-1.18100000	1.27030900
C	-4.35218300	0.45492500	2.25754900
H	-4.31674800	1.49287400	2.59082500
H	-4.68819500	-0.18174100	3.08202600
H	-5.07445400	0.34125100	1.44441500
O	-1.96694700	0.71479100	1.94155500
H	-0.05943900	-1.43749100	2.62310900

5

Thermal correction to Gibbs Free Energy= 0.375806 Hartree

SCF Done: E(RM06) = -1781.69222436 Hartree

0 1

C	-0.66138900	2.81956100	-0.34825100
C	-1.37625600	4.00732800	-0.25084200
C	-2.58741400	4.11805700	-0.92842200
C	-3.04659200	3.03750500	-1.68465600
C	-2.28108100	1.87681200	-1.73958700
H	-0.95567400	4.80126100	0.35537900
H	-3.16917600	5.03287800	-0.86960800
H	-3.98501300	3.08450100	-2.22617400
H	-2.57563300	1.00023000	-2.30473300
N	-1.11570700	1.78875100	-1.08267800
C	0.65965900	2.62524300	0.34073900
O	1.15078000	3.54026400	1.01074400
N	1.19262000	1.39486900	0.13132000
Pd	0.09378700	0.16020800	-1.03736200
C	-1.78064000	-2.09727400	-1.86118800
O	-1.80167300	-2.63000500	-0.70558500
O	-1.22829100	-1.01207900	-2.20974300
C	-2.48473400	-2.88542800	-2.95120800
H	-2.96156600	-2.20179700	-3.65621900
H	-3.21508600	-3.57176700	-2.52273600
H	-1.73412300	-3.46399400	-3.49990800
C	2.52178200	1.14801800	0.68591800
C	2.58406700	0.29988800	1.99340200
H	2.91333000	2.14539900	0.93504900
C	1.42909500	0.60163000	2.98571800
H	2.53939700	-0.75895600	1.71750600
C	0.26181100	-0.40197800	2.99311200
H	1.84625900	0.58584800	4.00045000
H	1.04570300	1.61463800	2.83364000
H	0.62464200	-1.43048900	3.07262200
C	3.46532700	0.59710000	-0.39130100
O	4.26698300	-0.30359700	-0.23537500
O	3.34579500	1.27890600	-1.54493100
C	3.93653700	0.54435700	2.69138900
H	4.00488900	1.58037900	3.04532600
H	4.77574300	0.34702400	2.02275900
H	4.03872400	-0.11076700	3.56309400
C	4.17998500	0.81874600	-2.62024500
H	3.97834400	1.48943200	-3.45520200
H	3.92070900	-0.20966200	-2.88302500
H	5.23473400	0.86180700	-2.33749100
H	-0.43283800	-0.21031100	3.81503400
C	1.58908700	-2.34213700	-0.32825600
O	0.79026300	-2.61088800	0.62442200
O	1.49509000	-1.36260200	-1.12643700
C	2.77619000	-3.25762400	-0.51408900
H	2.73360400	-4.09664500	0.17990300
H	3.68413000	-2.66900900	-0.35384900
H	2.79188800	-3.62221300	-1.54499300
Pd	-1.00935900	-1.68010100	0.95474000

C	-3.12406000	0.13093400	1.68380300
O	-2.86652000	-1.13231100	1.43054300
C	-4.57932100	0.34844300	2.07811000
H	-4.76040700	1.41244500	2.23433200
H	-4.80099600	-0.20240700	2.99705300
H	-5.24431500	-0.03774400	1.30044700
O	-2.31089100	1.04921900	1.62738600
H	-0.34397300	-0.21071500	2.06841000

TS2-δ

Thermal correction to Gibbs Free Energy= 0.375559 Hartree

SCF Done: E(RM06) = -1781.67450173 Hartree

0 1

C	1.09223500	-2.67139800	-0.35094600
C	1.95577600	-3.75675800	-0.24466700
C	3.16863600	-3.71905000	-0.92836300
C	3.48311400	-2.59663900	-1.70000700
C	2.57471500	-1.54377200	-1.76341700
H	1.64110400	-4.59152700	0.37111200
H	3.86158800	-4.55270000	-0.86578700
H	4.41560700	-2.53202600	-2.25000700
H	2.74740700	-0.64299900	-2.34157200
N	1.41079000	-1.59613800	-1.09641900
C	-0.24170400	-2.64194300	0.34513600
O	-0.59133800	-3.59991200	1.04374300
N	-0.93423000	-1.50196700	0.10769600
Pd	-0.02157300	-0.15059600	-1.09051700
C	1.51203000	2.30917300	-1.86673600
O	1.40383900	2.80065800	-0.70611700
O	1.13272300	1.15897800	-2.26357700
C	2.13984700	3.20471300	-2.91973400
H	2.65618500	2.60569600	-3.67189000
H	2.82146900	3.91907900	-2.45666200
H	1.34152100	3.76218600	-3.42144100
C	-2.26279900	-1.39689400	0.70144200
C	-2.39978500	-0.46118000	1.94302900
H	-2.50511200	-2.41610700	1.03662700
C	-1.16340000	-0.46466200	2.88211700
H	-2.56019500	0.55809600	1.58414600
C	-0.24812200	0.78550200	2.82984600
H	-1.53605800	-0.50516500	3.91438200
H	-0.58125700	-1.38120000	2.74901500
H	-0.86415900	1.68388600	2.76703700
C	-3.30925100	-1.05974200	-0.36924400
O	-4.22886600	-0.27589600	-0.23527500
O	-3.12661400	-1.78996800	-1.48472300
C	-3.65458900	-0.87158900	2.73917000
H	-3.52569900	-1.86867000	3.17792900
H	-4.54406300	-0.88061300	2.10647400
H	-3.83297800	-0.16801800	3.55942000
C	-4.05765100	-1.53135700	-2.54752000
H	-3.78451600	-2.21660600	-3.34976200
H	-3.96962800	-0.49492500	-2.88226500
H	-5.08310600	-1.71473500	-2.21692800
H	0.33440800	0.88303200	3.75376000
C	-1.88166000	2.12050200	-0.43168300
O	-1.19185500	2.48625500	0.57030700
O	-1.61727400	1.16518600	-1.22279100
C	-3.16806000	2.87094500	-0.69366600
H	-3.25338000	3.73759700	-0.03861200
H	-3.99837900	2.17936200	-0.52188300
H	-3.20156000	3.18193600	-1.74106600
Pd	0.69849800	1.75660800	0.97994600
C	2.83577900	0.11921900	1.89308100
O	2.66349100	1.27283800	1.38529400
C	4.25277000	-0.37564700	2.06270300
H	4.40676800	-1.23731100	1.40595800
H	4.39689900	-0.71735900	3.09065800
H	4.97062500	0.40634000	1.81739200
O	1.87935800	-0.62873300	2.25373500
H	0.81700500	0.18115600	2.22329200

6

Thermal correction to Gibbs Free Energy= 0.378340 Hartree

SCF Done: E(RM06) = -1781.71930082 Hartree

0 1

C	0.09843100	2.98982200	0.64471000
C	-0.30894700	4.22777300	1.13215500
C	-1.37477100	4.87638100	0.51377100
C	-1.99992000	4.26905300	-0.57825300
C	-1.54385800	3.02848800	-1.01375500
H	0.23189200	4.63657100	1.97806400
H	-1.71315600	5.84415000	0.87139500
H	-2.82736400	4.74531200	-1.09280900
H	-1.97349000	2.50719600	-1.86128400
N	-0.52138700	2.40987200	-0.40172700
C	1.25407800	2.23985900	1.25171100
O	1.84671000	2.71617200	2.22682700

N	1.50917900	1.06890800	0.62213900
Pd	0.29651000	0.61209400	-0.93035300
C	-2.06703800	-0.46931800	-2.40139200
O	-2.33207800	-1.19820100	-1.39533900
O	-1.08076000	0.32096800	-2.50244000
C	-3.00269200	-0.55593400	-3.59324100
H	-2.73416600	0.16854700	-4.36203700
H	-4.03471400	-0.39476100	-3.26708900
H	-2.94997300	-1.56665000	-4.00953700
C	2.61081000	0.26156400	1.13262900
C	2.20657100	-1.05341300	1.86993900
H	3.12910800	0.90916000	1.85450400
C	0.86682300	-0.96119000	2.64937100
H	2.09785200	-1.82814000	1.10913400
C	-0.15705600	-2.02980000	2.27681700
H	1.07586600	-1.07408000	3.72531600
H	0.41455700	0.03007000	2.55229500
H	0.29211800	-3.01995400	2.14785300
C	3.63414700	-0.02238000	0.02412700
O	4.20882200	-1.07889600	-0.15125200
O	3.87504500	1.07413800	-0.71922500
C	3.34686300	-1.49402400	2.80542600
H	3.50505200	-0.75584500	3.60148200
H	4.28316900	-1.62566400	2.25910100
H	3.09898100	-2.44844600	3.28304100
C	4.81627000	0.89437800	-1.78836800
H	4.91181500	1.87100000	-2.26281800
H	4.44110700	0.15699100	-2.50232000
H	5.78195800	0.55798100	-1.40235400
H	-0.97961200	-2.07444100	2.99821700
C	1.21361000	-2.24206700	-1.28379400
O	0.36604600	-2.72324300	-0.47985500
O	1.37631400	-1.01636200	-1.58605000
C	2.18178500	-3.20480700	-1.93982000
H	1.89564700	-4.23741100	-1.74051800
H	3.17737800	-3.00443500	-1.53153500
H	2.21812800	-3.01961200	-3.01631100
Pd	-1.10790800	-1.64161600	0.53028500
C	-3.91973200	-0.73124400	1.35405500
O	-2.73321300	-0.83758800	1.69664500
C	-4.98399000	-0.36692300	2.35384700
H	-5.67801600	-1.20659300	2.46067000
H	-5.56068400	0.48783100	1.99025400
H	-4.53049000	-0.13902700	3.31733500
O	-4.37313200	-0.92424400	0.13765800
H	-3.62153400	-1.11243400	-0.51517000

3-δ

Thermal correction to Gibbs Free Energy= 0.496549 Hartree

SCF Done: E(RM06) = -2091.85790790 Hartree

0 1

C	-2.67527500	2.30082500	-0.32402200
C	-3.12777800	3.59182900	-0.57790800
C	-2.83158200	4.59957300	0.33795300
C	-2.08968000	4.28732800	1.47934200
C	-1.66661900	2.97531000	1.67340900
H	-3.70251700	3.75448200	-1.48266300
H	-3.17636800	5.61531800	0.16883500
H	-1.83971600	5.04350900	2.21530600
H	-1.10177300	2.65332500	2.54064400
N	-1.95748400	2.01662900	0.78066900
C	-2.96146400	1.15769600	-1.26394000
O	-3.56925700	1.37518900	-2.31881100
N	-2.47242500	-0.02351400	-0.81763300
Pd	-1.55206000	0.02686600	0.99477000
C	0.60118300	0.11626300	3.02094300
O	1.44035500	-0.19547700	2.14938800
O	-0.65516700	0.33136200	2.84282800
C	1.07097300	0.24169100	4.46268300
H	2.12215500	0.53270800	4.49291600
H	0.97202200	-0.73636800	4.94619400
H	0.45588500	0.95354400	5.01634200
C	-2.68888200	-1.19519500	-1.65793800
C	-1.40006400	-1.82346800	-2.27244600
H	-3.33150000	-0.85002200	-2.48065600
C	-0.30450900	-0.78489400	-2.63131700
H	-0.98501800	-2.50316400	-1.52771900
H	-0.20057500	-0.74164600	-3.72720500
H	-0.61037700	0.22438400	-2.34130100
C	-3.50906600	-2.25377100	-0.90632000
O	-3.29774500	-3.45085500	-0.91905700
O	-4.54756000	-1.70008700	-0.25358700
C	-1.76477900	-2.66835600	-3.50610500
H	-2.19007400	-2.04002700	-4.29864900
H	-2.48543500	-3.44896000	-3.25415300
H	-0.87074800	-3.15455900	-3.91230300
C	-5.36525900	-2.61727300	0.48926800
H	-6.16459000	-2.01484400	0.92061400

H	-4.77778500	-3.09435400	1.27760400
H	-5.77602600	-3.38899600	-0.16676800
C	-0.61295100	-2.83403100	1.00494400
O	0.45398600	-2.57831000	0.38685700
O	-1.52665500	-2.00109800	1.31628400
C	-0.89965100	-4.27399700	1.37916400
H	-0.00810000	-4.88924800	1.25706900
H	-1.69446300	-4.63545100	0.71893900
H	-1.26500300	-4.32877000	2.40736600
Pd	1.24334300	-0.61747700	-0.08668100
C	1.08624200	-1.09004700	-2.07580800
H	1.32354600	-2.15850800	-2.09367700
H	1.86169400	-0.52820600	-2.60007000
C	3.26185500	0.07014500	-0.36736200
C	2.52513100	1.07726900	-0.52264500
C	4.41633000	-0.78304000	-0.31549700
C	4.32812800	-2.13328500	0.07223100
C	5.67525200	-0.24672000	-0.65925100
C	5.47441200	-2.92338200	0.11390600
H	3.36036200	-2.54603300	0.33843500
C	6.81366900	-1.04526100	-0.61066800
H	5.74662400	0.79221700	-0.96585100
C	6.71795300	-2.38523100	-0.22480800
H	5.39515700	-3.96403000	0.41400300
H	7.77764800	-0.62202100	-0.87766800
H	7.60844600	-3.00587000	-0.18960400
C	2.24019700	2.47110000	-0.74159300
C	3.26071900	3.42127600	-0.52622600
C	0.98498000	2.91388800	-1.19367500
C	3.02635500	4.77213000	-0.76416200
H	4.22902800	3.08709500	-0.16710400
C	0.76102500	4.26738900	-1.43734300
H	0.19799300	2.18733800	-1.35869600
C	1.77755200	5.20070000	-1.22338400
H	3.82092200	5.49264100	-0.59274800
H	-0.21109300	4.59122700	-1.79642400
H	1.59995600	6.25503800	-1.41412700

TS3-δ

Thermal correction to Gibbs Free Energy= 0.499273 Hartree

SCF Done: E(RM06) = -2091.82455393 Hartree

0 1

C	0.12581000	0.32320900	2.73406200
C	0.97539200	0.60111900	3.80028700
C	1.74500100	-0.43101600	4.33370000
C	1.64653600	-1.70990100	3.78181200
C	0.77882200	-1.91974900	2.71332600
H	0.99645900	1.61736400	4.17698300
H	2.41286500	-0.24384700	5.16918500
H	2.23070500	-2.53854900	4.16639200
H	0.64951600	-2.88343000	2.23409000
N	0.04153700	-0.91653900	2.21385400
C	-0.74636800	1.38254300	2.11176000
O	-0.64295600	2.55827100	2.49838000
N	-1.56487100	0.90033900	1.15361200
Pd	-1.30168400	-1.06185300	0.68627500
C	-0.13867200	-3.36383500	-0.75936700
O	0.34805500	-2.58461400	-1.62193200
O	-0.79433200	-3.04899600	0.29061900
C	0.09858500	-4.84948100	-0.97643600
H	1.17041600	-5.05453300	-0.88859100
H	-0.20425200	-5.12050700	-1.99109500
H	-0.45069600	-5.44774800	-0.24928100
C	-2.51844200	1.82735900	0.55035500
C	-2.15506000	2.35931200	-0.87038500
H	-2.55535500	2.69461700	1.22600600
C	-0.64500000	2.69288200	-1.04083400
H	-2.42874600	1.59097000	-1.59565100
H	-0.57344000	3.71984100	-1.42492100
H	-0.15732600	2.73719300	-0.06585900
C	-3.92794900	1.21595900	0.57323400
O	-4.73699100	1.26771200	-0.33162600
O	-4.19615300	0.64572500	1.76282200
C	-2.99384100	3.61505800	-1.18004500
H	-2.73253100	4.43385800	-0.49790400
H	-4.06246000	3.41451000	-1.09315800
H	-2.79657200	3.95964600	-2.20106800
C	-5.48219800	0.01364200	1.86161600
H	-5.54095900	-0.37435000	2.87845500
H	-5.55735800	-0.79938000	1.13557200
H	-6.28337700	0.73396500	1.67747600
C	-2.75286900	-0.97850800	-1.91956500
O	-1.72314100	-0.61239000	-2.54115100
O	-2.84296000	-1.20399100	-0.66273000
C	-4.05081500	-1.13882300	-2.68609200
H	-3.87738100	-1.05237900	-3.75883500
H	-4.73924600	-0.35686600	-2.35088500
H	-4.50531700	-2.10497300	-2.45174300

Pd	0.29527300	-0.42297500	-1.68175300
C	0.14438400	1.83188300	-2.02838800
H	-0.49067700	1.37598700	-2.79719300
H	0.90751000	2.38864400	-2.56400800
C	2.14138500	-0.08887500	-0.99709600
C	1.96537500	1.18211600	-1.00568800
C	3.17568400	-1.03393800	-0.57506200
C	3.38303300	-2.24572200	-1.25614500
C	4.00701300	-0.71339800	0.51540500
C	4.40759300	-3.10597000	-0.86225900
H	2.72922400	-2.50607200	-2.07963200
C	5.02391900	-1.58153700	0.90711400
H	3.85011100	0.22015800	1.04653200
C	5.22948900	-2.77990700	0.21873800
H	4.56426500	-4.03499900	-1.40324700
H	5.65973400	-1.31969200	1.74834900
H	6.02474800	-3.45434800	0.52325600
C	2.57277800	2.47541700	-0.73672600
C	2.39211400	3.12558300	0.49864900
C	3.39460000	3.07379800	-1.71214700
C	3.03058400	4.34046100	0.74910600
H	1.74389100	2.69317600	1.25326400
C	4.03179000	4.28404300	-1.45099500
H	3.53567900	2.57296700	-2.66524900
C	3.85006900	4.92091400	-0.22015700
H	2.87957700	4.83503100	1.70395200
H	4.67085700	4.73028400	-2.20729000
H	4.34375500	5.86741000	-0.01987700

4-δ

Thermal correction to Gibbs Free Energy= 0.501213 Hartree

SCF Done: E(RM06) = -2091.87088831 Hartree

0 1

C	-0.92447300	-0.42409800	3.08240500
C	-0.46739700	-0.57367500	4.38753100
C	0.18027100	-1.75496000	4.74183700
C	0.35387200	-2.75101100	3.77845500
C	-0.12523900	-2.53631600	2.48925300
H	-0.63597700	0.24608500	5.07654500
H	0.54526800	-1.90051500	5.75403100
H	0.85231100	-3.68491000	4.01368800
H	-0.03588500	-3.27080400	1.69820100
N	-0.74470900	-1.39189500	2.16069100
C	-1.60081500	0.83938600	2.61837400
O	-1.69190100	1.80355400	3.38618900
N	-2.00742200	0.77214400	1.32824200
Pd	-1.62668100	-0.96944300	0.36184600
C	-0.19104200	-3.10777100	-1.27122500
O	0.61850000	-2.29788500	-1.81324000
O	-1.11941300	-2.84991400	-0.44382500
C	-0.02430100	-4.56373000	-1.67289700
H	1.03687700	-4.81813900	-1.71482600
H	-0.43870600	-4.69713900	-2.67750100
H	-0.55313700	-5.22120200	-0.98217500
C	-2.48477100	2.00308900	0.71027600
C	-1.49873400	2.60306400	-0.34300900
H	-2.60143000	2.72272800	1.53307200
C	-0.02024900	2.45389900	0.08900900
H	-1.64649300	2.02572800	-1.26249600
H	0.27925400	3.32779200	0.67932500
H	0.08682400	1.58886200	0.74275600
C	-3.88675400	1.80981500	0.11636200
O	-4.26291100	2.23270800	-0.95952400
O	-4.68957000	1.15165700	0.97007900
C	-1.83548400	4.07201400	-0.64994700
H	-1.74382500	4.68707800	0.25371300
H	-2.84862800	4.17287300	-1.04092100
H	-1.14368600	4.47829700	-1.39667800
C	-6.02086900	0.90256400	0.49424700
H	-6.54091900	0.41551400	1.31897700
H	-5.99121800	0.24642200	-0.37910500
H	-6.51780400	1.83755900	0.22343200
C	-2.58290800	-0.36206900	-2.42670500
O	-1.42240700	-0.26733900	-2.89669300
O	-2.89223500	-0.56341200	-1.20005900
C	-3.76815500	-0.19367000	-3.35631300
H	-3.43631400	-0.11605200	-4.39173700
H	-4.30301000	0.71460500	-3.06189400
H	-4.45454300	-1.03686300	-3.24072800
Pd	0.44424600	-0.21458400	-1.74152100
C	0.96446600	2.24810700	-1.08819800
H	0.40705600	1.72890500	-1.92431100
H	1.21136800	3.18786000	-1.59695900
C	2.18491100	0.15467400	-0.85787100
C	2.25928500	1.49577000	-0.72298600
C	3.09266400	-0.92021700	-0.45158400
C	3.63158800	-1.82433300	-1.38674600
C	3.41834200	-1.08721400	0.90943800

C	4.48994200	-2.84242600	-0.97599500
H	3.37066600	-1.71895500	-2.43458300
C	4.27350400	-2.10979700	1.31579600
H	3.00405000	-0.39698600	1.63789300
C	4.81456800	-2.99006600	0.37517400
H	4.90607900	-3.52385800	-1.71277200
H	4.52186800	-2.21692900	2.36823900
H	5.48310000	-3.78535000	0.69240800
C	3.43905400	2.28328900	-0.28313900
C	3.28074900	3.55860600	0.29193500
C	4.75312100	1.81042900	-0.47064200
C	4.38347000	4.31699900	0.68490500
H	2.28719900	3.96422600	0.44656300
C	5.85293200	2.56917700	-0.07940100
H	4.91160000	0.84773300	-0.94188000
C	5.67622000	3.82583800	0.50499700
H	4.22776600	5.29426000	1.13330100
H	6.85442900	2.18074800	-0.24253100
H	6.53567100	4.41675500	0.80801800

TS4

Thermal correction to Gibbs Free Energy= 0.373871 Hartree

SCF Done: E(RM06) = -1781.66927153 Hartree

0 1

C	-0.65645400	2.72677900	-0.97619600
C	-1.24978500	3.98144400	-1.06408100
C	-2.56855000	4.07065800	-1.50059800
C	-3.25521300	2.90161700	-1.83881600
C	-2.60274100	1.67797100	-1.72713300
H	-0.65205100	4.84063900	-0.78218700
H	-3.05862600	5.03636200	-1.57868100
H	-4.28225400	2.92924300	-2.18584300
H	-3.07035900	0.73348000	-1.97797400
N	-1.33136300	1.61014500	-1.30165500
C	0.75957300	2.54593300	-0.51218900
O	1.42841700	3.52677000	-0.17029600
N	1.16606100	1.25241600	-0.52186300
Pd	-0.24165200	-0.09399000	-1.09590100
C	-2.54922200	-2.12163600	-1.01133500
O	-2.43798000	-2.37122900	0.22926400
O	-1.86991600	-1.31125500	-1.71023700
C	-3.66338400	-2.86956500	-1.72427900
H	-3.40101600	-3.02505400	-2.77174200
H	-4.57461300	-2.26322200	-1.68009700
H	-3.86251900	-3.82005400	-1.22840500
C	2.49813000	1.01958000	0.04561700
C	2.50195900	0.97152200	1.60051100
H	3.07462900	1.91347900	-0.22619900
C	3.92565200	1.03313700	2.20602500
H	1.98992400	1.89703500	1.88856800
H	3.80689900	1.07386100	3.29653500
H	4.45716300	0.10208700	1.98536600
C	3.22606200	-0.12351200	-0.65520700
O	3.77789400	-1.06024300	-0.10848200
O	3.26336800	0.08386700	-1.98429500
C	3.91561300	-0.93078700	-2.76152300
H	3.97415300	-0.53299000	-3.77462100
H	3.32069500	-1.84695600	-2.74884900
H	4.91422400	-1.13833200	-2.36982700
C	1.04365100	-2.69796300	-0.27539300
O	0.36836400	-2.76715500	0.79999500
O	0.99252800	-1.76514000	-1.13090500
C	2.02535900	-3.81568200	-0.53472200
H	3.03014800	-3.41293200	-0.37327100
H	1.95038300	-4.13894400	-1.57587600
H	1.84632900	-4.65349000	0.13896400
Pd	-1.01174100	-1.39121500	1.39568100
C	-2.21543900	0.96049600	2.36623100
O	-2.48594500	-0.31158700	2.18399500
C	-3.34533400	1.71765000	3.04232700
H	-3.09574300	2.77808300	3.09416800
H	-3.49774500	1.32685800	4.05310000
H	-4.28103500	1.57463800	2.49453600
O	-1.15654400	1.49214400	2.03630900
C	4.76881400	2.23652700	1.76266800
H	4.21634200	3.17584000	1.87510000
H	5.08258600	2.16170900	0.71616900
H	5.68073900	2.30748400	2.36485100
C	1.72228000	-0.19599800	2.22456100
H	0.65439400	-0.10600900	1.93032300
H	2.12154300	-1.16717500	1.93249400
H	1.74169900	-0.12254200	3.31656100

7

Thermal correction to Gibbs Free Energy= 0.375363 Hartree

SCF Done: E(RM06) = -1781.68740391 Hartree

0 1

C	-1.14051400	2.57021100	-1.12050300

C	-1.91676800	3.71922100	-1.22285200
C	-3.26262400	3.59197000	-1.55454200
C	-3.79272100	2.31813500	-1.77486600
C	-2.96053000	1.20967200	-1.65487500
H	-1.43412200	4.67081700	-1.03200900
H	-3.89395600	4.47104200	-1.64115300
H	-4.83554100	2.17706400	-2.03689400
H	-3.30142600	0.19420900	-1.81787900
N	-1.66545400	1.35070700	-1.33261100
C	0.31355300	2.62125000	-0.75198800
O	0.84783500	3.70209900	-0.48473600
N	0.90882000	1.40179700	-0.74620600
Pd	-0.31962400	-0.16274600	-1.15502200
C	-2.28089700	-2.47156700	-0.68613900
O	-1.99413900	-2.60942000	0.54465600
O	-1.79876400	-1.64391800	-1.51631300
C	-3.36664000	-3.40128600	-1.20161900
H	-3.23904800	-3.57312200	-2.27123800
H	-4.34013900	-2.92673200	-1.03702300
H	-3.35242500	-4.34344700	-0.65252100
C	2.27678700	1.38770300	-0.22309200
C	2.34985600	1.40141900	1.33202600
H	2.71115800	2.34735200	-0.53776400
C	3.78873900	1.70254500	1.81741300
H	1.72466400	2.25636200	1.61381900
H	4.41274300	0.81299000	1.68629500
H	4.21279100	2.47710900	1.16405500
C	3.14122900	0.33653700	-0.91412400
O	3.81550300	-0.51105900	-0.35879200
O	3.13198200	0.52155100	-2.24574500
C	3.89783600	-0.41357700	-3.01916000
H	3.89989200	-0.02188500	-4.03628600
H	3.41885600	-1.39503300	-2.99342100
H	4.91704900	-0.49354800	-2.63387100
C	1.42179800	-2.52382600	-0.44299800
O	0.89129500	-2.65212300	0.70541600
O	1.14908700	-1.62831200	-1.29536300
C	2.50884700	-3.50979200	-0.80004900
H	3.46677000	-2.99290700	-0.68459700
H	2.40615600	-3.81594800	-1.84355400
H	2.47696500	-4.37714300	-0.14060300
Pd	-0.55341700	-1.42750400	1.46697000
C	-2.06018800	0.78819000	2.47482700
O	-2.00981400	-0.52472800	2.48046800
C	-3.19405600	1.32552300	3.33624800
H	-3.20707600	2.41478200	3.28495300
H	-3.05783900	1.00515700	4.37337900
H	-4.15128900	0.92084100	2.99496300
O	-1.29035300	1.52070400	1.85889900
C	3.86879700	2.19790800	3.26625800
H	3.53572400	1.44013000	3.98309300
H	3.25137100	3.09094300	3.41485300
H	4.89954900	2.45842100	3.52853200
C	1.77412400	0.15849800	2.03405700
H	0.68059200	0.13393100	1.79218800
H	2.28189800	-0.75677200	1.73223900
H	1.83218100	0.25863300	3.12050900

TS2-γ

Thermal correction to Gibbs Free Energy= 0.373871 Hartree

SCF Done: E(RM06) = -1781.66927153 Hartree

0 1

C	1.83336800	-1.76174900	-1.76467600
C	2.76775000	-2.72172300	-2.13863400
C	4.11566400	-2.37141000	-2.16632500
C	4.49014100	-1.07270800	-1.81314100
C	3.50533700	-0.16143700	-1.44291500
H	2.40088600	-3.70980000	-2.39240000
H	4.86734700	-3.09861900	-2.45794100
H	5.52913600	-0.76217000	-1.82141300
H	3.72033100	0.86188400	-1.15733000
N	2.21072500	-0.51473800	-1.42350900
C	0.36232300	-2.06527700	-1.68247000
O	-0.04073600	-3.21929700	-1.87058300
N	-0.38519800	-0.98050000	-1.36677400
Pd	0.65338800	0.72166100	-0.99163700
C	2.04419400	2.55912800	0.86344900
O	1.43778400	2.03542900	1.84116000
O	1.92677800	2.27341900	-0.37482200
C	3.01306400	3.67768800	1.20531400
H	2.43708700	4.58602400	1.41065800
H	3.69313300	3.87257900	0.37535700
H	3.56847800	3.42575600	2.11093200
C	-1.80179200	-1.24262100	-1.11367600
C	-2.11864500	-1.73029200	0.33243600
H	-2.04841800	-2.09713300	-1.75955900
C	-3.52068300	-2.39988400	0.36906200
H	-1.38729600	-2.52804200	0.50708100

H	-4.29062400	-1.62174800	0.39666900
H	-3.66857800	-2.94850700	-0.57066900
C	-2.68607100	-0.10974800	-1.63014500
O	-3.57799500	0.43812900	-1.00925100
O	-2.40537100	0.16085300	-2.91671300
C	-3.15695200	1.22824700	-3.51341900
H	-2.90169300	1.21062100	-4.57288900
H	-2.86165200	2.18158700	-3.06914100
H	-4.22933700	1.07505500	-3.37151000
C	-1.53912400	2.53468600	0.07662000
O	-1.38045800	2.16672200	1.28240600
O	-0.94725800	2.06232200	-0.93940000
C	-2.58009700	3.60320800	-0.17103300
H	-3.50180400	3.09259500	-0.46920600
H	-2.26547900	4.25813200	-0.98576800
H	-2.76733500	4.17749700	0.73667700
Pd	-0.11728400	0.60762900	1.76533700
C	0.83870500	-1.85025800	2.88385900
O	1.28423400	-0.77563400	2.36546600
C	1.84415100	-2.83078600	3.44127000
H	1.63945000	-2.98442700	4.50451000
H	2.86213300	-2.46635800	3.30793200
H	1.71878200	-3.79500800	2.94093200
O	-0.39067100	-2.13078200	2.95150200
C	-3.71150400	-3.38228200	1.53033300
H	-3.61126700	-2.89919100	2.50784000
H	-2.97361400	-4.19081300	1.48767500
H	-4.70788700	-3.83516300	1.49227300
C	-2.00428300	-0.68503400	1.46656100
H	-1.01855300	-1.15041800	2.26034800
H	-2.44635200	0.26201500	1.17460400
H	-2.62008100	-0.99279900	2.32316700

8

Thermal correction to Gibbs Free Energy= 0.377504 Hartree

SCF Done: E(RM06) = -1781.71793713 Hartree

0 1

C	1.67970000	2.19139400	1.29782700
C	2.63495000	3.18649000	1.48041000
C	3.92360900	2.81954800	1.86066600
C	4.22115800	1.46645900	2.04265500
C	3.22094700	0.52105500	1.83617100
H	2.32861900	4.21353100	1.31695600
H	4.68811600	3.57480100	2.01593600
H	5.21139700	1.14063700	2.34165900
H	3.37992500	-0.54361000	1.96480500
N	1.98348500	0.88955300	1.46743400
C	0.27075500	2.50548400	0.87191000
O	-0.03475100	3.66594300	0.56979200
N	-0.53057400	1.41500400	0.84781800
Pd	0.37655300	-0.35234000	1.23046700
C	1.93116900	-2.66074900	0.28851000
O	1.65951900	-2.27363800	-0.89190900
O	1.54475000	-2.10620000	1.36578900
C	2.77881900	-3.91045100	0.43329600
H	2.19518700	-4.76707200	0.08101400
H	3.07290700	-4.07541400	1.46999200
H	3.66155000	-3.83644800	-0.20780600
C	-1.86452000	1.61677900	0.28167000
C	-1.91556400	1.50221700	-1.27500000
H	-2.10059700	2.66789500	0.50064900
C	-3.15510000	2.28499500	-1.80444400
H	-1.02918400	2.05311600	-1.61322900
H	-4.06449600	1.73567500	-1.53841700
H	-3.19775500	3.24336200	-1.27010100
C	-2.93016800	0.83003100	1.04146700
O	-3.82872600	0.18021900	0.54182100
O	-2.80872700	1.02673000	2.36722000
C	-3.74438600	0.31841500	3.19216600
H	-3.58828600	0.69425600	4.20344000
H	-3.53589600	-0.75326000	3.15099900
H	-4.77033700	0.50354600	2.86505400
C	-1.83373800	-2.31295800	0.60213300
O	-1.50212100	-2.41158200	-0.61349300
O	-1.34346600	-1.51033600	1.45779400
C	-2.96224700	-3.20050300	1.08323200
H	-3.88165400	-2.60659700	1.04308600
H	-2.79450700	-3.51099800	2.11625700
H	-3.07287100	-4.06787300	0.43162100
Pd	-0.24456600	-1.01759800	-1.50573500
C	2.14609900	0.32957800	-2.85597600
O	0.92357600	0.36754500	-2.65875700
C	2.82404800	1.35736200	-3.72037200
H	3.68495400	1.77792300	-3.19412900
H	2.11951900	2.14326600	-3.98828800
H	3.19971500	0.87239100	-4.62701300
O	2.95955000	-0.57831300	-2.36731900
C	-3.13712400	2.57619400	-3.30922500

H	-3.21739000	1.66564700	-3.91051600
H	-2.21428700	3.09065600	-3.60209700
H	-3.97748300	3.22257300	-3.58413100
C	-1.88616600	0.10475600	-1.89731400
H	2.47469000	-1.25905800	-1.80298000
H	-2.73284900	-0.50620800	-1.58430000
H	-1.85103400	0.17298900	-2.98911400

3-γ

Thermal correction to Gibbs Free Energy= 0.496052 Hartree

SCF Done: E(RM06) = -2091.85722476 Hartree

0 1

C	-3.01597900	-1.30761000	0.63032500
C	-3.83407700	-2.36508300	1.01403300
C	-4.33132200	-3.21899900	0.03263700
C	-3.98855600	-2.99656600	-1.30322700
C	-3.15500100	-1.92727400	-1.61780300
H	-4.05343800	-2.47516000	2.06976300
H	-4.98038000	-4.04700200	0.30145600
H	-4.35960500	-3.63665800	-2.09626900
H	-2.85224500	-1.68761200	-2.63080800
N	-2.68525400	-1.11257300	-0.66056000
C	-2.44886300	-0.33213300	1.62682600
O	-2.59231300	-0.53678500	2.83920200
N	-1.80241400	0.70406100	1.04330000
Pd	-1.62967700	0.60803900	-0.97838100
C	-0.22530400	-0.42105700	-3.31110300
O	0.68998400	-0.76995000	-2.52988400
O	-1.32215100	0.17926500	-2.99815400
C	-0.05184400	-0.70394500	-4.79581000
H	-1.01327200	-0.74001000	-5.31076200
H	0.49989800	-1.63527900	-4.93791500
H	0.54211900	0.10620200	-5.23286700
C	-1.09788900	1.60412200	1.95445200
C	0.33053800	1.10862000	2.32493500
H	-1.67815300	1.56739500	2.88514200
C	-1.19379100	3.06016200	1.50482400
O	-0.27306900	3.85413300	1.45616700
O	-2.47208800	3.39372400	1.25174600
C	-2.68791500	4.73378900	0.78689700
H	-3.76976800	4.86366400	0.75619500
H	-2.26422800	4.84964800	-0.21333100
H	-2.23064800	5.45930900	1.46398600
C	0.21245000	2.87014000	-1.57849100
O	1.22118700	2.12567300	-1.49879100
O	-0.98716300	2.53888200	-1.29065400
C	0.41281800	4.31310800	-1.99460600
H	0.43446800	4.91706400	-1.08111300
H	-0.41839400	4.65490500	-2.61455500
H	1.36006300	4.43111400	-2.52175000
Pd	1.17862400	0.20013100	-0.47621100
C	1.43267400	1.24431000	1.26998100
H	1.54304700	2.26654900	0.90876400
C	2.76085200	-1.13026800	0.15654100
C	1.69372000	-1.74491000	0.38769700
C	0.79059200	-2.82328500	0.70544000
C	0.57916000	-3.20605900	2.04249500
C	0.14968100	-3.53401900	-0.32752300
C	-0.24565200	-4.28991100	2.33786400
H	1.06643400	-2.65304600	2.83890100
C	-0.66466700	-4.62131500	-0.02098600
H	0.30742500	-3.21921600	-1.35374100
C	-0.86465800	-5.00284700	1.30923600
H	-0.40307000	-4.57738300	3.37310600
H	-1.14435100	-5.17474900	-0.82317200
H	-1.50010700	-5.85233800	1.54262300
C	4.17072900	-0.84147300	0.10032900
C	4.67625200	0.21168300	-0.68329300
C	5.06540400	-1.64006100	0.84130800
C	6.04745400	0.45377300	-0.72692700
H	3.98423600	0.83048300	-1.24573700
C	6.43380400	-1.39112100	0.78833100
H	4.67643500	-2.45046000	1.44980500
C	6.92894000	-0.34445600	0.00559800
H	6.42787600	1.26968200	-1.33424600
H	7.11522800	-2.01302500	1.36154700
H	7.99704700	-0.15101200	-0.03036700
H	2.38003600	0.90012500	1.69116300
H	0.19219600	0.04488800	2.55785400
C	0.85224700	1.77909700	3.63248300
H	1.83760500	1.34690600	3.84678100
H	1.01570600	2.84551500	3.44636300
C	-0.04166900	1.58742400	4.86629000
H	-0.33446200	0.53945300	4.99167700
H	0.48947200	1.90227100	5.77096500
H	-0.96090100	2.17897300	4.81304300

TS3-γ

Thermal correction to Gibbs Free Energy= 0.499426 Hartree

SCF Done: E(RM06) = -2091.82589574 Hartree

0 1

C	-1.76014300	1.54656100	-2.16045500
C	-1.88344400	2.66409800	-2.97943600
C	-2.66474000	3.73197500	-2.54509300
C	-3.29536600	3.65309800	-1.30129400
C	-3.12326900	2.50939400	-0.52723400
H	-1.36074700	2.65300000	-3.92898200
H	-2.78315300	4.61545000	-3.16507800
H	-3.91273400	4.46287700	-0.92823400
H	-3.58172900	2.37756300	0.44608900
N	-2.36777400	1.48805900	-0.96010600
C	-0.93185700	0.35479400	-2.55468500
O	-0.24796000	0.39276900	-3.58720900
N	-1.02440300	-0.67125300	-1.67775100
Pd	-2.09428400	-0.28292600	0.01666800
C	-2.20751000	0.89218200	2.67131000
O	-0.95089700	0.95054200	2.63225600
O	-2.98743200	0.42992700	1.76537800
C	-2.89282400	1.40532500	3.92682400
H	-2.34539700	2.25772000	4.33381300
H	-2.87493300	0.60798600	4.67763600
H	-3.93260100	1.67064100	3.72986300
C	-0.16018500	-1.81192700	-1.96801600
C	1.32642000	-1.62393400	-1.53748300
H	-0.12693100	-1.87898000	-3.06453700
C	-0.79610600	-3.12616800	-1.51623500
O	-0.26063700	-3.97523600	-0.82825000
O	-2.01858800	-3.26711400	-2.05364500
C	-2.74682300	-4.44032600	-1.65941500
H	-3.64203600	-4.45249800	-2.28110100
H	-3.01698400	-4.36399400	-0.60415700
H	-2.15016300	-5.34077600	-1.82340600
C	-1.44110800	-2.60575000	1.81883900
O	-0.46654100	-2.01932500	2.35275800
O	-2.12744500	-2.18798000	0.82078000
C	-1.83515300	-3.97297200	2.34374000
H	-1.42605100	-4.13033400	3.34226100
H	-1.41784300	-4.72234500	1.66276500
H	-2.92161500	-4.08454100	2.35122200
Pd	0.43944800	-0.25034200	1.38754100
C	2.40579500	0.20193300	0.42608900
C	1.54599400	1.16968900	0.52160300
C	1.31924900	2.57781900	0.29141900
C	1.73396100	3.16888000	-0.92069600
C	0.69266900	3.38258700	1.26480700
C	1.55461600	4.53309200	-1.13708700
H	2.17102800	2.54510200	-1.69373700
C	0.52597600	4.74672200	1.04108500
H	0.34358500	2.92019800	2.18121500
C	0.95774200	5.32767700	-0.15533300
H	1.88008400	4.97537800	-2.07431000
H	0.05671600	5.36129700	1.80419100
H	0.82442100	6.39255800	-0.32351200
C	3.85820900	0.03791400	0.46173600
C	4.46974500	-0.84737100	1.36792300
C	4.67329000	0.81063900	-0.38483300
C	5.85801400	-0.95341600	1.42454200
H	3.85037000	-1.43544500	2.03902000
C	6.06152400	0.70102300	-0.32066400
H	4.21475700	1.49541300	-1.08969500
C	6.65984900	-0.18241400	0.57964300
H	6.31370500	-1.63694300	2.13506000
H	6.67626400	1.30404000	-0.98279100
H	7.74138200	-0.26950700	0.62303900
C	1.60950000	-1.63491400	-0.02727800
H	2.60037200	-2.01586700	0.19485900
H	0.92297600	-2.29460200	0.50604300
C	2.20901800	-2.69870100	-2.23712600
H	2.09274900	-3.65392800	-1.71541800
H	1.81130200	-2.85139800	-3.24863600
C	3.69203300	-2.33390600	-2.35669600
H	4.23286500	-3.11558600	-2.90075300
H	3.82655200	-1.39418700	-2.90372000
H	4.17828500	-2.22009600	-1.38322800
H	1.60873900	-0.65580300	-1.96393600

4-γ

Thermal correction to Gibbs Free Energy= 0.502076 Hartree

SCF Done: E(RM06) = -2091.86330786 Hartree

0 1

C	0.34206200	-0.18791000	2.92321400
C	1.22754100	-0.27450500	3.99307300
C	1.61706400	-1.53405500	4.44231100
C	1.11114800	-2.66944000	3.80569000

C	0.23234900	-2.51164000	2.73807600
H	1.57652700	0.65217400	4.43413300
H	2.30398000	-1.63273100	5.27733900
H	1.38845900	-3.66787300	4.12522500
H	-0.20447000	-3.34715700	2.20462000
N	-0.13183300	-1.29093800	2.31483900
C	-0.11118500	1.13472700	2.37342100
O	0.38658300	2.18147100	2.81819100
N	-1.02654000	1.02962300	1.38508400
Pd	-1.49454700	-0.90149400	0.84295900
C	-0.90583400	-3.43125500	-0.62167600
O	-0.13856000	-2.82286900	-1.41929700
O	-1.60829200	-2.93462600	0.32313400
C	-1.01912800	-4.93415200	-0.81414600
H	-1.38883500	-5.41954000	0.09003100
H	-0.05314000	-5.34726700	-1.11042700
H	-1.72829900	-5.12315400	-1.62700900
C	-1.38100100	2.30766300	0.75084100
C	-0.27315000	2.88025600	-0.18326300
H	-1.47161500	3.02715500	1.57293900
C	-2.77167400	2.24621700	0.12249400
O	-3.02419400	2.32998300	-1.06363400
O	-3.70573900	2.16485700	1.08618200
C	-5.06902600	2.06164800	0.64783500
H	-5.67749100	2.21000200	1.54009200
H	-5.24228900	1.06851700	0.22986700
H	-5.29205000	2.82328600	-0.10293700
C	-3.28234400	-0.74707400	-1.57899500
O	-2.35149500	-0.99535500	-2.38236200
O	-3.16534200	-0.53363900	-0.31870700
C	-4.69135800	-0.63532400	-2.12763500
H	-4.89718500	0.42597700	-2.30163200
H	-5.42086000	-1.01679900	-1.41016300
H	-4.77006900	-1.16922200	-3.07543300
Pd	-0.27835100	-0.71583000	-1.73250100
C	0.21635100	1.93479900	-1.32055800
H	-0.64468200	1.27856700	-1.60562600
C	1.52261400	1.16536100	-1.05033300
C	1.53437400	-0.17414800	-1.16992000
C	2.61087600	-1.14428000	-0.94863400
C	3.33821100	-1.16272100	0.25745600
C	2.92747100	-2.09842200	-1.93464400
C	4.36066500	-2.08849500	0.45817900
H	3.10151500	-0.43796000	1.02905000
C	3.95134600	-3.02040400	-1.73209500
H	2.36144700	-2.10678700	-2.86078200
C	4.67259100	-3.01939400	-0.53528900
H	4.91732100	-2.08093900	1.39140900
H	4.18685400	-3.74226200	-2.50905800
H	5.47088000	-3.73930000	-0.37804400
C	2.75941500	1.98742200	-0.83603200
C	3.58074700	2.29115400	-1.93382500
C	3.11745200	2.48481700	0.42855200
C	4.72934600	3.06708500	-1.77581600
H	3.31528600	1.90814400	-2.91543000
C	4.26606000	3.26390600	0.58369500
H	2.49704600	2.26757200	1.29356500
C	5.07499300	3.55701600	-0.51513600
H	5.35416800	3.28677700	-2.63711000
H	4.52714100	3.64128700	1.56855100
H	5.96867300	4.16195900	-0.39029300
H	0.37549800	2.52971300	-2.22834800
H	0.57024600	3.04226200	0.49228400
C	-0.63790900	4.26843000	-0.76347500
H	0.25055000	4.63186400	-1.29527700
H	-1.42891900	4.15666500	-1.51176900
C	-1.04590200	5.32031400	0.27620300
H	-0.30412200	5.39864700	1.07901000
H	-1.13524700	6.30540500	-0.19333600
H	-2.01307500	5.09695900	0.73855400

TS5

Thermal correction to Gibbs Free Energy= 0.376646 Hartree

SCF Done: E(RM06) = -1781.68711396 Hartree

0 1

C	-1.31980100	2.70965400	-0.55749900
C	-2.25149900	3.74155700	-0.52094300
C	-3.52485400	3.51483200	-1.03604000
C	-3.82703700	2.26370200	-1.57928000
C	-2.84634500	1.27679800	-1.59041700
H	-1.93973900	4.68451300	-0.08668300
H	-4.27390600	4.30060800	-1.01856700
H	-4.80581500	2.04836800	-1.99356600
H	-3.00573800	0.29135500	-2.01068600
N	-1.62625400	1.50969800	-1.08219800
C	0.06843000	2.87304100	-0.00594200
O	0.41472300	3.96100300	0.46481400
N	0.80672200	1.73741300	-0.07851900

Pd	-0.06775500	0.20377900	-1.07935700
C	-1.61146100	-2.38525300	-1.75540100
O	-1.49655700	-2.90926000	-0.60339600
O	-1.18185000	-1.26001900	-2.14846600
C	-2.39665500	-3.20842500	-2.76302700
H	-2.07752700	-2.96608400	-3.77751900
H	-3.45953900	-2.96349500	-2.66194300
H	-2.27456300	-4.27324700	-2.56137200
C	2.12232200	1.77361700	0.56059300
C	2.17848100	1.26333200	2.03170600
H	2.38182100	2.84144900	0.60913500
C	3.21544600	1.17510600	-0.32535300
O	4.08930400	0.41654300	0.04971100
O	3.15347400	1.67882100	-1.57129200
C	4.13869000	1.19099700	-2.49290800
H	4.02508500	1.79558600	-3.39269200
H	3.94821200	0.13890800	-2.71797100
H	5.14440500	1.29832300	-2.07969000
C	1.95450500	-2.01776500	-0.76116500
O	1.27176300	-2.60504300	0.13684700
O	1.65623200	-0.92709100	-1.33062100
C	3.26409600	-2.65890700	-1.15480100
H	4.06981300	-2.00595400	-0.80416500
H	3.33002400	-2.72213800	-2.24405400
H	3.36232200	-3.64908500	-0.71040600
Pd	-0.47866900	-1.89957900	0.91031200
C	-2.39487200	-0.34927300	2.26493600
O	-2.22987700	-1.56357400	1.79600700
C	-3.71535900	-0.16210000	2.99081900
H	-3.80828800	0.87295500	3.32167600
H	-3.76151400	-0.83150200	3.85510400
H	-4.54903200	-0.42400900	2.33287800
O	-1.56573300	0.55091500	2.13773900
C	1.81989500	-0.22403800	2.22749500
H	0.74667200	-0.35157400	1.94559100
H	2.42718100	-0.82509600	1.55174100
C	2.01412600	-0.74012700	3.65823100
H	1.86749800	-1.82473600	3.70173800
H	3.03091400	-0.53089600	4.01035000
H	1.31347500	-0.28126800	4.36094500
C	1.37024200	2.18370900	2.96178700
H	1.60051300	1.96794200	4.00933300
H	1.60109400	3.23546100	2.77541100
H	0.29581000	2.04214100	2.81721900
H	3.24114500	1.34742500	2.30179600

9

Thermal correction to Gibbs Free Energy= 0.374375 Hartree

SCF Done: E(RM06) = -1781.68874036 Hartree

0 1

C	-1.59016200	2.65638500	-0.32970300
C	-2.60707100	3.59760900	-0.21621500
C	-3.84541400	3.31997700	-0.78890400
C	-4.02801400	2.10981600	-1.46152300
C	-2.96595500	1.21422400	-1.54190100
H	-2.38853500	4.51243800	0.32251000
H	-4.65939000	4.03460900	-0.71420800
H	-4.97668900	1.85592100	-1.92154500
H	-3.03337600	0.26506100	-2.05953700
N	-1.78124000	1.49580200	-0.98143100
C	-0.23840500	2.86875400	0.29077000
O	-0.00230300	3.91029200	0.91146100
N	0.59977200	1.81704000	0.10837000
Pd	-0.11372900	0.33530600	-1.07307000
C	-1.43835900	-2.31613300	-1.90959000
O	-1.24316300	-2.90722200	-0.79991600
O	-1.08993700	-1.14644100	-2.24736900
C	-2.22146000	-3.12226600	-2.93272100
H	-2.04651500	-2.73306200	-3.93623600
H	-3.28889200	-3.04194500	-2.70065700
H	-1.94568000	-4.17625400	-2.87279700
C	1.87182000	1.86257200	0.82529200
C	1.90081600	1.14287000	2.20792200
H	2.02725300	2.92766500	1.05202900
C	3.05881100	1.50184800	-0.06832300
O	3.96760300	0.75156400	0.23570600
O	3.02501000	2.20430200	-1.21338900
C	4.09217000	1.95143100	-2.13817200
H	3.98843700	2.70361000	-2.92010100
H	3.98628400	0.94987100	-2.56181400
H	5.06310300	2.03776900	-1.64477900
C	2.15748900	-1.66720900	-1.11997100
O	1.57677300	-2.45655000	-0.30949800
O	1.73257400	-0.53373700	-1.48552600
C	3.50127000	-2.10402300	-1.65484700
H	4.26352200	-1.48171000	-1.17458300
H	3.54703100	-1.92680600	-2.73207400
H	3.68671400	-3.15470800	-1.43236000

Pd	-0.13716200	-2.01632700	0.71388000
C	-2.33199500	-0.87149800	2.18564700
O	-1.73796900	-2.00593000	1.89553300
C	-3.51722000	-1.06574600	3.12114800
H	-3.93195900	-0.09416300	3.39196500
H	-3.20586900	-1.60415600	4.02062600
H	-4.28286300	-1.67241400	2.62804300
O	-1.99437200	0.23121600	1.76348500
C	1.76127800	-0.39517400	2.17962000
H	0.69815900	-0.60743000	1.86266300
H	2.44103500	-0.80373600	1.43419300
C	2.01490600	-1.08272100	3.52572400
H	2.01089300	-2.17152600	3.41327100
H	2.99649200	-0.79326600	3.91764100
H	1.26251400	-0.82185200	4.27434000
C	0.90257100	1.79272200	3.17981000
H	1.07767300	1.44867700	4.20335900
H	0.99251900	2.88142400	3.16838200
H	-0.12649500	1.53925000	2.90670600
H	2.91969700	1.32319900	2.58084800

TS2-γ′

Thermal correction to Gibbs Free Energy= 0.374807 Hartree

SCF Done: E(RM06) = -1781.66449916 Hartree

0 1

C	2.32343000	-1.87973700	-0.74671800
C	3.56594300	-2.50514000	-0.76054400
C	4.65626900	-1.82136000	-1.29355700
C	4.47258700	-0.53124100	-1.79765300
C	3.20264400	0.03688800	-1.75590400
H	3.62912800	-3.50714200	-0.35153000
H	5.63767800	-2.28521900	-1.31892100
H	5.29638000	0.03246200	-2.22157900
H	2.98101800	1.02701100	-2.13768900
N	2.16338900	-0.63439700	-1.23484800
C	1.10346200	-2.54160100	-0.16192600
O	1.19792700	-3.66940600	0.33404300
N	0.00057200	-1.75711900	-0.22406500
Pd	0.22773600	-0.00091800	-1.22320600
C	0.58827300	2.96981700	-1.40501600
O	0.18044600	3.13805800	-0.22093000
O	0.72192300	1.87202800	-2.04449900
C	0.93790000	4.23156500	-2.17630700
H	0.01981500	4.63204200	-2.61948800
H	1.64009600	4.01169000	-2.98178600
H	1.34404600	4.98626400	-1.50099000
C	-1.20300900	-2.25187700	0.44279100
C	-1.43237800	-1.78805600	1.91500000
H	-1.07083400	-3.34246700	0.49920600
C	-2.44567600	-2.08700100	-0.43443200
O	-3.51723700	-1.64630100	-0.06315400
O	-2.23164000	-2.59323400	-1.66045900
C	-3.32581400	-2.49697900	-2.58417600
H	-3.03801600	-3.10049300	-3.44499300
H	-3.46484300	-1.45554900	-2.88293100
H	-4.24685300	-2.87683200	-2.13600800
C	-2.56851600	1.15534500	-0.94593800
O	-2.30187300	1.72966000	0.15355200
O	-1.78897400	0.40622500	-1.60680500
C	-3.96795800	1.34440100	-1.48704900
H	-4.55730100	0.47862000	-1.16831200
H	-3.95188500	1.37369200	-2.57825400
H	-4.41685700	2.25229900	-1.08322200
Pd	-0.45160300	1.56681000	1.05983800
C	1.79938200	0.77226600	2.63323600
O	1.36865800	1.78406200	2.00729300
C	3.15030200	0.85918100	3.30152400
H	3.00871500	0.84067100	4.38650900
H	3.66590300	1.77555600	3.01620200
H	3.74678300	-0.01659900	3.03461400
O	1.14497200	-0.31045700	2.73953300
C	-1.43787900	-0.24028700	2.19141000
H	-0.02384900	-0.11038600	2.24370300
H	-2.24922400	0.12843000	1.57064500
C	-1.78278800	0.11737900	3.64599900
H	-2.03310300	1.17947500	3.72842600
H	-2.65619700	-0.45487700	3.98776000
H	-0.96094000	-0.08737600	4.33790300
C	-0.58710200	-2.64146800	2.87823500
H	-0.77408300	-2.35936900	3.91811600
H	-0.86376700	-3.69508000	2.76786100
H	0.48265200	-2.56233000	2.69144100
H	-2.48235600	-2.05259000	2.10818200

10

Thermal correction to Gibbs Free Energy= 0.378496 Hartree

SCF Done: E(RM06) = -1781.71782392 Hartree

0 1

C	1.52751600	-2.70761700	0.58604300
C	2.49018000	-3.62209400	1.00180500
C	3.67374300	-3.72978600	0.27557900
C	3.85959000	-2.91993900	-0.84765900
C	2.85573900	-2.02661800	-1.21002600
H	2.27162600	-4.21631500	1.88180100
H	4.44172500	-4.43625800	0.57567200
H	4.76473500	-2.97713700	-1.44235100
H	2.92533300	-1.38298900	-2.07948100
N	1.72355200	-1.92702700	-0.49533400
C	0.23619000	-2.51555000	1.33756700
O	0.03355500	-3.15562000	2.37629100
N	-0.56521700	-1.58243400	0.77132400
Pd	0.07992900	-0.80482100	-0.97744500
C	1.49502200	1.26696200	-2.52349100
O	1.40882400	2.00508600	-1.49368000
O	1.01823100	0.09408800	-2.64223700
C	2.21360600	1.83502600	-3.73353500
H	1.62473000	2.66898700	-4.12847800
H	2.34155000	1.08213800	-4.51146600
H	3.18411700	2.23987900	-3.43263900
C	-1.75325100	-1.17696000	1.51706000
C	-1.60530800	0.12198400	2.37246100
H	-1.90619300	-1.98106000	2.25232700
C	-3.01888000	-1.22950300	0.66097700
O	-3.91084300	-0.40288300	0.66290200
O	-3.07570600	-2.38055800	-0.03286200
C	-4.21845500	-2.54110900	-0.88448000
H	-4.17126500	-3.56696700	-1.25005000
H	-4.15865300	-1.83979200	-1.72016700
H	-5.14458800	-2.37020500	-0.33051300
C	-2.22902500	1.00754700	-1.73102500
O	-1.73949800	2.00807600	-1.13600800
O	-1.77961900	-0.18094400	-1.70219500
C	-3.50837900	1.22419800	-2.51299100
H	-4.34146900	0.94201300	-1.86019700
H	-3.53097400	0.58730500	-3.39924000
H	-3.61666500	2.27378600	-2.78847500
Pd	-0.18330400	1.80629600	0.24700100
C	2.58457000	2.29609100	1.53039000
O	1.39952200	1.98882700	1.71859000
C	3.52654000	2.49636500	2.68739800
H	4.43328400	1.90454500	2.53660800
H	3.03861600	2.21549600	3.61955700
H	3.82648800	3.54827700	2.72899000
O	3.13809000	2.48780700	0.35529600
C	-1.63471300	1.49083800	1.66124800
H	2.48405600	2.34318700	-0.40087900
H	-2.53128200	1.54098500	1.04422200
C	-1.59927700	2.68349100	2.61110300
H	-1.80595600	3.61302000	2.07197900
H	-2.37457600	2.57537800	3.38586500
H	-0.63750900	2.78958700	3.11960500
C	-0.45720900	-0.05481000	3.38112600
H	-0.46661000	0.74553000	4.12556800
H	-0.54545600	-1.01049000	3.90416900
H	0.51467400	-0.03150900	2.88375600
H	-2.54248600	0.13886400	2.95624700

3-γ′

Thermal correction to Gibbs Free Energy= 0.496540 Hartree

SCF Done: E(RM06) = -2091.85479337 Hartree

0 1

C	3.19893500	-1.47691500	-0.35104900
C	4.02278400	-2.58583900	-0.51500000
C	4.31727800	-3.37385000	0.59498000
C	3.78053400	-3.02901900	1.83810000
C	2.96161100	-1.90728900	1.93274400
H	4.40491300	-2.78609600	-1.50942800
H	4.96084800	-4.24299200	0.49751800
H	3.99365700	-3.61203000	2.72750200
H	2.52603000	-1.56805900	2.86562200
N	2.67880600	-1.16374200	0.85174000
C	2.83188100	-0.58027900	-1.50545000
O	3.22639300	-0.85770600	-2.64410600
N	2.04687300	0.45814000	-1.13247500
Pd	1.68282100	0.62343800	0.85592900
C	0.04464200	0.01626700	3.17748300
O	-0.82356700	-0.38907600	2.37205300
O	1.20570300	0.49406000	2.88155900
C	-0.26788300	-0.03043700	4.66567600
H	-0.87952600	-0.90620100	4.89179100
H	-0.85228300	0.85993000	4.92237300
H	0.64317300	-0.02812400	5.26630800
C	1.49699000	1.29115600	-2.19369900
C	0.04715800	0.93525500	-2.63987100
H	2.12508300	1.08761600	-3.07395800
C	1.70891100	2.78282400	-1.92765400

O	0.88581200	3.65758400	-2.11868000
O	2.97456400	3.02485300	-1.54461100
C	3.28438000	4.39389100	-1.24791100
H	4.36082000	4.42178900	-1.07874100
H	2.75135700	4.70623300	-0.34682800
H	3.00680700	5.04510700	-2.08022200
C	-0.01487500	3.06826700	1.06394500
O	-1.07538500	2.39912500	1.00353900
O	1.16476300	2.61641600	0.86979800
C	-0.10737300	4.55886100	1.32323400
H	-1.07952500	4.81464800	1.74551800
H	0.01120900	5.07028800	0.36180400
H	0.69769900	4.88465300	1.98516300
Pd	-1.11908300	0.36001300	0.15834400
C	-1.12071700	1.30684800	-1.70425600
H	-0.95891000	2.31371000	-1.32311200
C	-2.47486000	1.27037900	-2.40046900
H	-2.44220000	1.93270700	-3.27834000
H	-2.76018800	0.27529500	-2.74960000
H	-3.26703300	1.64053500	-1.74592800
C	0.00130700	-0.49766300	-3.19077400
H	-0.97434900	-0.71620800	-3.63362200
H	0.76496900	-0.64288200	-3.95938300
H	0.18445100	-1.23212700	-2.40447700
H	-0.12102800	1.61290400	-3.49442500
C	-2.86473300	-0.88052100	-0.18406100
C	-1.87795200	-1.62893700	-0.37979000
C	-1.15946100	-2.86788600	-0.55456100
C	-1.39089200	-3.66821000	-1.68908400
C	-0.25873800	-3.31467500	0.43024500
C	-0.73663900	-4.89003400	-1.83104000
H	-2.08060900	-3.32246500	-2.45226900
C	0.38498900	-4.54075400	0.28146700
H	-0.09660200	-2.69426700	1.30494300
C	0.15204300	-5.33048700	-0.84791200
H	-0.92152400	-5.49877100	-2.71128700
H	1.07113800	-4.88218100	1.05112900
H	0.65907800	-6.28434100	-0.96086800
C	-4.22992400	-0.45272900	-0.03263400
C	-4.56912700	0.65917500	0.76122900
C	-5.25337300	-1.17459500	-0.67914900
C	-5.90323500	1.03101800	0.90796200
H	-3.77847700	1.22108900	1.24821800
C	-6.58398700	-0.79623100	-0.52260300
H	-4.99347500	-2.02910300	-1.29598400
C	-6.91324800	0.30678600	0.26952600
H	-6.15478900	1.89051000	1.52227100
H	-7.36527200	-1.36093500	-1.02300500
H	-7.95195400	0.60196800	0.38576100

TS3-γ′

Thermal correction to Gibbs Free Energy= 0.499244s Hartree

SCF Done: E(RM06) = -2091.81277973 Hartree

0 1

C	-2.49873500	1.72886500	-1.75580800
C	-2.92302400	2.86512900	-2.43685000
C	-3.47548500	3.91730800	-1.71052800
C	-3.59056300	3.79985700	-0.32365000
C	-3.14740200	2.63480300	0.29567500
H	-2.80371400	2.87959600	-3.51414900
H	-3.81549400	4.81636900	-2.21550800
H	-4.01947000	4.59422300	0.27732600
H	-3.22306800	2.46786500	1.36363800
N	-2.60797800	1.63290100	-0.41604200
C	-1.87502900	0.56080400	-2.47187600
O	-1.71081100	0.61056400	-3.69745400
N	-1.52254300	-0.44176600	-1.63268900
Pd	-2.04330300	-0.18818600	0.31984900
C	-1.52164300	0.76459000	3.02994000
O	-0.31185600	0.87464000	2.70442800
O	-2.48173500	0.34882700	2.28927300
C	-1.90658900	1.14889100	4.44899500
H	-1.31702400	2.00671100	4.77914900
H	-1.66637800	0.30774000	5.10815500
H	-2.97420200	1.35880200	4.52877100
C	-0.78610900	-1.55200400	-2.21703900
C	0.76838900	-1.47982000	-2.09039600
H	-0.98687500	-1.50065800	-3.29772400
C	-1.34871000	-2.90992400	-1.79249400
O	-0.68060400	-3.87072400	-1.45800700
O	-2.68298200	-2.94116300	-1.92727200
C	-3.32400500	-4.15790500	-1.51529100
H	-4.37070400	-4.04855800	-1.79876300
H	-3.23275200	-4.27004500	-0.43275500
H	-2.87637900	-5.02116200	-2.01339600
C	-1.10815100	-2.64373100	1.80674500
O	-0.06217600	-2.09687500	2.23320000
O	-1.90042400	-2.17125100	0.91509600

C	-1.47596300	-4.02268900	2.32308900
H	-2.53841700	-4.06208100	2.57561300
H	-0.86782600	-4.28165500	3.19015300
H	-1.29357600	-4.74760200	1.52269100
Pd	0.76683400	-0.32494600	1.15666300
C	1.33295200	-1.75677800	-0.67338700
H	0.52206400	-2.12732100	-0.05205600
C	2.40948400	-2.82685700	-0.63242900
H	1.92326500	-3.75339100	-0.95966000
H	3.25203100	-2.62231900	-1.29676200
H	2.79155000	-2.98734500	0.37731100
C	1.30298700	-0.25800700	-2.84766000
H	2.39380500	-0.28511000	-2.90031400
H	0.90845500	-0.24812400	-3.86687300
H	1.00537700	0.67606800	-2.36833400
H	1.10890600	-2.35872900	-2.65622800
C	2.56249400	-0.06495900	-0.10244400
C	1.89042800	1.00969200	0.20053300
C	1.88151700	2.45031500	0.10751600
C	2.54534300	3.09288300	-0.95991300
C	1.22867800	3.23851400	1.07763900
C	2.56886100	4.48202600	-1.04219300
H	3.03653400	2.49223100	-1.71762700
C	1.25913900	4.62761600	0.98603600
H	0.71295600	2.74191300	1.89241000
C	1.92706500	5.25416700	-0.07015900
H	3.08576400	4.96345600	-1.86736000
H	0.76147500	5.22616200	1.74393300
H	1.94578800	6.33823100	-0.13723500
C	3.98551700	-0.41317000	-0.17707300
C	4.61446400	-1.01749700	0.92738500
C	4.76455000	-0.09022300	-1.30140100
C	5.98004100	-1.29100200	0.90378000
H	4.02006200	-1.25817300	1.80371800
C	6.13343100	-0.36043300	-1.31737400
H	4.29923200	0.37805100	-2.16212900
C	6.74530100	-0.96543700	-0.21895500
H	6.44855700	-1.75417200	1.76741700
H	6.72054000	-0.09828800	-2.19296100
H	7.81000400	-1.17884700	-0.23548500

4-γ′

Thermal correction to Gibbs Free Energy= 0.501080 Hartree

SCF Done: E(RM06) = -2091.85060323 Hartree

0 1

C	-2.40554500	1.89646200	-1.46964800
C	-2.87096400	3.11164800	-1.96339700
C	-3.56148600	3.96570700	-1.10763600
C	-3.77477900	3.57378200	0.21600800
C	-3.28437900	2.34391000	0.64452900
H	-2.67934500	3.33517300	-3.00658600
H	-3.93709700	4.91955800	-1.46570900
H	-4.31821700	4.20360200	0.91195600
H	-3.43326100	1.96811000	1.64934900
N	-2.60590100	1.53717500	-0.18735900
C	-1.68058200	0.91470300	-2.34982100
O	-1.51183100	1.18822200	-3.54621400
N	-1.28303800	-0.20217700	-1.69595700
Pd	-1.89120100	-0.32613300	0.26740200
C	-1.78699300	0.28439700	3.14425600
O	-0.61625600	0.70517400	2.98006100
O	-2.56181600	-0.19292900	2.23769600
C	-2.37414300	0.31959000	4.54578200
H	-2.01266400	1.19895200	5.08287600
H	-2.02146000	-0.56781300	5.08252500
H	-3.46485200	0.29924600	4.52347800
C	-0.62801200	-1.21732600	-2.51914800
C	0.92869700	-1.21469000	-2.49306800
H	-0.91156600	-0.98561500	-3.55676600
C	-1.20975100	-2.61286300	-2.27516900
O	-0.56475000	-3.64089200	-2.21214400
O	-2.55369600	-2.57700700	-2.24521200
C	-3.19953600	-3.83142600	-1.98228000
H	-4.26788300	-3.63803000	-2.07970900
H	-2.96187000	-4.15898900	-0.96795800
H	-2.87786100	-4.59201400	-2.69786700
C	-0.80820700	-2.83652700	1.53909100
O	-0.02182000	-2.22844500	2.29661600
O	-1.46707000	-2.33042500	0.55229400
C	-1.03128500	-4.32736600	1.72892400
H	-0.39333000	-4.71189100	2.52495800
H	-0.81451900	-4.84338400	0.78898900
H	-2.08174100	-4.51345200	1.97313700
Pd	0.82960200	-0.26259600	1.51961400
C	1.53212800	-1.55546200	-1.07992500
H	0.68455000	-1.89570600	-0.49092700
C	2.46485900	-2.77708700	-1.17397000
H	1.87791500	-3.61691700	-1.55171300

H	3.30803500	-2.61572000	-1.85230700
H	2.87155900	-3.04666100	-0.19468800
C	1.47067300	0.04410100	-3.17810300
H	2.56275400	0.01675900	-3.24830700
H	1.07370800	0.12763400	-4.19335800
H	1.18219900	0.95464100	-2.65106800
H	1.19958400	-2.06400500	-3.13086400
C	2.20431900	-0.41069500	-0.22790600
C	1.62769400	0.79724500	0.18250100
C	1.67318000	2.20988600	-0.05542800
C	2.47919200	2.77117300	-1.07499800
C	0.91190000	3.07036900	0.77230200
C	2.52179100	4.14790400	-1.25424000
H	3.07809600	2.11720100	-1.69718500
C	0.94180000	4.44452100	0.56763000
H	0.30893700	2.62822700	1.55998500
C	1.74818000	4.98393300	-0.44090800
H	3.15131300	4.57394900	-2.02928900
H	0.35101000	5.09996700	1.20012500
H	1.77912500	6.05951900	-0.59070100
C	3.70477900	-0.40559700	-0.01484900
C	4.24060500	-0.36057300	1.28298300
C	4.60140400	-0.39623600	-1.09952900
C	5.61970700	-0.31147000	1.49250600
H	3.55900400	-0.37883800	2.12846500
C	5.97882900	-0.34941500	-0.89027600
H	4.22145600	-0.42617700	-2.11456500
C	6.49513700	-0.30576500	0.40697300
H	6.00635400	-0.28363100	2.50726100
H	6.65009200	-0.34660100	-1.74458400
H	7.56874900	-0.27110000	0.56762500

TS2-β

Thermal correction to Gibbs Free Energy= 0.374244 Hartree

SCF Done: E(RM06) = -1781.63397189 Hartree

0 1

C	2.31525300	-1.47402500	-1.31404400
C	3.52171700	-2.13270800	-1.52678600
C	4.66856500	-1.37667100	-1.75918700
C	4.57920800	0.01811400	-1.76426500
C	3.34233900	0.61636000	-1.54169800
H	3.51675400	-3.21640100	-1.49523800
H	5.62294300	-1.86469900	-1.93247100
H	5.45078600	0.63951600	-1.93847000
H	3.19307100	1.69079100	-1.53407400
N	2.24644600	-0.12721700	-1.32299300
C	1.04197800	-2.21298000	-0.98658900
O	1.06432600	-3.43492600	-0.80580200
N	-0.01618300	-1.37916000	-0.86577600
Pd	0.34309500	0.58292600	-1.17749600
C	0.61038800	3.30066600	-0.02583200
O	0.02682000	2.91995700	1.03134000
O	0.90196100	2.61446400	-1.06316500
C	0.98323200	4.77347600	-0.08376800
H	0.12416800	5.33388300	-0.46810700
H	1.82441000	4.93119400	-0.76071000
H	1.21282000	5.14626600	0.91551500
C	-1.22988800	-1.88094100	-0.22099100
C	-1.31687800	-1.52703600	1.32862000
H	-1.13140000	-2.97640400	-0.27199300
C	-0.87002700	-2.83429500	2.09087400
H	0.08070900	-1.06488100	1.61394300
H	-1.64316400	-3.57688200	1.83923400
H	0.06356300	-3.21426500	1.67399000
C	-2.44883300	-1.62920500	-1.12100700
O	-3.57339800	-1.32584500	-0.77642900
O	-2.13061000	-1.90469500	-2.40083400
C	-3.19523800	-1.75345000	-3.35028400
H	-2.79347300	-2.10891800	-4.29901000
H	-3.48194700	-0.70187500	-3.43213300
H	-4.06764400	-2.34169300	-3.05521200
C	-2.48903600	1.49060800	-0.69609200
O	-2.34833300	1.41231900	0.56124900
O	-1.61872600	1.17176900	-1.55928900
C	-3.83642000	1.95392500	-1.19999900
H	-4.47861800	1.07002300	-1.26854800
H	-3.73986700	2.40360900	-2.18929100
H	-4.28440300	2.65714100	-0.49640300
Pd	-0.57933300	0.90124000	1.47687300
C	1.77291300	-0.27510500	2.51549200
O	1.12258100	0.80325300	2.63256900
C	3.11582200	-0.39289300	3.19229600
H	3.83093100	-0.86062000	2.51191500
H	3.01345500	-1.04799500	4.06331700
H	3.47361200	0.58430400	3.51531600
O	1.32820600	-1.27365100	1.86949000
C	-0.76312900	-2.74900900	3.61586100
H	-1.73407700	-2.59842200	4.09460400

H	-0.10417400	-1.93515900	3.93388900
H	-0.34503300	-3.68156200	4.00913900
C	-2.69828500	-1.17130900	1.91094000
H	-3.20403900	-0.35983600	1.40393700
H	-3.35488900	-2.05012400	1.85572400
H	-2.59408900	-0.90625500	2.96690500

11

Thermal correction to Gibbs Free Energy= 0.557657 Hartree

SCF Done: E(RM06) = -2320.92269008 Hartree

0 1

C	2.39600700	-3.04109000	-0.71213300
C	2.48751900	-4.39013700	-1.03976800
C	2.20736800	-5.34087200	-0.06177800
C	1.84344900	-4.91293900	1.21706000
C	1.76555300	-3.54795400	1.47676000
H	2.77598800	-4.64123900	-2.05411600
H	2.27340800	-6.40077200	-0.28820500
H	1.62243700	-5.62050500	2.00868200
H	1.50307300	-3.14906300	2.44899300
N	2.03018300	-2.64114500	0.52267000
C	2.67023900	-1.95963500	-1.72274000
O	2.89175900	-2.26555700	-2.90122800
N	2.61144000	-0.71962400	-1.18780000
Pd	2.06913000	-0.60668800	0.76049600
C	0.25489500	-0.65325400	3.11646600
O	-0.73153100	-0.30581500	2.40321600
O	1.47159200	-0.74856000	2.76206300
C	-0.06201700	-1.00742400	4.55896900
H	-0.18199100	-0.07503200	5.12100100
H	0.74314000	-1.58844700	5.01074400
H	-1.00974800	-1.54759600	4.60967000
C	2.67946300	0.40840000	-2.10493500
C	1.31308300	1.12288500	-2.34320400
H	3.01848000	-0.01359500	-3.06209200
C	0.12038600	0.15192500	-2.22362100
H	1.20483500	1.87506000	-1.55298400
H	0.16038300	-0.59210600	-3.03049100
H	0.21082900	-0.40072500	-1.28688900
C	3.76341500	1.40969200	-1.68765000
O	3.67504100	2.61835200	-1.77480400
O	4.87977900	0.78102100	-1.27285200
C	1.32622900	1.84965600	-3.69750800
H	1.40896200	1.13143900	-4.52287500
H	2.16467900	2.54672900	-3.75593000
H	0.40565500	2.42357200	-3.84857400
C	5.95002000	1.64206800	-0.86202300
H	6.78198300	0.98170700	-0.61673400
H	5.65306800	2.22201000	0.01558700
H	6.22768900	2.32936700	-1.66542700
C	1.97550000	2.30568200	1.58772600
O	0.79763500	2.30319700	2.02390100
O	2.54451700	1.38592700	0.90285400
C	2.85721700	3.51091300	1.86205000
H	2.28675100	4.30694800	2.34100900
H	3.28019900	3.85843200	0.91508000
H	3.68984900	3.21379700	2.50740200
Pd	-0.82240300	1.25043900	0.99702400
C	-2.31881300	0.30415800	0.00566000
C	-2.32705400	0.22114400	-1.35410800
C	-3.39093500	-0.51517500	-2.11176800
C	-3.03165600	-1.34730800	-3.18788400
C	-4.75819800	-0.37177900	-1.81944200
C	-3.99957400	-2.02817700	-3.92581800
H	-1.98351600	-1.47376100	-3.44122300
C	-5.72650700	-1.04293200	-2.56445500
H	-5.06712200	0.27299400	-1.00359600
C	-5.35277800	-1.87819700	-3.61885400
H	-3.69359800	-2.67370900	-4.74445900
H	-6.77691400	-0.90791200	-2.32190400
H	-6.10797700	-2.40151500	-4.19821000
C	-3.23382700	-0.41595100	0.92111700
C	-4.02495500	0.27740300	1.85500700
C	-3.29510800	-1.82174800	0.91358900
C	-4.86739400	-0.40634400	2.73028200
H	-3.97755800	1.36260800	1.89269600
C	-4.13194600	-2.50505900	1.79293300
H	-2.68322200	-2.37072400	0.20494400
C	-4.92439600	-1.80134500	2.70358000
H	-5.47727300	0.15096800	3.43597900
H	-4.16745100	-3.59088500	1.76685200
H	-5.57798900	-2.33537400	3.38747500
C	-1.24662500	0.88216500	-2.21603500
H	-1.62812200	0.99882800	-3.23449800
H	-1.07265700	1.89218900	-1.83761300
C	-2.16037800	3.75412400	-0.20013600
O	-1.10099700	3.19170100	0.10198900
O	-3.29295000	3.11420400	-0.41713500

H	-3.11648100	2.13505100	-0.37015300
C	-2.24956700	5.24363900	-0.36065900
H	-1.27926300	5.69674300	-0.16464200
H	-2.99727500	5.63794600	0.33410300
H	-2.58450700	5.48445700	-1.37370900

11’

Thermal correction to Gibbs Free Energy= 0.558632 Hartree

SCF Done: E(RM06) = -2320.93548226 Hartree

0 1

C	-3.42908000	0.16797000	2.15899200
C	-4.05994900	-0.03996700	3.38216200
C	-4.43999700	-1.33295700	3.73150600
C	-4.18877700	-2.37945900	2.84054500
C	-3.56084900	-2.10232200	1.63006700
H	-4.23800100	0.82690900	4.00813600
H	-4.93281200	-1.52526700	4.67980100
H	-4.47928200	-3.39899600	3.06957700
H	-3.35605700	-2.85822800	0.88204600
N	-3.18627100	-0.85165500	1.31410100
C	-3.03370900	1.54372800	1.69694200
O	-3.36329900	2.52345100	2.37857800
N	-2.34798600	1.54654600	0.52931900
Pd	-2.25261600	-0.28937300	-0.40615200
C	-1.70745900	-3.04258600	-1.56155800
O	-0.47699500	-2.99201700	-1.34531900
O	-2.58574800	-2.16912000	-1.23344500
C	-2.24679100	-4.25374700	-2.30758600
H	-3.29838100	-4.42574400	-2.07218400
H	-1.64728100	-5.13573200	-2.07552200
H	-2.16492900	-4.06253100	-3.38313800
C	-2.09990000	2.87387700	-0.04962300
C	-0.73318200	3.54316900	0.25984500
H	-2.85988600	3.53201500	0.39845300
C	0.52587200	2.71567400	-0.07828300
H	-0.70504700	4.39905800	-0.42507600
H	0.42781200	2.29183200	-1.08104300
H	1.36773400	3.41748800	-0.12905800
C	-2.42767000	2.91096700	-1.54586900
O	-1.75819800	3.45735200	-2.39982000
O	-3.63021900	2.35367900	-1.78900400
C	-0.69086200	4.10780900	1.68943300
H	-0.82388000	3.33464600	2.44978300
H	-1.48899600	4.83969300	1.84473500
H	0.26862400	4.60665200	1.86951800
C	-4.03221900	2.32035000	-3.16620000
H	-5.05386200	1.94022000	-3.16550300
H	-3.37646300	1.64835700	-3.72407100
H	-3.99217600	3.31969700	-3.60620400
C	-0.30895400	0.26731000	-2.73264400
O	0.70999000	-0.32884800	-2.27683700
O	-1.45139300	0.39046100	-2.19208600
C	-0.16132500	0.93209600	-4.08885200
H	0.86586500	0.86133300	-4.44618000
H	-0.47070200	1.97697800	-4.00076300
H	-0.83205000	0.44341800	-4.80261400
Pd	0.82671200	-1.43943700	-0.51932500
C	2.36381100	-0.24937500	0.00314900
C	2.24730200	0.97586600	0.56520100
C	3.44341200	1.84905400	0.81477600
C	3.68401700	2.38121700	2.09284700
C	4.32704400	2.18700300	-0.22285300
C	4.78088400	3.20981000	2.32972500
H	3.01458600	2.13557500	2.91288900
C	5.41867500	3.02289300	0.01129300
H	4.14938700	1.79364800	-1.21828600
C	5.65209500	3.53548800	1.28866900
H	4.95311400	3.60231000	3.32815800
H	6.08603300	3.27620600	-0.80775700
H	6.50314200	4.18552800	1.47074800
C	3.62329800	-0.93031800	-0.38877400
C	3.85984100	-1.24937500	-1.74157500
C	4.58491500	-1.31422200	0.56524400
C	5.02413200	-1.91410500	-2.12205000
H	3.12119200	-0.96296900	-2.48332000
C	5.74685200	-1.98143600	0.17937000
H	4.42586000	-1.07460200	1.61238700
C	5.97168300	-2.28509000	-1.16479500
H	5.19157200	-2.14409700	-3.17071400
H	6.47959100	-2.26203200	0.93116500
H	6.87706300	-2.80558500	-1.46363600
C	0.90184600	1.59249600	0.91172000
H	0.12713200	0.82261800	0.91077900
H	0.94611600	2.00820800	1.92561200
C	1.33108100	-2.41490200	2.38262100
O	0.93290500	-2.66772000	1.24062500
O	1.89503800	-1.27875900	2.73996000
H	2.00759400	-0.69426000	1.93878400

C	1.21258900	-3.41429100	3.49863500
H	0.65169500	-4.28448500	3.16135800
H	0.72371100	-2.95141200	4.36018900
H	2.21495600	-3.71898300	3.81526300

TS6

Thermal correction to Gibbs Free Energy= 0.554293 Hartree

SCF Done: E(RM06) = -2320.89874506 Hartree

0 1

C	2.28035800	-3.18144700	-0.09346000
C	2.35320400	-4.56706500	-0.19379200
C	1.97069500	-5.34195300	0.89825600
C	1.52449200	-4.70676300	2.05953300
C	1.47015900	-3.31649700	2.09187100
H	2.70825600	-4.98465500	-1.12908600
H	2.02068100	-6.42551600	0.84977400
H	1.22222500	-5.27340300	2.93342600
H	1.14727200	-2.75962300	2.96323700
N	1.83513500	-2.58133100	1.02933200
C	2.65718500	-2.28049700	-1.23950800
O	2.93568200	-2.77303700	-2.33980900
N	2.61034600	-0.97043700	-0.90843300
Pd	1.93789200	-0.53857000	0.95011700
C	-0.06431900	-0.11569800	3.10747300
O	-0.94876800	0.21385100	2.26428500
O	1.16655600	-0.34869500	2.88807300
C	-0.54048000	-0.26012400	4.54151300
H	-0.63011800	0.74206100	4.97419500
H	0.16499800	-0.83916200	5.13903100
H	-1.53335200	-0.71475500	4.55677600
C	2.75350000	0.00158400	-1.98309000
C	1.41793000	0.69786500	-2.39224400
H	3.12210200	-0.56992700	-2.84714100
C	0.21402000	-0.25197900	-2.22329800
H	1.27159900	1.54656900	-1.71518400
H	0.31959700	-1.10080200	-2.91043300
H	0.23217900	-0.66279000	-1.21237500
C	3.84762000	1.02956900	-1.66967300
O	3.79814000	2.21352100	-1.93570600
O	4.92681000	0.44212500	-1.11824900
C	1.51805600	1.23735500	-3.82777800
H	1.64131500	0.41639700	-4.54543300
H	2.36565100	1.91808200	-3.92663600
H	0.61467500	1.79011300	-4.10686300
C	6.00698300	1.32800300	-0.79455600
H	6.80784800	0.69110700	-0.41865200
H	5.69502200	2.04043600	-0.02654300
H	6.33640900	1.87997100	-1.67865700
C	1.96647200	2.46082900	1.34810100
O	0.75863900	2.58756800	1.68544100
O	2.51192700	1.43322800	0.82556400
C	2.89706100	3.63961400	1.55811500
H	2.35177000	4.50129100	1.94267300
H	3.36464700	3.88303900	0.59937000
H	3.69310600	3.35838400	2.25408000
Pd	-0.81307700	1.56181500	0.67579400
C	-2.48201000	0.61106900	-0.29945700
C	-2.26038000	-0.02459600	-1.51303200
C	-3.13369300	-1.09764600	-2.05796700
C	-2.58344600	-2.14922600	-2.81668100
C	-4.53346400	-1.05223000	-1.90527700
C	-3.39710300	-3.13896000	-3.36557800
H	-1.51057100	-2.20633300	-2.96122800
C	-5.34579000	-2.02532600	-2.48088600
H	-4.98448500	-0.23855900	-1.35008300
C	-4.78196200	-3.07921300	-3.20393800
H	-2.94694900	-3.95080100	-3.92952700
H	-6.42357700	-1.95953000	-2.36399300
H	-5.41752100	-3.84205800	-3.64431500
C	-3.45129300	0.13726700	0.72503000
C	-4.36153900	1.04201500	1.30048000
C	-3.45612500	-1.19205600	1.18507800
C	-5.26357900	0.62563000	2.27987200
H	-4.36298400	2.07666600	0.96976500
C	-4.35347200	-1.60619200	2.16584400
H	-2.74508200	-1.89805100	0.76784300
C	-5.26482900	-0.70022900	2.71641300
H	-5.96459000	1.33975800	2.70317600
H	-4.34181000	-2.63889400	2.50445700
H	-5.96515900	-1.02476100	3.48090200
C	-1.15403100	0.45946700	-2.43560400
H	-1.47969800	0.35762900	-3.47520100
H	-1.00378500	1.52273300	-2.24127300
C	-1.81662500	3.70366300	-1.04470400
O	-0.74069300	3.20109600	-0.58403600
O	-2.93862600	3.11781400	-1.04450200
H	-2.68761300	1.84366700	-0.63007100
C	-1.72699500	5.10157000	-1.61718500

H	-0.70996500	5.31386400	-1.94806600
H	-1.99074800	5.81377300	-0.82790300
H	-2.43815500	5.22063600	-2.43597500

TS6’

Thermal correction to Gibbs Free Energy= 0.555063 Hartree

SCF Done: E(RM06) = -2320.91161806 Hartree

0 1

C	-3.39962000	-0.15274200	2.10377500
C	-4.03508200	-0.56412700	3.27114300
C	-4.35970200	-1.90999000	3.42010900
C	-4.04701200	-2.80314000	2.39246200
C	-3.41875600	-2.32516300	1.24635500
H	-4.25657800	0.19130800	4.01624900
H	-4.85328700	-2.26094300	4.32130000
H	-4.28755100	-3.85793500	2.46779400
H	-3.16188600	-2.95465400	0.40353700
N	-3.10504600	-1.02532100	1.12246000
C	-3.03987900	1.28612700	1.85939700
O	-3.41485600	2.14856700	2.66356600
N	-2.31726000	1.47818800	0.72827100
Pd	-2.16655300	-0.18749600	-0.47390300
C	-1.58454000	-2.61855700	-2.19752000
O	-0.33732900	-2.52214700	-2.08096400
O	-2.46712200	-1.93298800	-1.58208000
C	-2.11431000	-3.63884500	-3.19021400
H	-3.05525000	-4.06405600	-2.83545800
H	-1.37595100	-4.42264500	-3.36211000
H	-2.31437200	-3.13051700	-4.13961500
C	-2.07902200	2.88124300	0.36852700
C	-0.73577100	3.51010200	0.82704700
H	-2.86628700	3.45261100	0.88343300
C	0.54459300	2.76514700	0.38975200
H	-0.70325000	4.46680000	0.29240200
H	0.45388800	2.46949900	-0.65722300
H	1.36982100	3.48557100	0.42859900
C	-2.35020100	3.14421500	-1.11622700
O	-1.65551200	3.82242700	-1.84720700
O	-3.52974800	2.61586000	-1.49330000
C	-0.74023900	3.83104500	2.32996500
H	-0.86163500	2.93841900	2.94845300
H	-1.56527400	4.50260000	2.58382600
H	0.19777500	4.32268400	2.61412600
C	-3.87475600	2.78999800	-2.87609300
H	-4.88417900	2.39200800	-2.97934100
H	-3.17745700	2.22866000	-3.50168400
H	-3.84592600	3.84673500	-3.15226900
C	-0.10521100	0.76648500	-2.54847500
O	0.89444600	0.14174600	-2.08124800
O	-1.30140200	0.74872500	-2.12079600
C	0.14557900	1.65430100	-3.75140900
H	1.20617300	1.67845600	-4.00122000
H	-0.22564600	2.65850000	-3.53050900
H	-0.42129300	1.26715900	-4.60402600
Pd	0.74784600	-1.36339800	-0.66539200
C	2.31229100	-0.40305000	0.41395700
C	2.24148500	0.92355900	0.78146000
C	3.44113700	1.80246000	0.85420200
C	3.76864500	2.45562100	2.05648700
C	4.24878400	2.02693300	-0.27357500
C	4.88494100	3.28601800	2.13400400
H	3.16205800	2.29168300	2.94197200
C	5.34953200	2.87925300	-0.19953900
H	3.99672700	1.54814200	-1.21196800
C	5.67649900	3.50489200	1.00454700
H	5.13354400	3.76608100	3.07608400
H	5.95414100	3.05146300	-1.08523400
H	6.54069700	4.16026100	1.06263000
C	3.58575100	-1.09112500	0.02319500
C	3.99655000	-1.17044600	-1.31954600
C	4.36890400	-1.72766900	1.00064200
C	5.16468900	-1.84764100	-1.66820200
H	3.38541100	-0.70447600	-2.08692700
C	5.53890800	-2.40245800	0.64934200
H	4.05442000	-1.69105800	2.03942800
C	5.94194600	-2.46498900	-0.68544700
H	5.46682800	-1.89506800	-2.71091200
H	6.13534600	-2.88126900	1.42131300
H	6.85125400	-2.99286400	-0.95857300
C	0.94388500	1.53767400	1.24256700
H	0.15267900	0.78664100	1.22983200
H	1.07168700	1.86540100	2.28306000
C	0.83329800	-2.85559500	1.87402400
O	0.53605700	-2.95169900	0.63848900
O	1.35183800	-1.84335300	2.42169500
H	1.74098200	-0.99413600	1.36959300
C	0.56639100	-4.07668800	2.73286600
H	-0.14161300	-4.74846100	2.24727500

H	0.19929900	-3.76767600	3.71370500
H	1.51213000	-4.60805800	2.88218900

12

Thermal correction to Gibbs Free Energy= 0.607601 Hartree

SCF Done: E(RM06) = -2549.97468962 Hartree

0 1

Pd	-3.46151100	-1.40775300	0.90448900
O	-4.21340400	-0.07015100	-1.97026000
C	-4.96758700	-1.02373400	-1.60288600
O	-4.83622300	-1.74641800	-0.57283200
C	-6.14017000	-1.34970800	-2.50664000
H	-5.77598100	-1.95024800	-3.34685000
H	-6.89600000	-1.91887400	-1.96547900
H	-6.56515600	-0.43044400	-2.91483200
Pd	-2.29658400	0.36685900	-1.23867600
C	-5.52579800	-0.52151800	2.60135000
O	-4.67876600	-0.03396400	1.71346200
O	-5.57466600	-1.68855600	2.96271600
C	-6.47696600	0.54480300	3.13512600
H	-7.02219900	0.15069000	3.99361800
H	-5.93060500	1.44914100	3.41689900
H	-7.19133900	0.82168300	2.35267300
C	-1.52753500	-0.34662000	3.02342200
O	-1.06179600	0.63179800	2.26952300
O	-2.24602700	-1.26802100	2.62706800
C	-1.64649600	-2.58302500	-1.13705000
O	-2.18263100	-2.76076800	-0.00415000
O	-1.62169400	-1.50451700	-1.81637400
C	-0.95758300	-3.77205700	-1.77175500
H	0.01459500	-3.46003700	-2.16004600
H	-0.85167300	-4.58268700	-1.05101700
H	-1.56318900	-4.11950900	-2.61555400
C	-1.08219300	-0.26264500	4.45459500
H	-1.57920200	-1.03295200	5.04165300
H	0.00331700	-0.39162000	4.50403700
H	-1.31335000	0.72976700	4.85138000
C	-1.85063700	3.02529900	-0.28206500
C	-2.06988900	4.32563800	0.15854500
C	-3.37751300	4.80429900	0.20661400
C	-4.42326900	3.96932800	-0.19164100
C	-4.13424900	2.68025600	-0.63050100
H	-1.20691500	4.91702400	0.44215300
H	-3.58137600	5.81418600	0.54900400
H	-5.45438200	4.30377700	-0.16544400
H	-4.89397700	1.98235500	-0.95791100
N	-2.86903200	2.23487900	-0.67146800
C	-0.46949400	2.44010800	-0.38797300
O	0.51610600	3.15829200	-0.20926800
C	0.87598500	0.58518900	-1.04715000
C	1.65367000	-0.03247000	0.14764800
H	1.45283000	1.47252000	-1.34367900
C	3.17060900	-0.04005800	-0.14588900
H	1.48864900	0.68356900	0.96369000
C	4.04258300	-0.35085100	1.08790500
H	3.38760500	-0.76893500	-0.93376700
H	3.46033100	0.94474300	-0.53290300
H	3.82754000	-1.35770800	1.45961500
C	0.84637800	-0.27412500	-2.31188500
O	1.43438000	-1.32523700	-2.46944900
O	0.16256300	0.34415700	-3.29497900
C	1.14157800	-1.40718100	0.60185800
H	1.41618900	-2.18125400	-0.11815100
H	0.05532400	-1.42711000	0.71317300
H	1.56903700	-1.67932100	1.57224500
C	0.05344600	-0.38681300	-4.52752800
H	-0.46679200	0.27781500	-5.21699500
H	-0.52509100	-1.29854400	-4.36417300
H	1.04292000	-0.64492900	-4.91195800
H	3.76831700	0.34661400	1.89303600
N	-0.45981300	1.10611600	-0.68909800
H	-1.24108900	0.49759600	1.31042400
C	5.53299300	-0.23756000	0.81218300
C	6.29475500	-1.35295600	0.79959800
H	5.76309200	-2.29413900	0.94370700
C	7.74898200	-1.52553600	0.62447700
C	8.70577500	-0.56234500	0.99764000
C	8.21609600	-2.75038100	0.11093200
C	10.06750900	-0.80910000	0.83620300
H	8.38026400	0.37695400	1.42911500
C	9.57778200	-2.99425700	-0.05735900
H	7.49443800	-3.51577700	-0.16435200
C	10.51195500	-2.02138500	0.30237000
H	10.78680700	-0.05200800	1.13657000
H	9.90905800	-3.94583200	-0.46404100
H	11.57471500	-2.20892400	0.17837100
C	6.03833400	1.13421900	0.50847100
C	6.65127600	1.41686100	-0.72417700

C	5.86729800	2.18706500	1.42280700
C	7.09064800	2.70514000	-1.02594800
H	6.77907500	0.61521900	-1.44487700
C	6.31404500	3.47493100	1.12450200
H	5.39447800	1.99289300	2.38165500
C	6.92661000	3.73913500	-0.10160000
H	7.55878900	2.90232500	-1.98638400
H	6.18019000	4.27260900	1.84993100
H	7.26870300	4.74292000	-0.33682100

TS7

Thermal correction to Gibbs Free Energy= 0.605329 Hartree

SCF Done: E(RM06) = -2549.96110684 Hartree

0 1

Pd	-3.41139700	-1.11555300	1.20737800
O	-4.19638300	-0.64832300	-1.93795500
C	-4.94143000	-1.47418700	-1.30850600
O	-4.80404200	-1.86911600	-0.12027500
C	-6.10210300	-2.05071200	-2.09469700
H	-5.72972200	-2.87618500	-2.71058500
H	-6.86685500	-2.43215600	-1.41794800
H	-6.51746400	-1.29595200	-2.76537300
Pd	-2.33575300	-0.01412500	-1.26250500
C	-5.50432500	0.17648800	2.58170600
O	-4.64286000	0.42215400	1.61312500
O	-5.59078800	-0.87108400	3.20567800
C	-6.41499800	1.37089300	2.85617500
H	-7.10222200	1.12783100	3.66727800
H	-5.81764500	2.24693600	3.12689500
H	-6.98457400	1.62654700	1.95692600
C	-1.36329100	0.51190800	2.73460000
O	-0.90313800	1.17703900	1.74459600
O	-2.15353200	-0.46818600	2.70540600
C	-1.62002600	-2.80062500	-0.48449700
O	-2.12743400	-2.67166600	0.66182100
O	-1.61634300	-1.93360400	-1.42796100
C	-0.93696800	-4.11071200	-0.81320200
H	0.01953000	-3.90961900	-1.30083600
H	-0.79913900	-4.70407600	0.09048900
H	-1.56428800	-4.66837400	-1.51664300
C	-0.87920500	0.95207600	4.10175500
H	-1.40854300	0.41944300	4.89092700
H	0.19556000	0.76020300	4.18023700
H	-1.02593600	2.03061300	4.20652800
C	-1.97613600	2.81238200	-0.95796400
C	-2.23157600	4.17183100	-0.81987100
C	-3.55883500	4.59513500	-0.76504600
C	-4.57861600	3.64832900	-0.86052500
C	-4.25060700	2.30343600	-1.01883400
H	-1.39285300	4.85531400	-0.76045500
H	-3.79486800	5.64801300	-0.64756600
H	-5.62237700	3.93758700	-0.81157200
H	-4.99252900	1.52133500	-1.10183000
N	-2.97002500	1.91104000	-1.07109900
C	-0.58208900	2.26707800	-1.02838000
O	0.35367500	2.98677300	-1.32330000
C	0.86680300	0.32124800	-0.99024900
C	1.66614800	-0.08341800	0.27575100
H	1.41934500	1.15395800	-1.44096300
C	3.17685400	-0.12257100	-0.05185500
H	1.51116600	0.75055800	0.97300000
C	4.08109300	-0.23756900	1.19262600
H	3.38504400	-0.95986900	-0.72564800
H	3.44796300	0.79450200	-0.59027200
H	3.90632300	-1.19144700	1.69952700
C	0.82734400	-0.73001400	-2.10253000
O	1.43452800	-1.77980400	-2.10488600
O	0.11262800	-0.28089800	-3.15312700
C	1.18273900	-1.36962000	0.96269200
H	1.42539800	-2.24625900	0.35801000
H	0.10647800	-1.36561400	1.14424400
H	1.66629000	-1.48597200	1.93724100
C	-0.01490000	-1.19882800	-4.25492900
H	-0.56974500	-0.65910900	-5.02177900
H	-0.56649000	-2.08321100	-3.93035900
H	0.97025000	-1.49248500	-4.62380300
H	3.80090900	0.55276200	1.90419000
N	-0.49298500	0.88722800	-0.69445000
H	-0.77301700	0.87757700	0.53873600
C	5.56017100	-0.11657300	0.86339500
C	6.35265000	-1.20695800	0.94727300
H	5.85253900	-2.13963600	1.21038400
C	7.80502100	-1.36113700	0.74262600
C	8.74493300	-0.33878700	0.97414300
C	8.29016500	-2.62307300	0.34989400
C	10.10728200	-0.56755000	0.79252700
H	8.40681800	0.63374500	1.31213800
C	9.65209800	-2.84958300	0.16091400

H	7.58239600	-3.43212600	0.18599900
C	10.56884500	-1.81969000	0.37881100
H	10.81385700	0.23570700	0.98297000
H	9.99743200	-3.83155100	-0.15060000
H	11.63198500	-1.99299000	0.23858100
C	6.01569800	1.22737200	0.39848900
C	6.57650800	1.39400800	-0.87926400
C	5.84724800	2.36590900	1.20434300
C	6.96765900	2.65411500	-1.33011900
H	6.70302500	0.52427400	-1.51644000
C	6.24623200	3.62602200	0.75685300
H	5.41665700	2.26114900	2.19650200
C	6.80677200	3.77512300	-0.51286200
H	7.39630100	2.76080100	-2.32275300
H	6.11732300	4.49165700	1.40073800
H	7.11219100	4.75666100	-0.86388800

13

Thermal correction to Gibbs Free Energy= 0.608173 Hartree

SCF Done: E(RM06) = -2549.96489165 Hartree

0 1

Pd	-3.42387100	-1.08491000	1.22555600
O	-4.19628800	-0.62464900	-1.95251000
C	-4.93412400	-1.46359700	-1.32365200
O	-4.78479500	-1.86332700	-0.14303100
C	-6.09280900	-2.03570800	-2.11581500
H	-5.71592700	-2.84520500	-2.74986700
H	-6.84936500	-2.43667900	-1.44118900
H	-6.51993000	-1.27257200	-2.76949700
Pd	-2.33648400	-0.02604500	-1.28097900
C	-5.59557900	0.16158700	2.52666200
O	-4.68955300	0.43451500	1.60792600
O	-5.71855200	-0.90474100	3.11103200
C	-6.50781700	1.35492200	2.80505600
H	-7.27456000	1.06923100	3.52601300
H	-5.92076900	2.18810500	3.20422300
H	-6.98131500	1.69960000	1.88022900
C	-1.44094500	0.52814500	2.76226500
O	-1.01974900	1.16652700	1.76510700
O	-2.24393700	-0.46531300	2.76706700
C	-1.62128000	-2.78461500	-0.47463900
O	-2.09836800	-2.62107500	0.67654900
O	-1.63775700	-1.94391700	-1.44781300
C	-0.94496800	-4.10237500	-0.78764900
H	0.01186900	-3.91394600	-1.27996200
H	-0.80726800	-4.68192100	0.12491800
H	-1.57598400	-4.66737700	-1.48172300
C	-0.98632000	0.96648700	4.14660200
H	-0.97699400	0.12184400	4.83706100
H	-0.00253100	1.43592500	4.09179900
H	-1.70066500	1.70457600	4.52789200
C	-1.95243900	2.80790300	-0.98137300
C	-2.19612600	4.16539400	-0.81208200
C	-3.51754100	4.58601500	-0.66209300
C	-4.53954800	3.63892900	-0.69790200
C	-4.22367200	2.29531700	-0.89584500
H	-1.35782100	4.85176200	-0.79873100
H	-3.74520400	5.63692800	-0.51521700
H	-5.57736100	3.92519200	-0.57013400
H	-4.97044100	1.51465700	-0.92760200
N	-2.95045900	1.90485800	-1.04155600
C	-0.56805400	2.26716400	-1.14743000
O	0.33784600	2.94864500	-1.57406300
C	0.91919000	0.31580900	-1.04497800
C	1.69820300	-0.05869600	0.24104700
H	1.46823900	1.13893200	-1.51608300
C	3.21173500	-0.11636500	-0.06840400
H	1.53677800	0.79330800	0.91594200
C	4.10041100	-0.21146700	1.18876700
H	3.42001700	-0.97014600	-0.72134400
H	3.49699800	0.78618300	-0.62404000
H	3.91551200	-1.15503000	1.71080500
C	0.86457900	-0.76217600	-2.13144900
O	1.46468000	-1.81489600	-2.11030600
O	0.14110600	-0.33187800	-3.18216500
C	1.19204000	-1.32138800	0.95423600
H	1.42248000	-2.21588800	0.37129900
H	0.11643900	-1.29108100	1.13607600
H	1.67108700	-1.41996400	1.93247500
C	-0.01247500	-1.27473900	-4.26095000
H	-0.56254000	-0.74295900	-5.03654000
H	-0.57918000	-2.13856900	-3.90886100
H	0.96447800	-1.59561900	-4.62845100
H	3.81470900	0.59256100	1.88236400
N	-0.44181000	0.88510200	-0.75942400
H	-0.68604100	0.85497200	0.30009100
C	5.58350400	-0.10181600	0.87381000
C	6.37090700	-1.19311100	0.98781300

H	5.86441100	-2.11875500	1.26320400
C	7.82499300	-1.35605000	0.80322600
C	8.76568100	-0.33270900	1.02687700
C	8.30999400	-2.62670900	0.43961300
C	10.12922300	-0.56939000	0.86552700
H	8.42715100	0.64696200	1.34316400
C	9.67320900	-2.86122800	0.27076500
H	7.60131600	-3.43633200	0.28265100
C	10.59098600	-1.83053100	0.48036100
H	10.83645200	0.23476100	1.04957100
H	10.01866900	-3.84995500	-0.01837200
H	11.65505600	-2.01001300	0.35592800
C	6.04860400	1.23162600	0.38852200
C	6.62606800	1.37187300	-0.88501500
C	5.87267300	2.38623700	1.16965100
C	7.02609000	2.62199700	-1.35552400
H	6.75902500	0.48941000	-1.50311200
C	6.28048400	3.63640900	0.70256600
H	5.42977600	2.30199300	2.15834100
C	6.85752100	3.75925300	-0.56259100
H	7.46797500	2.70811600	-2.34434000
H	6.14604200	4.51473000	1.32787300
H	7.17020500	4.73294100	-0.92874700

3-a

Thermal correction to Gibbs Free Energy= 0.429359 Hartree

SCF Done: E(RM06) = -1380.33251494 Hartree

0 1

C	5.45844000	-1.25382500	0.01659500
C	6.06583900	-2.51143100	0.02012400
C	7.42161000	-2.58889300	0.33045800
C	8.11213400	-1.41279900	0.62416900
C	7.41333600	-0.20368500	0.59424700
H	5.46553200	-3.38166700	-0.21822100
H	7.93132300	-3.54791700	0.34358400
H	9.16877000	-1.42594400	0.87171600
H	7.92038300	0.73289600	0.81803000
N	6.11312100	-0.11604300	0.29753800
C	3.98838800	-1.15476400	-0.31946600
O	3.32007800	-2.15310000	-0.58780600
C	2.11139900	0.39012500	-0.63305600
C	1.14964800	0.16535300	0.58172300
H	1.81036400	-0.31525000	-1.41369900
C	-0.32620900	0.15806400	0.13892900
H	1.40357800	-0.84693700	0.92133500
C	-1.28470700	-0.41114500	1.20608400
H	-0.64020900	1.16918800	-0.14128300
H	-0.42556400	-0.45239600	-0.76719200
C	1.95781600	1.78695800	-1.22178900
O	1.02510700	2.13461900	-1.91163100
O	2.95947900	2.61822200	-0.85221100
C	1.43290300	1.14936100	1.72533900
H	1.13232600	2.17010000	1.46106000
H	2.49707800	1.16341600	1.97961800
H	0.88579200	0.86988700	2.63004800
C	2.86085100	3.96408800	-1.35133900
H	3.73533700	4.48614000	-0.96410200
H	1.94091700	4.43560500	-0.99801600
H	2.86228900	3.96653800	-2.44379400
H	-0.97086200	-1.42792700	1.47043000
H	-1.20860300	0.18919600	2.12182700
N	3.49570900	0.10849000	-0.29597700
H	4.14086600	0.85968300	-0.08666200
C	-2.73384300	-0.43165400	0.74963900
C	-3.32055000	-1.60833300	0.44183300
H	-2.68104700	-2.48947200	0.50454100
C	-4.70159400	-1.91799200	0.02769300
C	-4.92099200	-3.08367000	-0.73085500
C	-5.82459100	-1.15114700	0.39280700
C	-6.20103200	-3.45322100	-1.13915800
H	-4.06934600	-3.70104400	-1.00638200
C	-7.10523300	-1.52488200	-0.00923000
H	-5.69409100	-0.26744000	1.00618300
C	-7.30146900	-2.67212000	-0.78209800
H	-6.33927100	-4.35375600	-1.73121500
H	-7.95669700	-0.91947700	0.28959100
H	-8.30191900	-2.95893900	-1.09329300
C	-3.38791900	0.90396600	0.61380100
C	-3.50934800	1.76319000	1.71877200
C	-3.85061500	1.35444900	-0.63394700
C	-4.09431100	3.02314000	1.58559900
H	-3.15963500	1.43454900	2.69383600
C	-4.42694700	2.61689000	-0.76933500
H	-3.75187900	0.70551700	-1.49843200
C	-4.55409900	3.45524000	0.34028900
H	-4.18976600	3.66730700	2.45545100
H	-4.77386600	2.94735200	-1.74438900
H	-5.00436700	4.43816400	0.23471300

TS1-a

Thermal correction to Gibbs Free Energy= 0.375469 Hartree

SCF Done: E(RM06) = -1781.66626306 Hartree

0 1

Pd	-2.09207500	-0.02282600	1.31526500
O	-2.03635300	-0.95971800	-1.99781900
C	-3.15939400	-0.89550700	-1.39625700
O	-3.39246300	-0.48029000	-0.22809300
C	-4.36118200	-1.38701900	-2.18236600
H	-5.25431900	-0.83243000	-1.89031500
H	-4.18363300	-1.30541000	-3.25556400
H	-4.52377500	-2.44200700	-1.93690300
Pd	-0.16217800	-0.43622000	-1.25645200
C	-1.02444500	1.94400400	2.33671200
O	-1.74724300	2.08493200	1.29059000
O	-0.93810700	0.76999900	2.83930000
C	-0.24765600	3.08262500	2.92037300
H	-0.73375500	4.03236500	2.68979000
H	-0.14330400	2.95322800	3.99931400
H	0.75255700	3.07131900	2.47090300
C	-1.12931300	-2.67177300	1.61530900
O	-2.18177300	-1.95863100	1.86785000
C	-1.15043500	-4.03906600	2.28324400
H	-0.76933700	-3.93391200	3.30465600
H	-2.16762700	-4.43074500	2.34376100
H	-0.50330900	-4.73001900	1.74050000
C	0.52404300	2.28229500	-0.89634700
C	0.50516400	3.67248100	-0.90972900
C	-0.52189400	4.31949700	-1.59188500
C	-1.50496500	3.55904000	-2.22909500
C	-1.43379800	2.17120100	-2.16488200
H	1.29532500	4.19839500	-0.38661300
H	-0.56039400	5.40384400	-1.62734400
H	-2.32122200	4.02694900	-2.76792800
H	-2.16478700	1.52270600	-2.63154900
N	-0.42960100	1.56715800	-1.51433600
C	1.60232000	1.49136100	-0.20857800
O	2.34698500	2.05545800	0.60103500
C	2.71028000	-0.67919000	-0.09775700
C	3.15274200	-1.81487800	-1.08468900
H	2.38850500	-1.12417500	0.85102900
C	2.17788000	-3.00285200	-1.22818600
H	3.28602300	-1.33593600	-2.06362200
C	0.84155900	-2.74667200	-1.91384900
H	2.70499700	-3.76719800	-1.81296200
H	1.98717100	-3.43835000	-0.24106000
H	0.93354200	-2.13293300	-2.81510100
C	3.96840300	0.17780600	0.16252100
O	4.57463000	0.78337100	-0.69364500
O	4.37648600	0.05374700	1.43913700
C	4.51293300	-2.40529700	-0.64828200
H	4.46239200	-2.80098400	0.37246000
H	5.31656700	-1.66888000	-0.69308300
H	4.78950700	-3.23132300	-1.31039700
C	5.52769700	0.83984400	1.78187700
H	5.73273600	0.62165800	2.83008100
H	5.30616600	1.90113500	1.64533300
H	6.38395100	0.57063600	1.15784600
H	0.34368700	-3.67742700	-2.20866100
N	1.65177200	0.19794300	-0.62273500
O	-0.16130700	-2.31352400	0.92628500
H	0.11632200	-2.36030500	-1.15638000

1a

Thermal correction to Gibbs Free Energy= 0.373924 Hartree

SCF Done: E(RM06) = -1781.67106392 Hartree

0 1

Pd	-2.14140700	-0.17886300	1.16036900
O	-1.91380500	0.66386100	-2.21898600
C	-3.05457900	0.68366400	-1.64071300
O	-3.31050200	0.48653700	-0.42482000
C	-4.24420600	0.96511800	-2.54071800
H	-5.01105100	1.50982800	-1.98768800
H	-3.93893500	1.51586000	-3.43144500
H	-4.67241600	0.00708900	-2.85432900
Pd	-0.07999500	0.29407000	-1.31103400
C	-0.54345600	0.69816600	2.82101500
O	-0.96283900	1.45564300	1.87733000
O	-0.99993900	-0.49519400	2.86444900
C	0.47529000	1.17409700	3.80990000
H	0.23403800	2.19030700	4.13159500
H	0.51323000	0.50054500	4.66713300
H	1.45442700	1.19307200	3.31758800
C	-2.45259800	-2.96922000	0.73886300
O	-3.15690200	-1.90486800	1.01616300
C	-3.24685600	-4.25836200	0.90748600
H	-3.29642500	-4.50630600	1.97298400

H	-4.27125900	-4.13821700	0.54774400
H	-2.74722400	-5.07157100	0.37871800
C	1.27087900	2.40740000	-0.01107500
C	1.60409300	3.66055600	0.48890300
C	0.85885900	4.76312200	0.07824100
C	-0.19995800	4.58014400	-0.81432300
C	-0.48727900	3.29631700	-1.26793000
H	2.43533000	3.72745600	1.18120400
H	1.09710400	5.75561400	0.44807500
H	-0.80190300	5.41452400	-1.15642100
H	-1.29809100	3.07844000	-1.95251900
N	0.24775200	2.24980600	-0.86742800
C	2.04114000	1.16554700	0.34225200
O	2.89002700	1.19823500	1.24110300
C	2.51470400	-1.12641600	-0.27740000
C	2.47642100	-2.07300500	-1.52140700
H	2.17193200	-1.67855500	0.60690700
C	1.14805100	-2.83264700	-1.70681600
H	2.65710900	-1.43730500	-2.39891100
C	-0.06146200	-2.00072100	-2.10884900
H	1.30921600	-3.57285300	-2.50071500
H	0.89820000	-3.39166300	-0.79934700
H	0.14553200	-1.35064700	-2.96801800
C	3.98947700	-0.73406300	-0.05287400
O	4.63847300	-0.04306800	-0.80651400
O	4.49094900	-1.35440900	1.03085600
C	3.60890700	-3.11859200	-1.44706600
H	3.53553600	-3.71894800	-0.53380400
H	4.59782200	-2.65666600	-1.47569100
H	3.54153600	-3.79989800	-2.30052100
C	5.86020500	-1.04012300	1.32803300
H	6.11093000	-1.62310000	2.21426100
H	5.96222800	0.02903400	1.52909900
H	6.51204600	-1.30956900	0.49284400
H	-0.92819900	-2.61120200	-2.37605100
N	1.73419400	0.10566300	-0.44558400
O	-1.26943200	-2.97630400	0.39336800
H	-0.49386500	-1.53245400	-1.16774900

TS2-a

Thermal correction to Gibbs Free Energy= 0.368407 Hartree

SCF Done: E(RM06) = -1781.64650679 Hartree

0 1

Pd	-2.56890000	-0.47876100	0.75646500
O	-1.49682900	-0.58660800	-2.30341800
C	-2.58033500	-1.20028400	-2.08970600
O	-3.12480100	-1.42720700	-0.96250300
C	-3.31463800	-1.74602600	-3.29997900
H	-3.27933500	-1.01565600	-4.11128000
H	-2.79978500	-2.64881900	-3.64490600
H	-4.34602100	-1.99561400	-3.05139400
Pd	0.18901000	-0.20586600	-1.06053500
C	-2.80590700	1.78650300	1.66319400
O	-3.11592800	1.52382800	0.44754200
O	-2.34435200	0.82855000	2.37085500
C	-2.99873100	3.15716700	2.23480900
H	-2.46889500	3.24667900	3.18398300
H	-2.64255300	3.90661500	1.52412300
H	-4.06731900	3.33011800	2.39987200
C	-0.94356400	-2.67649900	1.82067100
O	-2.13069900	-2.26750200	1.62237900
C	-0.78002800	-3.76549600	2.86119000
H	-0.20282100	-3.36053000	3.69847100
H	-1.74778000	-4.11404000	3.22078100
H	-0.20679400	-4.59416800	2.43760000
C	0.81836800	2.45699800	-0.21061000
C	0.78959800	3.82740900	0.02874300
C	-0.18727300	4.59540800	-0.60313500
C	-1.11294700	3.96940600	-1.44115000
C	-1.03818800	2.58900400	-1.61548800
H	1.54162400	4.24336000	0.68926300
H	-0.22566500	5.66986700	-0.44934000
H	-1.88632100	4.53309200	-1.95121800
H	-1.73805900	2.03815500	-2.23269900
N	-0.08481300	1.86555900	-1.01432100
C	1.89646400	1.57774800	0.37007500
O	2.71185100	2.05486600	1.16724800
C	3.02640800	-0.54988400	0.20352000
C	3.24331000	-1.69135700	-0.83690200
H	2.89049200	-0.99036200	1.19968600
C	2.13637100	-2.75992200	-0.81212400
H	3.25902500	-1.20958700	-1.82383100
C	0.75466800	-2.30179900	-1.26971200
H	2.45509700	-3.57463100	-1.47777900
H	2.07634000	-3.18775400	0.19391500
H	0.78109700	-2.03367700	-2.33330900
C	4.31771400	0.28952800	0.22895200
O	4.72860100	0.94983900	-0.70003800

O	4.99778400	0.09479500	1.37624200
C	4.60059900	-2.38753300	-0.61755000
H	4.67599800	-2.80100400	0.39458800
H	5.44108500	-1.70696700	-0.77043900
H	4.71550300	-3.21461400	-1.32510900
C	6.20672100	0.85789700	1.49642100
H	6.63640900	0.58208900	2.45976200
H	5.97755800	1.92606200	1.47012400
H	6.90015600	0.62262100	0.68449200
H	0.00996500	-3.10371400	-1.20202500
N	1.89854200	0.31978200	-0.13190500
O	0.10695100	-2.25490500	1.25276800
H	0.19756900	-1.84695900	-0.00844400

2a

Thermal correction to Gibbs Free Energy= 0.372957 Hartree

SCF Done: E(RM06) = -1781.68032781 Hartree

0 1

Pd	-2.54502800	-0.55207900	0.65834500
O	-1.46483400	-0.81310400	-2.34856700
C	-2.48084200	-1.50084600	-2.09360100
O	-3.00460600	-1.70427300	-0.93820100
C	-3.17889900	-2.20420100	-3.24173200
H	-3.10996900	-1.59111100	-4.14221600
H	-2.65882700	-3.14823700	-3.43675200
H	-4.21938500	-2.42061700	-2.99809600
Pd	0.21491200	-0.30211800	-1.11502400
C	-3.16842300	1.70037800	1.37709200
O	-3.47500000	1.25957600	0.21345600
O	-2.51271900	0.91005100	2.14129900
C	-3.53681800	3.08321500	1.80789500
H	-3.46760200	3.17032600	2.89308100
H	-2.84139000	3.79143600	1.34529600
H	-4.54538400	3.32388000	1.46398100
C	-0.66128300	-2.22166300	2.27426800
O	-1.75093600	-2.20200000	1.67695300
C	-0.37903300	-3.25917700	3.32135800
H	0.03995400	-2.77792600	4.20879500
H	-1.29087500	-3.79964600	3.56993200
H	0.37365400	-3.95563600	2.93815900
C	0.73619200	2.42905800	-0.14613000
C	0.72000700	3.79546200	0.13329300
C	-0.27269800	4.58478200	-0.44318400
C	-1.22301400	3.98410500	-1.27301200
C	-1.15034400	2.60819300	-1.48767200
H	1.49673300	4.19193400	0.77654800
H	-0.30160200	5.65483100	-0.25798000
H	-2.00527400	4.56465200	-1.75073600
H	-1.86954900	2.08459200	-2.10845200
N	-0.19025900	1.85528700	-0.93476300
C	1.85584200	1.56202600	0.38140500
O	2.70847400	2.07447800	1.12164000
C	3.07594000	-0.47927100	0.23878300
C	3.22764900	-1.74876200	-0.64052200
H	3.08170000	-0.77754900	1.29627700
C	2.01896700	-2.69178200	-0.50763900
H	3.28901500	-1.40454800	-1.68174000
C	0.77596900	-2.25774700	-1.26635400
H	2.32403100	-3.67972300	-0.88733800
H	1.80257200	-2.83815900	0.55855300
H	0.92666000	-2.36332600	-2.34883100
C	4.31896800	0.39792700	0.00909700
O	4.56454700	0.99530200	-1.01593700
O	5.16956300	0.32486400	1.05581200
C	4.52558800	-2.50108500	-0.29830800
H	4.52410100	-2.83934800	0.74485400
H	5.41652400	-1.88549800	-0.44449100
H	4.62604800	-3.38513700	-0.93600100
C	6.34890900	1.13040300	0.92972600
H	6.92993500	0.94982700	1.83471900
H	6.07580400	2.18606000	0.85500300
H	6.92277200	0.84960300	0.04232900
H	-0.09892300	-2.85560300	-0.98318200
N	1.86439800	0.28469800	-0.06951300
O	0.32839800	-1.38876200	2.06832900
H	0.22649000	-0.84705400	1.23862800

^{M}1

Thermal correction to Gibbs Free Energy= 0.283185 Hartree

SCF Done: E(RM06) = -1196.88173265 Hartree

0 1

C	1.33815200	2.15557700	-0.35553300
C	1.86472200	3.43648400	-0.48106100
C	3.23557300	3.61600900	-0.31083200
C	4.04073400	2.51120000	-0.01936400
C	3.45014300	1.25603600	0.09176400
H	1.17750900	4.24477600	-0.70421800
H	3.67596000	4.60422600	-0.40187200

H	5.11108600	2.61287900	0.12107100
H	4.01362600	0.35572900	0.31298400
N	2.12721600	1.10368500	-0.07598500
C	-0.12758900	1.85953200	-0.49769600
O	-0.92588400	2.76978100	-0.74628300
N	-0.42877000	0.55358600	-0.31507100
Pd	1.15958000	-0.67162700	0.02417400
C	1.85535000	-3.03402100	0.31550800
O	0.62779200	-2.74140800	0.14049100
O	2.70365000	-2.07509500	0.34445500
C	2.29990300	-4.46067400	0.45294500
H	1.52217200	-5.05032900	0.94191600
H	3.23477600	-4.51304300	1.01356300
H	2.46926100	-4.87939400	-0.54510700
C	-1.85805200	0.24687600	-0.37403400
C	-2.16995800	-1.27760300	-0.31747500
H	-2.24515100	0.64991900	-1.31599000
C	-3.48972400	-1.62591400	-1.04041700
H	-1.35871100	-1.76808200	-0.86673900
H	-3.72419300	-2.67288000	-0.80889100
H	-4.30583400	-1.02772400	-0.61858800
C	-2.59150900	1.00680100	0.74317500
O	-2.13504300	1.29953300	1.82480000
O	-3.86615100	1.27466600	0.37499800
C	-4.64729300	1.97810100	1.35351700
H	-5.63219000	2.11130000	0.90547500
H	-4.19321700	2.94659500	1.57681600
H	-4.72104000	1.40170300	2.27942500
C	-3.45608700	-1.45771600	-2.56380900
H	-2.66030700	-2.06471400	-3.01116000
H	-4.40499300	-1.77207100	-3.01110900
H	-3.28743900	-0.41806100	-2.86332800
C	-2.17606500	-1.83412200	1.11568600
H	-1.29451200	-1.52192900	1.68026100
H	-3.06093800	-1.49660400	1.66742300
H	-2.19038500	-2.92756500	1.09150500

^{M}TS1-δ

Thermal correction to Gibbs Free Energy= 0.282859 Hartree

SCF Done: E(RM06) = -1196.84158743 Hartree

0 1

C	0.13967000	-2.51062700	-1.00518600
C	-1.22040600	-2.21370300	-1.64216000
H	0.04506400	-3.47768600	-0.49056300
H	0.92425100	-2.65803700	-1.75465300
C	-2.29112000	-1.71091500	-0.65262300
H	-1.11251000	-1.49516100	-2.46200900
H	-1.58221000	-3.14593900	-2.09693200
C	-2.28284500	-0.16022700	-0.57522500
H	-2.04495000	-2.10101200	0.34506600
H	-2.81932100	0.22392400	-1.45279300
C	-3.68559200	-2.22370600	-1.04631100
H	-3.92540800	-1.93449700	-2.07642700
H	-3.71596500	-3.31689300	-0.99052800
H	-4.46793700	-1.82051300	-0.40039500
C	-3.13763700	0.29251400	0.61906700
O	-4.34171600	0.42614400	0.57649000
O	-2.41349600	0.44172300	1.74813600
C	-3.16661200	0.85403200	2.89853900
H	-3.94308800	0.12212700	3.13605300
H	-2.44568800	0.92580500	3.71313200
H	-3.63940100	1.82242500	2.71680800
N	-0.94415800	0.41618000	-0.55132800
C	-0.91671600	1.76879900	-0.54084800
O	-1.89943100	2.51735100	-0.60862600
C	0.46170400	2.35414700	-0.39617900
C	0.68398700	3.72785800	-0.40967300
C	2.72902500	1.91907500	-0.05265000
C	1.98489900	4.19660700	-0.24124200
H	-0.17263500	4.37772100	-0.54778700
H	3.48196300	1.15055300	0.08415800
H	2.19038000	5.26284000	-0.24935500
C	3.02351500	3.27993800	-0.05890600
H	4.04831500	3.60763500	0.07752100
N	1.47125500	1.48326800	-0.21895500
Pd	0.86266800	-0.49583800	-0.33747100
O	2.80913700	-1.18806200	0.08210200
C	2.77470700	-2.20956400	0.84490900
O	1.70302800	-2.82216600	1.12836900
C	4.07452300	-2.71730300	1.42422200
H	4.90894700	-2.07542400	1.14253800
H	3.98902500	-2.76734500	2.51315000
H	4.25275900	-3.73526600	1.06548800
H	0.76924100	-2.33431700	0.23700200

^{M}1-δ

Thermal correction to Gibbs Free Energy= 0.404238 Hartree

SCF Done: E(RM06) = -1507.02403119 Hartree

0 1

C	-0.01898200	0.15215500	-2.31592200
C	1.16482400	-0.54206900	-2.96037800
H	0.03142500	1.23879200	-2.45868400
H	-0.96725600	-0.20648300	-2.72825400
C	2.54220500	-0.10251200	-2.43654600
H	1.07085800	-1.63343400	-2.87858300
H	1.13333900	-0.31831800	-4.03886500
C	2.89386000	-0.77895400	-1.08370100
H	2.50441100	0.98288300	-2.26352100
H	3.16299400	-1.82554500	-1.28350700
C	3.62536600	-0.39324100	-3.48982800
H	3.63727100	-1.45939600	-3.74632400
H	3.41864500	0.16743700	-4.40745300
H	4.62628200	-0.13046400	-3.14284800
C	4.19427400	-0.15716400	-0.54927900
O	5.30542600	-0.55914700	-0.81818400
O	3.96939000	0.96934600	0.16708500
C	5.14956700	1.58288000	0.70190100
H	5.83601700	1.87250500	-0.09836800
H	4.80593400	2.46223600	1.24793600
H	5.66501600	0.89217300	1.37420600
N	1.81256800	-0.72669900	-0.10188900
C	2.15934100	-1.22822300	1.10606100
O	3.26498000	-1.71004300	1.39685000
C	1.10598300	-1.15449100	2.18833000
C	1.39374100	-1.62462400	3.47022700
C	-1.03136800	-0.52278400	2.83006600
C	0.41569800	-1.52875000	4.45634600
H	2.37636700	-2.04669300	3.64528100
H	-1.97492700	-0.08167200	2.52222900
H	0.61175200	-1.88619500	5.46307200
C	-0.82091000	-0.96576900	4.13372500
H	-1.60984000	-0.87074400	4.87232000
N	-0.08765900	-0.61612000	1.88404800
Pd	-0.14989300	-0.12851500	-0.29374300
C	-2.36070700	-0.07122700	-0.43154200
C	-1.98116600	1.10265900	-0.23138700
C	-1.98722600	2.52440300	-0.02363300
C	-0.80085900	3.25004400	0.19391600
C	-3.21883500	3.21326400	-0.03546300
C	-0.84869900	4.62780600	0.39352800
H	0.14684100	2.72155100	0.20116200
C	-3.25527900	4.58943000	0.16549100
H	-4.13724000	2.66005600	-0.20546900
C	-2.07179400	5.30170700	0.38054100
H	0.07372000	5.17638000	0.55901500
H	-4.20923500	5.10848000	0.15266600
H	-2.10416200	6.37598500	0.53598100
C	-3.19337200	-1.21794500	-0.66541300
C	-4.59179600	-1.04603200	-0.74749600
C	-2.65525400	-2.50998000	-0.81464400
C	-5.42164800	-2.13988100	-0.97179800
H	-5.01378500	-0.05209900	-0.63608500
C	-3.49471100	-3.59838200	-1.04009400
H	-1.57998700	-2.64221300	-0.75449300
C	-4.87742900	-3.41913000	-1.11903100
H	-6.49614400	-1.99428700	-1.03396900
H	-3.06651300	-4.58965800	-1.15502900
H	-5.52839000	-4.27026700	-1.29522800

^{M}TS2-δ

Thermal correction to Gibbs Free Energy= 0.406774 Hartree

SCF Done: E(RM06) = 1506.97860613 Hartree

0 1

C	-0.17829500	-1.68057500	-1.28131600
C	0.74948300	-2.65763900	-0.56721300
H	0.32767800	-1.07448800	-2.04880100
H	-0.97377900	-2.18602700	-1.82411500
C	2.26109000	-2.34367500	-0.64553900
H	0.44683700	-2.79510500	0.47797900
H	0.60514100	-3.63995700	-1.03660600
C	2.74950500	-1.22392200	0.32117300
H	2.48253600	-2.00224900	-1.66587300
H	2.64980800	-1.59364600	1.35315200
C	3.03552000	-3.65215200	-0.39434800
H	2.81735600	-4.04784500	0.60464300
H	2.74325000	-4.41234300	-1.12616100
H	4.11630000	-3.51704000	-0.45842600
C	4.26784800	-1.05796900	0.12799600
O	5.11022300	-1.58345100	0.82239300
O	4.55455500	-0.37294000	-1.00340100
C	5.95008800	-0.12835300	-1.21698900
H	6.50760000	-1.06674400	-1.28107800
H	6.01566900	0.42057700	-2.15722800
H	6.35494700	0.46912600	-0.39603200
N	2.02504100	0.03196300	0.16049000
C	2.59995900	1.10489300	0.72485700

O	3.69489500	1.15743700	1.31271200
C	1.80726300	2.38234900	0.57370500
C	2.38348100	3.60620100	0.91391600
C	-0.16334600	3.43183900	-0.08266800
C	1.65226400	4.77489700	0.72953100
H	3.39219300	3.58747000	1.30989900
H	-1.17167300	3.31033900	-0.46203700
H	2.08177200	5.74049500	0.97995900
C	0.35694800	4.68751500	0.21438100
H	-0.24867300	5.57189000	0.04790000
N	0.54657000	2.30594800	0.09977900
Pd	-0.02954200	0.30512300	-0.30981300
C	-1.83156900	-1.03291400	-0.30347900
C	-2.06562300	0.23655100	-0.41187600
C	-3.17464500	1.17325300	-0.35462500
C	-4.17928800	1.14247700	-1.34136600
C	-3.27581900	2.12211100	0.68111500
C	-5.25191200	2.02996100	-1.28817400
H	-4.10670300	0.41892100	-2.14739500
C	-4.35684100	3.00074900	0.73322400
H	-2.51122500	2.14794200	1.45131700
C	-5.34660800	2.96175000	-0.25156500
H	-6.01809300	1.99171800	-2.05733600
H	-4.42700000	3.71647800	1.54748100
H	-6.18504700	3.65072300	-0.21131300
C	-2.44565500	-2.25485300	0.22137800
C	-3.30092100	-3.02841700	-0.58102400
C	-2.20183000	-2.65687500	1.54544100
C	-3.91071000	-4.17072400	-0.06394200
H	-3.49385900	-2.72287500	-1.60548100
C	-2.80873200	-3.80412600	2.05551000
H	-1.54313300	-2.05901800	2.16805300
C	-3.66377500	-4.56293200	1.25349600
H	-4.57836100	-4.75496000	-0.69054800
H	-2.61453900	-4.10379200	3.08117700
H	-4.13602600	-5.45548100	1.65318700

^{M}2-δ

Thermal correction to Gibbs Free Energy= 0.410243 Hartree

SCF Done: E(RM06) = -1507.01022718 Hartree

0 1

C	0.85211900	-2.37314800	0.08295100
C	-0.36708600	-2.17769100	-0.87537500
H	0.46744500	-2.40594500	1.11149900
H	1.29795500	-3.35537100	-0.10107800
C	-1.79294800	-2.41621500	-0.27253400
H	-0.26640600	-1.22981100	-1.51583100
H	-0.24096900	-2.84312000	-1.74110600
C	-2.87990800	-1.41992800	-0.80611400
H	-1.72771900	-2.21612600	0.80325400
H	-3.03077400	-1.58610700	-1.88258600
C	-2.20107100	-3.88383800	-0.47223600
H	-2.35609600	-4.10851200	-1.53377700
H	-1.42191600	-4.55505700	-0.09596000
H	-3.12772100	-4.12295900	0.05594400
C	-4.23623200	-1.73607600	-0.16417300
O	-5.18156200	-2.20144800	-0.76014400
O	-4.21708500	-1.50857300	1.16566800
C	-5.48616200	-1.64769700	1.82234300
H	-5.89494200	-2.65012400	1.67291000
H	-5.29424700	-1.46399800	2.87968500
H	-6.18711500	-0.90901600	1.42637400
N	-2.41796900	-0.06280000	-0.54932900
C	-3.19683700	0.98920200	-0.28505600
O	-4.43228700	1.07238000	-0.27921500
C	-2.35671300	2.20903800	0.08747700
C	-2.98278000	3.41331800	0.39407700
C	-0.25309200	3.14525600	0.50343200
C	-2.21075000	4.51083700	0.76709100
H	-4.06501900	3.43721000	0.33060500
H	0.81864800	2.98543900	0.52700500
H	-2.68165300	5.45864800	1.01066600
C	-0.82370100	4.37412200	0.82580200
H	-0.18298500	5.19986800	1.11508200
N	-1.00396200	2.09321200	0.14298200
Pd	-0.38830200	0.18435800	-0.44379400
C	1.95144000	-1.29786300	-0.00629800
C	1.62384800	0.00630600	-0.20791200
C	2.53429300	1.16149100	-0.19141700
C	3.23817300	1.51999400	0.97626100
C	2.66029400	1.99719700	-1.32012300
C	4.04579700	2.65683300	1.00670300
H	3.14907300	0.89114600	1.85685900
C	3.47586000	3.12763200	-1.29054800
H	2.11057700	1.74613900	-2.22327700
C	4.17178800	3.46465800	-0.12623600
H	4.58083500	2.91074600	1.91785300
H	3.56588800	3.74907800	-2.17744400

H	4.80299300	4.34825300	-0.10166300
C	3.34696000	-1.78883300	0.19895100
C	3.62894800	-2.74467700	1.19296500
C	4.41125300	-1.34797700	-0.60772200
C	4.92375100	-3.22712800	1.38411500
H	2.82985800	-3.10343900	1.83571400
C	5.70430400	-1.83319300	-0.42006400
H	4.21657500	-0.62649300	-1.39373000
C	5.96877100	-2.77367500	0.57794100
H	5.11491900	-3.95798900	2.16522500
H	6.50702900	-1.48044600	-1.06183900
H	6.97671800	-3.15191600	0.72230900

^{M}TS1-γ

Thermal correction to Gibbs Free Energy= 0.282847 Hartree

SCF Done: E(RM06) = -1196.84869384 Hartree

0 1

C	-0.80532200	-3.88215500	1.15008700
C	-1.40664100	-2.50025900	0.86581000
H	-1.01318000	-4.18450700	2.18172800
H	-1.23567400	-4.64660200	0.49161100
H	0.28065700	-3.89589100	1.01957200
C	-1.30527700	-2.06670800	-0.61154100
H	-2.46315300	-2.51281900	1.15499100
H	-0.92347400	-1.74567500	1.49833700
C	-1.91752600	-0.66580600	-0.89553500
H	-1.88891500	-2.78780800	-1.20258200
H	-2.08849200	-0.58693100	-1.97834700
C	0.14410600	-2.08029200	-1.14334800
H	0.47904800	-3.12041900	-1.24815300
H	0.20938400	-1.67513400	-2.15966600
C	-3.31535300	-0.51670800	-0.28810800
O	-4.31974900	-0.87312800	-0.86543200
O	-3.30346900	-0.02139200	0.96356800
C	-4.59934200	0.18374200	1.54584800
H	-5.15844100	-0.75429600	1.59228900
H	-4.41416200	0.57042100	2.54808000
H	-5.16690000	0.90722200	0.95546000
N	-0.95867000	0.33935200	-0.46649200
C	-1.25757700	1.65495000	-0.49495900
O	-2.34303000	2.16541200	-0.78584800
C	-0.07883700	2.51231900	-0.09087100
C	-0.19099800	3.88938300	0.06733700
C	2.19360600	2.56755000	0.45102000
C	0.93784700	4.61650800	0.44096000
H	-1.15969400	4.34237400	-0.11167500
H	3.10044000	1.98492500	0.57359700
H	0.87868000	5.69234800	0.57579800
C	2.14795500	3.94733400	0.63711200
H	3.04736600	4.47990800	0.92626300
N	1.09846500	1.87908200	0.09965600
Pd	0.96390500	-0.15423900	-0.31513500
C	3.30846300	-1.71698400	0.17114800
O	3.01850300	-0.49514100	-0.02582500
O	2.45641300	-2.65382900	0.09458600
H	1.30920000	-2.13345800	-0.35310200
C	4.73868000	-2.07518700	0.49921900
H	5.35066700	-1.18071900	0.61305600
H	4.76302300	-2.66922500	1.41659000
H	5.14338200	-2.69923000	-0.30324000

^{M}1-γ

Thermal correction to Gibbs Free Energy= 0.402538 Hartree

SCF Done: E(RM06) = -1507.01955792 Hartree

0 1

C	1.36560200	3.41555400	-2.19433900
C	2.11043400	2.24224500	-1.54684800
H	1.74620600	4.36939200	-1.81382300
H	1.49725500	3.41950000	-3.28302000
H	0.29127300	3.39025700	-1.98821900
C	1.77196500	0.86394600	-2.15312000
H	3.18685600	2.41452900	-1.65644500
H	1.91499400	2.22357300	-0.46827100
C	2.53812000	-0.30866400	-1.47600400
H	2.07293000	0.89508800	-3.21263000
H	2.50406500	-1.17437800	-2.15238900
C	0.27328300	0.51224500	-2.08037200
H	-0.38292900	1.33299600	-2.37583100
H	0.04257100	-0.36179800	-2.70187300
C	4.03244200	-0.02847100	-1.31143200
O	4.84970100	-0.27585100	-2.17259800
O	4.33561100	0.58387100	-0.14861900
C	5.73553100	0.80534200	0.06972500
H	6.16126000	1.42739300	-0.72208000
H	5.80832400	1.30900800	1.03401100
H	6.26854500	-0.14840800	0.09626600
N	1.82221300	-0.61023700	-0.23859200
C	2.30105900	-1.52808100	0.62464000

O	3.37870900	-2.12951400	0.54320900
C	1.37899000	-1.78949200	1.80498400
C	1.81349300	-2.59767900	2.85476700
C	-0.67978700	-1.46535800	2.82542000
C	0.95627700	-2.82853700	3.92809000
H	2.80955600	-3.02030500	2.78901600
H	-1.66052800	-1.00190400	2.76244900
H	1.27125100	-3.45045100	4.76101000
C	-0.31466000	-2.25155600	3.91689000
H	-1.01427700	-2.40650700	4.73148300
N	0.14679900	-1.23729700	1.79689900
Pd	-0.12825800	-0.14245600	-0.18042100
C	-2.32538500	-0.16923800	-0.43596100
C	-2.05242200	0.92109500	0.10960400
C	-2.18285000	2.22181300	0.70560300
C	-1.07567200	2.91799300	1.22548600
C	-3.45987100	2.81832400	0.77574700
C	-1.24379000	4.17615300	1.79913900
H	-0.09330800	2.46111700	1.16942100
C	-3.61719700	4.07523400	1.35123000
H	-4.31804700	2.28823500	0.37470800
C	-2.51126600	4.75878400	1.86490700
H	-0.38079800	4.70335100	2.19479100
H	-4.60518900	4.52393100	1.39797100
H	-2.63787700	5.74017100	2.31225700
C	-3.06143100	-1.24647900	-1.03827400
C	-4.45431500	-1.10831300	-1.21740400
C	-2.43600600	-2.43710900	-1.45302500
C	-5.19359400	-2.13565900	-1.79503000
H	-4.94322700	-0.19172700	-0.90256200
C	-3.18477300	-3.45966700	-2.03086300
H	-1.36487200	-2.54399500	-1.31732900
C	-4.56288900	-3.31429600	-2.20387400
H	-6.26481800	-2.01617300	-1.92857400
H	-2.68923300	-4.37250800	-2.34769300
H	-5.14307600	-4.11348600	-2.65545800

^{M}TS2-γ

Thermal correction to Gibbs Free Energy= 0.407000 Hartree

SCF Done: E(RM06) = 1506.96936607 Hartree

0 1

C	-0.01283500	-3.61334000	1.53177800
C	-1.09271100	-2.59004000	1.16394700
H	-0.02227600	-3.81003800	2.60897000
H	-0.18443500	-4.56934100	1.02245000
H	0.99278800	-3.27274500	1.27154900
C	-1.22236300	-2.33067700	-0.35026600
H	-2.05607200	-2.95228200	1.53571100
H	-0.90765100	-1.63992300	1.67832600
C	-2.47695700	-1.48862300	-0.73845800
H	-1.35198900	-3.31256500	-0.83269200
H	-2.60281100	-1.57229000	-1.82905200
C	0.06768700	-1.74435800	-1.01236400
H	0.77184800	-2.56229000	-1.10683300
H	-0.12503900	-1.36990700	-2.02132000
C	-3.76302600	-2.11726500	-0.18984200
O	-4.35442600	-3.00932300	-0.76095000
O	-4.13716400	-1.61458000	1.00155600
C	-5.38297800	-2.11638900	1.50719400
H	-5.34588900	-3.20200200	1.62985400
H	-5.52946800	-1.62541000	2.46937000
H	-6.19586600	-1.86373200	0.82202200
N	-2.23599800	-0.11934000	-0.35577400
C	-3.14011400	0.84801800	-0.54100100
O	-4.32218100	0.75099700	-0.89808600
C	-2.53831000	2.21432400	-0.25548900
C	-3.34268600	3.35087200	-0.24466700
C	-0.63514200	3.49501600	0.16241200
C	-2.76291000	4.59444500	-0.01002700
H	-4.40148100	3.21084400	-0.43190400
H	0.43992400	3.50201000	0.30625200
H	-3.37164100	5.49370700	0.00706100
C	-1.38442800	4.66815200	0.19612000
H	-0.88733400	5.61541200	0.37541600
N	-1.20005200	2.29599200	-0.05156100
Pd	-0.25958100	0.41184300	-0.23077900
C	1.74602900	-0.77420200	-0.34700700
C	1.74060100	0.51199500	-0.13320400
C	2.74600300	1.54515300	0.08291000
C	2.67513200	2.39216000	1.20552900
C	3.80246800	1.72426700	-0.83043200
C	3.64012800	3.37713200	1.41425800
H	1.86870700	2.25427200	1.92028200
C	4.75621700	2.71874300	-0.62336500
H	3.86443800	1.07778900	-1.70013700
C	4.68103000	3.54785200	0.49885700
H	3.57843100	4.01241600	2.29347800
H	5.56386600	2.84445100	-1.33901700

H	5.42773600	4.32014000	0.65830500
C	2.79588600	-1.80227200	-0.24777500
C	3.50148900	-1.94198000	0.96080500
C	3.17958900	-2.59120100	-1.34731100
C	4.55587800	-2.84853500	1.06614400
H	3.21222000	-1.33561000	1.81300700
C	4.23417700	-3.49630400	-1.23720700
H	2.66466600	-2.47760600	-2.29672200
C	4.92352000	-3.63076700	-0.03011200
H	5.08732100	-2.94591400	2.00842300
H	4.52243200	-4.09171000	-2.09869900
H	5.74259400	-4.33883400	0.05434800

M2-γ

Thermal correction to Gibbs Free Energy= 0.411742 Hartree

SCF Done: E(RM06) = -1507.03913396 Hartree

0 1

C	1.35345300	-3.16245400	2.23485200
C	0.17576800	-2.44796300	1.56086700
H	1.27848200	-3.08873500	3.32503300
H	1.37517900	-4.22841900	1.97764400
H	2.31693700	-2.73085600	1.94420700
C	0.15186700	-2.61060700	0.02851700
H	-0.76325500	-2.84140000	1.96301900
H	0.19349500	-1.38573100	1.82237000
C	-1.16683000	-2.12827400	-0.64975500
H	0.12410400	-3.69110000	-0.16868200
H	-1.04949100	-2.29876100	-1.72915300
C	1.45745700	-2.16235900	-0.68618000
H	2.19244200	-2.95743800	-0.52557600
H	1.26487100	-2.16080500	-1.76865000
C	-2.26457000	-3.14200300	-0.26698100
O	-2.38634400	-4.21288900	-0.82175600
O	-2.99303300	-2.77682900	0.81023900
C	-4.05584200	-3.67863400	1.14872300
H	-3.66844500	-4.67978900	1.35432000
H	-4.52794100	-3.25938700	2.03778700
H	-4.77372400	-3.73791300	0.32661100
N	-1.56302000	-0.74735700	-0.40977400
C	-2.77821700	-0.41953000	-0.89769100
O	-3.54101200	-1.17164300	-1.52396100
C	-3.24301400	0.98264300	-0.57709600
C	-4.53096600	1.38981900	-0.92403500
C	-2.78062300	3.04727500	0.37930600
C	-4.94282600	2.67694700	-0.59062400
H	-5.16178300	0.67851600	-1.44418300
H	-2.04638200	3.66791700	0.88398900
H	-5.94128800	3.01970700	-0.84584900
C	-4.05355900	3.52280700	0.07608600
H	-4.33402300	4.53297600	0.35477300
N	-2.38846900	1.80425000	0.06231000
Pd	-0.42829400	0.86843900	0.16825200
C	2.13592100	-0.85196000	-0.30647200
C	1.45445300	0.24767000	0.03081100
C	1.74459700	1.66203000	0.19154700
C	1.21097500	2.31576600	1.34213500
C	2.25682100	2.47385100	-0.86300600
C	1.20712700	3.72485100	1.42450800
H	0.94442300	1.72591700	2.21514000
C	2.26654300	3.84992700	-0.74963400
H	2.64900600	1.98395800	-1.74812900
C	1.72955700	4.48387100	0.39131300
H	0.82180300	4.20508100	2.31946600
H	2.68300300	4.45314700	-1.55137200
H	1.73999100	5.56764400	0.46032900
C	3.62932800	-0.83159400	-0.36684300
C	4.39500600	-0.12726400	0.57959100
C	4.31412600	-1.53036200	-1.37866900
C	5.78691700	-0.10758400	0.50853600
H	3.89065500	0.39089700	1.38880900
C	5.70657900	-1.50629000	-1.45254600
H	3.75277700	-2.08383300	-2.12502200
C	6.45057400	-0.79492600	-0.50975600
H	6.35480300	0.43670100	1.25814100
H	6.21038100	-2.04543500	-2.25007500
H	7.53532200	-0.78235100	-0.56346400

MTS1-γ′

Thermal correction to Gibbs Free Energy= 0.405778 Hartree

SCF Done: E(RM06) = -1506.97285077 Hartree

0 1

C	0.21147300	-2.39487400	-2.04779300
C	0.00831400	-2.31215700	-0.52664700
H	1.26440800	-2.58355200	-2.28023300
H	-0.36769700	-3.22098400	-2.47943200
H	-0.08128900	-1.47709600	-2.56458800
C	-1.44353100	-2.06283900	-0.03065600
H	0.24935300	-3.31344500	-0.13723200

C	-2.01012000	-0.72042900	-0.55372600
H	-1.39383900	-1.97111100	1.06133400
H	-2.29351800	-0.81950400	-1.61021600
C	-2.36799500	-3.24516500	-0.36954400
H	-2.46540000	-3.38421000	-1.45104200
H	-1.97394100	-4.17366700	0.05697700
H	-3.37465900	-3.09026600	0.02763700
C	-3.31662900	-0.37854900	0.16949200
O	-4.41708100	-0.61271100	-0.28034800
O	-3.09955600	0.11980100	1.40353400
C	-4.28467500	0.48903500	2.12417100
H	-4.94407700	-0.37279800	2.25541100
H	-3.93998700	0.85912500	3.08991100
H	-4.82507400	1.27075400	1.58465700
N	-0.97176800	0.27986100	-0.38860900
C	-1.23686700	1.59320200	-0.53076300
O	-2.33195600	2.10440800	-0.78630700
C	-0.00954900	2.44964000	-0.32024700
C	-0.06343100	3.83788100	-0.38207400
C	2.28249800	2.49047300	0.12881300
C	1.10753600	4.56488000	-0.17490800
H	-1.02242600	4.29834900	-0.59172900
H	3.16885800	1.89491200	0.31940800
H	1.09522500	5.64991700	-0.21588500
C	2.29824800	3.88284400	0.08473200
H	3.22903800	4.41441100	0.24993800
N	1.14841400	1.80377700	-0.06865400
Pd	0.91884000	-0.26705400	-0.09410900
C	3.22641600	-1.77500600	0.66308000
O	2.96610200	-0.59593900	0.26412400
O	2.34634000	-2.68178100	0.75049100
H	1.19395900	-2.17883500	0.23644300
C	4.64763300	-2.10940600	1.05071200
H	4.67154600	-2.40642400	2.10328600
H	4.99064800	-2.96590700	0.46394200
H	5.30900100	-1.25804800	0.89149800

^{M}1-γ′

Thermal correction to Gibbs Free Energy= 0.403225 Hartree

SCF Done: E(RM06) = -1507.02452645 Hartree

0 1

C	-0.11057900	0.02698900	-3.12468000
C	0.36896300	0.88480800	-1.95507000
H	-1.19096700	-0.13660900	-3.07871700
H	0.09855700	0.52119400	-4.08421600
H	0.37559100	-0.95389500	-3.14993700
C	1.89999900	1.12585100	-1.88538500
H	-0.14964800	1.85016100	-1.96951700
C	2.61394800	-0.16581200	-1.41169000
H	2.06383600	1.86470900	-1.09082800
H	2.68443600	-0.88757000	-2.23676900
C	2.48372300	1.69531900	-3.18940700
H	2.40929600	0.97814900	-4.01282200
H	1.95144800	2.60610600	-3.48461700
H	3.54211800	1.94522200	-3.07112100
C	4.06478600	0.13026400	-1.03090500
O	5.01225900	-0.06882100	-1.76035100
O	4.16308400	0.74018700	0.17100700
C	5.50406200	1.00818900	0.60239000
H	6.02110200	1.65657600	-0.11001900
H	5.41139300	1.50014500	1.57114300
H	6.06227800	0.07371400	0.70053200
N	1.79959000	-0.69213100	-0.32332800
C	2.27079300	-1.65391500	0.49018900
O	3.39062900	-2.17937400	0.44103300
C	1.28697800	-2.07804300	1.56861200
C	1.67020900	-3.03110500	2.51169300
C	-0.81146900	-1.87554200	2.53606700
C	0.76300000	-3.40575800	3.49995800
H	2.66914300	-3.44495600	2.43453500
H	-1.78332800	-1.39070400	2.50185100
H	1.03639900	-4.14483100	4.24749800
C	-0.50266200	-2.81761700	3.51594200
H	-1.23976900	-3.07964500	4.26768700
N	0.06077200	-1.51412500	1.58662000
Pd	-0.10929500	-0.08139400	-0.19392400
C	-2.30892000	0.17108800	-0.27302900
C	-1.84933600	1.20381800	0.26021800
C	-1.75574700	2.50454700	0.86374600
C	-0.54009100	3.00690700	1.36482100
C	-2.91723900	3.29972900	0.96276700
C	-0.49081000	4.27047100	1.94888700
H	0.35337200	2.39591700	1.28726100
C	-2.85754000	4.56007300	1.54914000
H	-3.85715300	2.92027600	0.57427700
C	-1.64555000	5.05030200	2.04392500
H	0.45348400	4.64677300	2.33094400
H	-3.75835800	5.16287700	1.61848200

H	-1.60256200	6.03503700	2.49975200
C	-3.23814600	-0.79614700	-0.78989200
C	-4.61341300	-0.48091500	-0.81981800
C	-2.81773700	-2.05335200	-1.26344300
C	-5.53501400	-1.40111900	-1.30936400
H	-4.94533000	0.48700500	-0.45752700
C	-3.74815100	-2.96791300	-1.75162500
H	-1.76048600	-2.29617100	-1.24460900
C	-5.10709900	-2.64715000	-1.77691400
H	-6.59045600	-1.14541700	-1.32760200
H	-3.40961000	-3.93366800	-2.11495600
H	-5.82926400	-3.36219800	-2.15957900

^{M}TS2-γ'

Thermal correction to Gibbs Free Energy= 0.405778 Hartree

SCF Done: E(RM06) = -1506.97285077 Hartree

0 1

C	-0.16387300	-0.98240600	-2.76922800
C	0.09737300	-1.55126300	-1.36792600
H	0.74126500	-0.51769600	-3.16858100
H	-0.43274200	-1.80957600	-3.44002000
H	-0.96831500	-0.24600300	-2.80256200
C	-1.10811500	-2.22926900	-0.62973900
H	0.81123200	-2.36048200	-1.50492400
C	-2.44165700	-1.41652400	-0.66756800
H	-0.82516700	-2.32337200	0.42482900
H	-2.88770300	-1.50500500	-1.66894400
C	-1.30655000	-3.64980200	-1.19688600
H	-1.51753100	-3.62511300	-2.27154700
H	-0.40984500	-4.25721400	-1.03883100
H	-2.14931600	-4.15180700	-0.71599000
C	-3.47237600	-2.07106100	0.26134700
O	-4.31311900	-2.86425700	-0.10563700
O	-3.29094800	-1.71821600	1.55003700
C	-4.25242300	-2.26026900	2.46711100
H	-4.23332500	-3.35330100	2.45034800
H	-3.96626600	-1.88638800	3.45039400
H	-5.25721200	-1.91972500	2.20540700
N	-2.15849200	-0.03864300	-0.34440900
C	-3.11928400	0.87711300	-0.20418900
O	-4.34877700	0.72166300	-0.23631300
C	-2.52704900	2.26219600	0.00082400
C	-3.36142000	3.36086900	0.19188300
C	-0.63475400	3.62049900	0.08996400
C	-2.80024200	4.62648400	0.33720400
H	-4.42950400	3.17457800	0.21095500
H	0.44757600	3.67101800	0.03871500
H	-3.43189000	5.49715500	0.48704300
C	-1.41183800	4.76002900	0.28090600
H	-0.93025000	5.72686300	0.38044500
N	-1.17882500	2.39998700	-0.04090700
Pd	-0.19543800	0.54027800	-0.32633100
C	1.62337800	-0.77533500	-0.38343200
C	1.81379200	0.52119000	-0.26936400
C	2.93600100	1.40354800	-0.00677100
C	2.83670200	2.42508000	0.95869200
C	4.14434500	1.26935200	-0.71951200
C	3.91345400	3.27226400	1.21184500
H	1.91474500	2.52504200	1.52368500
C	5.21295600	2.12794300	-0.47274500
H	4.23026000	0.48943000	-1.46950500
C	5.10352500	3.13149200	0.49348900
H	3.82445300	4.04444500	1.97092800
H	6.13600700	2.01197600	-1.03382000
H	5.93921700	3.79815500	0.68502800
C	2.37766800	-1.93379100	0.13828000
C	2.29195200	-2.27259100	1.49840500
C	3.20295500	-2.70177500	-0.70057200
C	3.01914900	-3.34968400	2.00619600
H	1.65414400	-1.68357000	2.15037700
C	3.93473400	-3.77287700	-0.18805700
H	3.27838300	-2.44696900	-1.75435300
C	3.84251500	-4.10153500	1.16601700
H	2.94322200	-3.59943000	3.06062900
H	4.57769500	-4.34981000	-0.84667100
H	4.40946100	-4.93820300	1.56364600

^{M}2-γ'

Thermal correction to Gibbs Free Energy= 0.412264 Hartree

SCF Done: E(RM06) = -1507.03769262 Hartree

0 1

C	1.11562900	-1.89811300	-2.19949600
C	1.48424300	-2.02382500	-0.70504100
H	1.99314300	-1.62445600	-2.79376400
H	0.73595300	-2.84794400	-2.59119500
H	0.35427300	-1.13028000	-2.35631200
C	0.30087600	-2.50701900	0.21855600
H	2.23955500	-2.81345200	-0.64077400

C	-1.12230700	-2.27004400	-0.36089100
H	0.35076700	-1.93629600	1.15182500
H	-1.18224100	-2.70537500	-1.36473700
C	0.53514900	-3.99244000	0.56311600
H	0.45459900	-4.62859900	-0.32524900
H	1.53898200	-4.11755200	0.98100600
H	-0.17842400	-4.37211500	1.29689300
C	-2.15029400	-3.10086600	0.43000600
O	-2.48825400	-4.22463400	0.13132500
O	-2.56851600	-2.47355000	1.55340600
C	-3.55138800	-3.19286600	2.31092300
H	-3.15838100	-4.15703000	2.64468900
H	-3.78818100	-2.55975100	3.16664700
H	-4.44362800	-3.36831100	1.70443800
N	-1.50419400	-0.85535400	-0.41348400
C	-2.77508000	-0.64120800	-0.81268400
O	-3.56633100	-1.51019500	-1.21092300
C	-3.26849900	0.78202300	-0.69104500
C	-4.55290300	1.11596800	-1.11991800
C	-2.88017000	2.95221000	0.03717800
C	-4.99812800	2.42468500	-0.95742600
H	-5.15695300	0.33176400	-1.56116900
H	-2.17853400	3.64154800	0.49746100
H	-5.99310700	2.71299000	-1.28366200
C	-4.14812900	3.36205900	-0.36601700
H	-4.45634600	4.39149000	-0.21742800
N	-2.45376300	1.69001300	-0.12129100
Pd	-0.47438900	0.83692700	0.15622900
C	2.17178600	-0.75085000	-0.20474600
C	1.44331900	0.31444100	0.15087900
C	1.65849800	1.73450800	0.35641000
C	0.99749800	2.35148300	1.46029100
C	2.22708200	2.58071200	-0.64136600
C	0.92531200	3.75790200	1.55342200
H	0.67841000	1.74011600	2.30021000
C	2.16778500	3.95443500	-0.51622700
H	2.71622200	2.11814200	-1.49227100
C	1.50450800	4.55104200	0.57745200
H	0.44094700	4.21028400	2.41405500
H	2.62698300	4.58434500	-1.27284000
H	1.46174200	5.63338400	0.65626400
C	3.66234100	-0.72638500	-0.13795300
C	4.33319300	0.01736500	0.85199600
C	4.44990500	-1.45852900	-1.04731300
C	5.72442100	0.04821300	0.91555600
H	3.75270100	0.55617600	1.59338800
C	5.84288400	-1.42525500	-0.98544100
H	3.97444200	-2.04923100	-1.82277700
C	6.48908700	-0.67057400	-0.00576700
H	6.21243200	0.62464000	1.69675400
H	6.42355700	-1.99276200	-1.70745900
H	7.57379600	-0.65039900	0.04568900

2-Ni

Thermal correction to Gibbs Free Energy= 0.431324 Hartree

SCF Done: E(RM06) = -2053.84402323 Hartree

0 1

Pd	-2.55045200	-0.76631400	0.63674100
O	-1.03283900	-0.26248200	-2.14898700
C	-2.19415900	-0.78245900	-2.28798600
O	-3.00198400	-1.13013800	-1.37755900
C	-2.63547800	-1.01301500	-3.71905600
H	-2.03291100	-1.81872100	-4.15032900
H	-3.68965600	-1.28650500	-3.75537300
H	-2.45197100	-0.11317500	-4.31125900
C	-4.74176700	0.91701200	0.96985100
O	-3.50205200	1.00210900	0.51943100
O	-5.24443200	-0.10430400	1.41747900
C	-5.49367100	2.23699700	0.87301000
H	-6.49558100	2.11788800	1.28651700
H	-4.95788800	3.01865300	1.42026100
H	-5.56325000	2.55277900	-0.17278100
C	-0.38278300	0.08446400	2.40610800
O	0.37709600	0.15043600	1.37133700
O	-1.60868400	-0.20689700	2.42883300
C	-0.51800900	-2.80442300	0.25543500
O	-1.63336800	-2.60226100	0.81414000
O	0.13857900	-1.93402800	-0.40145500
C	0.08471600	-4.18667800	0.35123500
H	-0.12848000	-4.73068500	-0.57549800
H	1.16783700	-4.10382900	0.46162300
H	-0.35656600	-4.73471400	1.18405100
C	0.28788200	0.37981300	3.73120800
H	-0.44411500	0.37565900	4.53807400
H	1.05100000	-0.38105200	3.92211800
H	0.79213500	1.34893400	3.68330200
C	1.11712200	2.44272400	-1.08969300
C	1.17138000	3.79500400	-1.43436500

C	0.01608600	4.56692200	-1.37518300
C	-1.17494100	3.95749800	-0.98851000
C	-1.17497400	2.59874400	-0.68806300
H	2.12435500	4.20017200	-1.75145700
H	0.04563200	5.62192500	-1.63007200
H	-2.10444600	4.51236000	-0.92509200
H	-2.08785900	2.08750100	-0.39466500
N	-0.04965900	1.85688100	-0.73436900
C	2.42637700	1.67734200	-1.19268300
O	3.21510600	1.99108400	-2.07859600
C	3.89983100	-0.06893100	-0.26901500
C	4.71086800	0.03991300	1.04172800
H	4.48417000	0.31193200	-1.11042700
C	4.99449800	1.51338900	1.39802800
H	4.10109700	-0.40568300	1.84020900
C	5.63926200	1.71632800	2.77427000
H	5.63526500	1.95252700	0.62093200
H	4.05443500	2.07578500	1.36830500
H	6.63732800	1.27221200	2.83501400
C	3.49228900	-1.51854400	-0.58403300
O	3.30863400	-2.38893900	0.24177900
O	3.33586400	-1.67957900	-1.90756300
C	6.00726800	-0.77813800	0.91859400
H	6.64805900	-0.37596100	0.12419500
H	5.79803700	-1.82716300	0.69742100
H	6.57775800	-0.75090700	1.85045200
C	2.83953400	-2.96107300	-2.33702300
H	2.90222600	-2.94794600	-3.42482900
H	1.80475700	-3.07804200	-2.01055400
H	3.45189900	-3.76627400	-1.92430800
H	5.74362300	2.78283100	2.99832900
H	5.02829700	1.26929900	3.56722900
N	2.66885800	0.72604200	-0.25145300
H	2.01996700	0.58670200	0.52084200
Ni	-0.20757500	-0.07692300	-0.45447900

TS1-NI

Thermal correction to Gibbs Free Energy= 0.429869 Hartree

SCF Done: E(RM06) = -2053.83896525 Hartree

0 1

Pd	-1.71104000	-0.86997200	0.93227500
O	-1.03756200	-1.23387200	-2.18178600
C	-1.98741300	-1.98109900	-1.77470100
O	-2.44120900	-2.04068900	-0.59897400
C	-2.62102400	-2.89120200	-2.80581900
H	-3.60243100	-3.22520700	-2.46909000
H	-2.69322300	-2.37943600	-3.76776100
H	-1.97587300	-3.76563900	-2.94228800
C	-4.20997200	0.40626200	1.10830400
O	-3.05366500	0.56362800	0.49193300
O	-4.48672000	-0.50953400	1.86942900
C	-5.19801500	1.51999400	0.77260700
H	-6.15315900	1.32066900	1.25959800
H	-4.80542500	2.48314600	1.11355700
H	-5.34223100	1.58924100	-0.31009100
C	-0.41731100	1.22707300	2.67535500
O	0.37249500	1.69655600	1.78651600
O	-1.14759400	0.20468600	2.59174200
C	0.64840100	-2.57975000	0.47814300
O	-0.29128800	-2.34789600	1.28769000
O	0.97947700	-1.85816400	-0.52437100
C	1.46346800	-3.83637400	0.68545700
H	1.08606400	-4.60990800	0.00724000
H	2.50955700	-3.64631400	0.43888700
H	1.35768400	-4.19145300	1.71069300
C	-0.45997400	1.98899800	3.98364500
H	-0.64041600	3.04784300	3.77874100
H	-1.23514400	1.59242000	4.63821400
H	0.51572900	1.91325400	4.47365500
C	0.18815200	2.48595900	-1.47553400
C	-0.19564600	3.77432600	-1.82680000
C	-1.47975400	3.96326300	-2.33472000
C	-2.31982400	2.85912700	-2.47525900
C	-1.85626200	1.59453000	-2.11827300
H	0.51268900	4.58360700	-1.69486500
H	-1.82166900	4.95511300	-2.61332100
H	-3.32998400	2.96524800	-2.85441600
H	-2.46926700	0.71058300	-2.20406300
N	-0.61739300	1.41950100	-1.63469500
C	1.53775600	2.16651100	-0.92666700
O	2.47365200	2.93512000	-1.05802000
C	2.97130700	0.48776900	0.04409900
C	3.31880400	0.44430400	1.55451900
H	3.59006300	1.28628900	-0.37854000
C	4.84705400	0.51630800	1.79185800
H	2.88924200	1.36954900	1.96001300
C	5.52567700	1.81573500	1.34000000
H	5.00743100	0.40002100	2.87119400

H	5.32647400	-0.34662400	1.31846500
H	5.04710900	2.69328000	1.78966600
C	3.38205000	-0.73930400	-0.76939800
O	4.04958200	-1.66946000	-0.36886400
O	3.01178700	-0.59270300	-2.06077600
C	2.71504300	-0.73240200	2.33341000
H	3.15680100	-1.67853400	2.01290900
H	1.63110700	-0.79929200	2.22323300
H	2.92387200	-0.61162300	3.40197300
C	3.32739200	-1.69852800	-2.92409400
H	3.01615400	-1.39015800	-3.92186500
H	2.77219200	-2.58360800	-2.60575800
H	4.39859200	-1.91033100	-2.90100400
H	6.57880700	1.82171500	1.63975600
H	5.50251100	1.95207100	0.25371300
N	1.56402500	0.91673200	-0.25911100
H	0.88216100	1.15750800	0.79865900
Ni	0.17624800	-0.24888200	-1.09100300

3-Ni

Thermal correction to Gibbs Free Energy= 0.376604 Hartree

SCF Done: E(RM06) = -1824.79872316 Hartree

0 1

C	0.65151800	-2.79942300	-0.58690200
C	1.36041400	-3.99349300	-0.52577100
C	2.58204200	-4.07866000	-1.18948600
C	3.04843700	-2.96815200	-1.89604800
C	2.28301900	-1.80552800	-1.91421400
H	0.92232400	-4.81379400	0.03116200
H	3.16189200	-4.99640300	-1.16330300
H	3.99148200	-2.99397600	-2.43072300
H	2.58889900	-0.91714000	-2.45063300
N	1.10929600	-1.73037900	-1.26638800
C	-0.69749500	-2.62146400	0.04498400
O	-1.25561800	-3.57381400	0.60218600
N	-1.15842300	-1.36266700	-0.12328800
C	1.74581000	1.85291400	-2.15126400
O	2.08115500	2.34690000	-1.02843800
O	1.04434400	0.81771000	-2.33842100
C	2.22958500	2.59807300	-3.37772200
H	2.45536300	1.89331100	-4.18021600
H	3.10014600	3.21024900	-3.14130200
H	1.42295300	3.25232100	-3.72548400
C	-2.56327400	-1.14082900	0.21950500
C	-2.83806200	-0.38870300	1.55808700
H	-2.98758400	-2.14846200	0.34657400
C	-1.77496200	-0.73483800	2.62082000
H	-2.78982700	0.69123200	1.37369900
C	-1.92576400	0.04062800	3.93466500
H	-1.80810400	-1.81481200	2.81606700
H	-0.78252000	-0.52662400	2.21355900
H	-2.84558500	-0.21309700	4.47146500
C	-3.25941600	-0.59503800	-1.03711500
O	-3.00480100	-0.97213400	-2.16065500
O	-4.23614800	0.30704000	-0.77942700
C	-4.25303500	-0.72639300	2.06401600
H	-4.31043200	-1.78196000	2.35737900
H	-5.01171000	-0.54106200	1.30151000
H	-4.51204100	-0.12487700	2.93999600
C	-4.94680000	0.77346000	-1.93834600
H	-5.71179300	1.45263100	-1.56089000
H	-5.40480200	-0.06273800	-2.47193000
H	-4.26820900	1.29468300	-2.61798900
H	-1.08516900	-0.17403800	4.60224000
C	-1.12694900	2.26632700	-0.09116300
O	-0.16780300	2.52972300	0.70220100
O	-1.21383800	1.28447500	-0.88457400
C	-2.27670400	3.25148800	-0.09533100
H	-2.21445200	3.85596400	-1.00647000
H	-2.22569800	3.90881800	0.77241500
H	-3.22104700	2.70435400	-0.11610700
C	2.74093500	0.09384700	2.40213500
O	3.27713900	0.38064800	1.27500200
C	3.46203000	-0.72467300	3.42730600
H	2.76269200	-1.40798900	3.91426600
H	3.87081400	-0.05758000	4.19368100
H	4.28142200	-1.27552800	2.96321900
O	1.56917700	0.55483300	2.62572600
H	-1.92972100	1.12252300	3.75523100
Ni	-0.04084900	-0.19596900	-1.10925900
Pd	1.57916400	1.48700300	0.75395300

TS2-Ni

Thermal correction to Gibbs Free Energy= 0.376651 Hartree

SCF Done: E(RM06) = -1824.77810882 Hartree

0 1

C	1.09412700	-2.57806700	-0.63380200
C	1.98352100	-3.64573900	-0.62406700

C	3.18793300	-3.51510200	-1.30932100
C	3.45671400	-2.32359100	-1.98716700
C	2.51664700	-1.29810000	-1.95391300
H	1.69582100	-4.53895600	-0.08201800
H	3.90724200	-4.32844300	-1.32187400
H	4.37940100	-2.18377700	-2.53963200
H	2.66300900	-0.35760400	-2.46852100
N	1.36130500	-1.43088500	-1.28236600
C	-0.24386300	-2.63220400	0.03588200
O	-0.61403900	-3.65854900	0.61456800
N	-0.91991200	-1.46691500	-0.10555400
C	1.53474600	2.22930000	-1.82173600
O	1.66965400	2.68855200	-0.64345300
O	0.95767300	1.15672400	-2.16429000
C	2.17459100	3.05411800	-2.92257300
H	3.17203500	2.65035500	-3.12812100
H	2.28204600	4.09344300	-2.61167700
H	1.58122400	2.98035200	-3.83550700
C	-2.29522500	-1.46335100	0.39333800
C	-2.50921600	-0.64456500	1.70170100
H	-2.51183300	-2.51257600	0.64381100
C	-1.38174700	-0.91517500	2.74390800
H	-2.49066400	0.41987600	1.44783800
C	-0.68575700	0.34150300	3.26364300
H	-1.81707200	-1.43127700	3.60921300
H	-0.61960900	-1.58607900	2.34756400
H	-1.37581300	1.15061000	3.52053200
C	-3.21704800	-1.17385500	-0.80268800
O	-3.07545700	-1.69750800	-1.88517700
O	-4.24214000	-0.32634300	-0.53821800
C	-3.87435200	-0.95234600	2.34719800
H	-3.94168700	-2.01556800	2.60826700
H	-4.71108900	-0.70579000	1.69343900
H	-3.98752300	-0.38110300	3.27540600
C	-5.14279900	-0.11572600	-1.63921800
H	-5.92507400	0.54199600	-1.25888200
H	-5.56818500	-1.06426100	-1.97480200
H	-4.61898700	0.35041900	-2.47720500
H	-0.03398000	0.13057500	4.11532400
C	-1.70330800	2.07349700	-0.35059800
O	-0.93111100	2.48020300	0.57524900
O	-1.51927700	1.05712700	-1.08039000
C	-2.97233900	2.86348100	-0.57218400
H	-3.09723000	3.06113000	-1.63971400
H	-2.95211700	3.79735200	-0.01111100
H	-3.81612900	2.24907500	-0.24547400
C	2.89353200	-0.09396400	1.87575400
O	2.75957200	1.14270000	1.45464500
C	4.31264800	-0.42958800	2.29996800
H	4.38059800	-1.49053700	2.54347900
H	4.58530200	0.16549600	3.17713000
H	5.01866700	-0.17712700	1.50401800
O	1.96741700	-0.90137100	1.93492600
H	0.00689300	0.74118900	2.48074100
Ni	-0.03503000	-0.12313600	-1.10005300
Pd	0.89423300	1.65126500	0.97254500

4-Ni

Thermal correction to Gibbs Free Energy= 0.378329 Hartree

SCF Done: E(RM06) = -1824.78350196 Hartree

0 1

C	1.07572100	-2.50751700	-0.70163000
C	1.96525900	-3.57392500	-0.73993400
C	3.13370200	-3.43900900	-1.48348200
C	3.36879200	-2.24377300	-2.16727600
C	2.43106500	-1.21920400	-2.08308100
H	1.70739900	-4.46939600	-0.18695700
H	3.85228600	-4.25144600	-1.53482400
H	4.26399500	-2.10023000	-2.76240000
H	2.55322300	-0.27400200	-2.59598600
N	1.30861300	-1.35825100	-1.35946800
C	-0.23294300	-2.56878400	0.02295100
O	-0.58219000	-3.60012600	0.60696400
N	-0.92255100	-1.40711800	-0.09107900
C	1.49162800	2.33160100	-1.68602100
O	1.64065000	2.69991600	-0.47589400
O	0.91638000	1.28505700	-2.10411800
C	2.05356200	3.27712800	-2.72978100
H	2.78723000	3.95144500	-2.28843800
H	1.22880800	3.86860400	-3.14111600
H	2.49487100	2.70763900	-3.55030800
C	-2.30815200	-1.44588800	0.37520400
C	-2.60174100	-0.73029500	1.72873100
H	-2.51407100	-2.51182400	0.55572800
C	-1.48177500	-0.96608200	2.78016700
H	-2.70175100	0.34742500	1.56016800
C	-0.39685100	0.11852900	2.90702500
H	-1.95927800	-1.02187500	3.76676400

H	-1.01184600	-1.93999500	2.61290700
H	-0.83836400	1.11397200	2.99730100
C	-3.21140200	-1.09972800	-0.81959500
O	-3.03954500	-1.55025200	-1.93022200
O	-4.25949700	-0.29404100	-0.51738100
C	-3.93169000	-1.24918500	2.31323700
H	-3.83947700	-2.30678900	2.58852500
H	-4.75797700	-1.14940700	1.60834100
H	-4.19244200	-0.69354900	3.22031800
C	-5.14682500	-0.02838700	-1.61747200
H	-5.94854500	0.58695500	-1.20793400
H	-5.54695600	-0.96070800	-2.02242100
H	-4.61898800	0.50276000	-2.41312100
H	0.24955400	-0.05970200	3.77001600
C	-1.73144700	2.08635800	-0.20513800
O	-0.94670500	2.41537800	0.74000500
O	-1.55780800	1.13039700	-1.01453300
C	-2.99854900	2.89745600	-0.35224100
H	-2.97093900	3.78121900	0.28457500
H	-3.84420600	2.26219200	-0.07377300
H	-3.12809300	3.18461200	-1.39874100
C	3.07159000	-0.19897300	1.74199400
O	2.76773200	1.05591400	1.49716100
C	4.53737600	-0.36838000	2.11958800
H	5.18039200	0.05543400	1.34321700
H	4.75877600	-1.42736200	2.25626700
H	4.74607500	0.17470900	3.04622000
O	2.28945100	-1.14390100	1.69095200
H	0.28828800	-0.00927600	2.02347100
Ni	-0.07376000	-0.05152600	-1.10386200
Pd	0.88637400	1.54392400	1.04872200

TS3-Ni

Thermal correction to Gibbs Free Energy= 0.377580 Hartree

SCF Done: E(RM06) = -1824.76670116 Hartree

0 1

C	1.37235300	-2.34311800	-0.78544600
C	2.35541300	-3.32497100	-0.83972000
C	3.51994500	-3.06285000	-1.55760800
C	3.65907600	-1.82922500	-2.19915200
C	2.63342200	-0.89332100	-2.09858100
H	2.16545900	-4.25962200	-0.32480100
H	4.30641800	-3.80870300	-1.62503100
H	4.54622000	-1.58921400	-2.77492100
H	2.67615700	0.07664200	-2.57740500
N	1.51709500	-1.15332700	-1.39816500
C	0.06206900	-2.54376500	-0.08468400
O	-0.18934900	-3.61855900	0.47188800
N	-0.72307600	-1.44620000	-0.18140000
C	1.32582800	2.51375700	-1.59219500
O	1.36331700	2.81648400	-0.36375400
O	0.88217500	1.43515400	-2.10159800
C	1.84118600	3.56019300	-2.56164900
H	2.57249900	4.20627800	-2.07486400
H	0.99642900	4.17701400	-2.88707300
H	2.26909900	3.08506900	-3.44626300
C	-2.08958100	-1.59791500	0.31079300
C	-2.42579000	-0.86890600	1.64766600
H	-2.19619700	-2.67272800	0.52159200
C	-1.25783900	-0.91518000	2.67250100
H	-2.66738600	0.17752700	1.44323600
C	-0.37225900	0.34763400	2.80819100
H	-1.70522000	-1.05464600	3.66598700
H	-0.64546800	-1.80571200	2.50021000
H	-1.00444200	1.23670600	2.81122700
C	-3.03968500	-1.36363500	-0.87468000
O	-2.83938600	-1.80787800	-1.98303900
O	-4.16137800	-0.66787000	-0.56301600
C	-3.66633300	-1.52448300	2.28930700
H	-3.43732500	-2.54985900	2.60400500
H	-4.51332000	-1.55824900	1.60214300
H	-3.97636500	-0.96647100	3.17933900
C	-5.09020700	-0.51203200	-1.64942800
H	-5.94516100	0.02136600	-1.23261300
H	-5.39700600	-1.48671800	-2.03588200
H	-4.63519000	0.06055900	-2.46116200
H	0.15130800	0.35046000	3.77191600
C	-1.90517300	1.94923800	-0.27121800
O	-1.23573500	2.30036900	0.75004100
O	-1.59091400	1.04341700	-1.09727500
C	-3.22202300	2.65918300	-0.49634000
H	-3.28641800	3.55920100	0.11484500
H	-4.02818000	1.97152900	-0.22380500
H	-3.33314300	2.90410100	-1.55504000
C	2.78329900	-0.15216100	1.94613100
O	2.60215700	1.03448900	1.52140700
C	4.20494700	-0.63580900	2.10708400
H	4.38542400	-1.44122600	1.38870100

H	4.33394900	-1.05478900	3.10806000
H	4.91418000	0.17384800	1.93855100
O	1.83354700	-0.93905100	2.22796400
H	0.74728700	-0.15448800	2.21533400
Ni	0.00347500	0.00984900	-1.15115900
Pd	0.64355700	1.53858200	1.13296300

5-Ni

Thermal correction to Gibbs Free Energy= 0.380885 Hartree

SCF Done: E(RM06) = -1824.80147973 Hartree

0 1

C	1.43453100	-2.61392800	0.05213700
C	2.34264700	-3.64904100	0.24150700
C	3.48065500	-3.69117300	-0.56045500
C	3.66436000	-2.70199400	-1.52914400
C	2.70686400	-1.70064300	-1.66778200
H	2.11949800	-4.38746800	1.00284300
H	4.21111400	-4.48570700	-0.44073500
H	4.53193000	-2.70415800	-2.18001600
H	2.77403200	-0.92202800	-2.41681400
N	1.61977900	-1.65931200	-0.88015700
C	0.16107800	-2.49813400	0.84098300
O	-0.13519600	-3.36381500	1.66701500
N	-0.52479700	-1.36242700	0.52159800
C	1.49298500	1.61994700	-2.37611200
O	1.57986700	2.30381200	-1.33125400
O	0.94737300	0.46125300	-2.48996900
C	2.06419600	2.19935000	-3.66038800
H	2.93448000	2.81963800	-3.43843300
H	1.30329800	2.84122400	-4.11798000
H	2.31870500	1.41341700	-4.37364100
C	-1.91203000	-1.33272600	1.00241700
C	-2.38233800	-0.05193000	1.75474800
H	-1.98306100	-2.16231900	1.72120000
C	-1.28471600	0.58084000	2.65613000
H	-2.68942600	0.69652100	1.02148000
C	-0.76579700	1.95632200	2.22923700
H	-1.71501700	0.72172700	3.66028700
H	-0.46363200	-0.12683400	2.81162900
H	-1.59529100	2.64285200	2.02943900
C	-2.79632600	-1.79180700	-0.17540000
O	-2.47254800	-2.67135000	-0.94279600
O	-3.99468600	-1.16755300	-0.24930300
C	-3.61170500	-0.39122500	2.62259700
H	-3.33687800	-1.09153000	3.42109000
H	-4.41844100	-0.83702400	2.03889600
H	-4.00055900	0.51570800	3.09815800
C	-4.84721000	-1.62664100	-1.31207100
H	-5.77373200	-1.06045100	-1.21157000
H	-5.03903300	-2.69794100	-1.21645300
H	-4.38120400	-1.43714800	-2.28196500
H	-0.12372900	2.38824300	3.00648100
C	-1.92182200	1.57553200	-1.25904300
O	-1.36912400	2.44748500	-0.52869800
O	-1.53014300	0.38262400	-1.44013100
C	-3.20331200	1.97425000	-1.96219100
H	-3.39408900	3.04081900	-1.84503000
H	-4.02784000	1.40232500	-1.52598900
H	-3.13996200	1.71155700	-3.02113800
C	2.59919100	0.89609300	2.17864500
O	2.14058700	1.93903200	1.70534500
C	3.87918100	0.86911700	2.96229000
H	4.59165400	0.19612600	2.47640600
H	3.68613400	0.47338800	3.96369400
H	4.29493100	1.87324100	3.02692400
O	2.02667500	-0.28738400	2.05208300
H	1.18425900	-0.22338300	1.53756000
Ni	0.12613400	-0.44236100	-1.02536700
Pd	0.36500400	2.06198700	0.53710700

6-Ni

Thermal correction to Gibbs Free Energy= 0.497014 Hartree

SCF Done: E(RM06) = -2134.95035001 Hartree

0 1

C	-2.43402000	2.37551700	-0.14784600
C	-2.76391400	3.71133100	-0.34717300
C	-2.45491400	4.63065700	0.65306100
C	-1.82864800	4.18103400	1.81689000
C	-1.53318100	2.82651400	1.94853800
H	-3.25720500	3.97806700	-1.27487000
H	-2.70086500	5.68148000	0.53222100
H	-1.57279500	4.86343100	2.61996200
H	-1.06858200	2.40945000	2.83273200
N	-1.83058200	1.94880900	0.97737200
C	-2.74383900	1.30604300	-1.15462700
O	-3.26994300	1.60832700	-2.23356400
N	-2.37733400	0.08155700	-0.71060800
C	0.40029100	-0.04719800	3.00190300

O	1.29502400	-0.23680200	2.14862600
O	-0.85045600	0.13700800	2.77169200
C	0.79566800	-0.04825100	4.47030500
H	0.10203900	0.54648100	5.06751800
H	1.81793400	0.31577300	4.58623200
H	0.75964200	-1.07981600	4.83775600
C	-2.73695900	-1.03777200	-1.57966400
C	-1.55355900	-1.78146400	-2.27059700
H	-3.35593400	-0.59819600	-2.37545300
C	-0.37774700	-0.84567200	-2.66113000
H	-1.17443900	-2.52909700	-1.57226300
H	-0.23838200	-0.89751300	-3.75272200
H	-0.62974800	0.20056000	-2.46476700
C	-3.66011200	-2.01838700	-0.84655500
O	-3.59361500	-3.23144000	-0.90110000
O	-4.62646000	-1.36975400	-0.16777200
C	-2.06926700	-2.53611400	-3.50985700
H	-2.45041600	-1.83493900	-4.26274700
H	-2.86674200	-3.23397600	-3.24713800
H	-1.25818800	-3.11029600	-3.97146900
C	-5.53778000	-2.20955200	0.55584700
H	-6.26896900	-1.53493100	1.00131300
H	-5.00589500	-2.76295100	1.33398200
H	-6.02810400	-2.91949700	-0.11508600
C	-0.89339100	-2.73824900	0.97914800
O	0.22866800	-2.57049900	0.42888600
O	-1.75505400	-1.83048300	1.21520700
C	-1.30301000	-4.14516900	1.35878900
H	-0.46397800	-4.83326400	1.25546800
H	-2.11898300	-4.44616900	0.69442800
H	-1.68272900	-4.15757500	2.38365500
Pd	1.12001400	-0.67693400	-0.07513900
C	0.98005400	-1.19152200	-2.05197700
H	1.16781600	-2.27004500	-2.03640400
H	1.79292400	-0.68107800	-2.57288100
C	3.15269200	-0.00690300	-0.33426000
C	2.43434300	1.00555500	-0.52173400
C	4.29424300	-0.87611600	-0.25546700
C	4.18430700	-2.21977300	0.14783000
C	5.56376400	-0.35903800	-0.58907700
C	5.32000700	-3.02358900	0.21343100
H	3.20850300	-2.61725600	0.40752100
C	6.69148500	-1.17081300	-0.51611800
H	5.65138100	0.67540600	-0.90643300

C	6.57418800	-2.50483400	-0.11586200
H	5.22402400	-4.05943200	0.52499700
H	7.66393400	-0.76261000	-0.77564300
H	7.45634900	-3.13593700	-0.06205600
C	2.16576600	2.40141100	-0.75221600
C	3.14063600	3.35223700	-0.38393600
C	0.98234500	2.84373900	-1.36816100
C	2.93361600	4.70519900	-0.63574700
H	4.05125500	3.01637800	0.10205200
C	0.78709400	4.19943900	-1.62430200
H	0.23017900	2.11759100	-1.65358900
C	1.75865300	5.13397700	-1.25977600
H	3.69215600	5.42680300	-0.34629800
H	-0.12703700	4.52343100	-2.11217100
H	1.60317700	6.18982200	-1.46127200
Ni	-1.61627600	0.03292900	1.03424000

TS4-Ni

Thermal correction to Gibbs Free Energy= 0.502627 Hartree

SCF Done: E(RM06) = -2134.92022901 Hartree

0 1

C	0.18970000	-0.06054600	2.59497200
C	1.11143300	0.05860000	3.62917800
C	1.78296200	-1.08410300	4.05964400
C	1.50931400	-2.30507800	3.44110800
C	0.57586700	-2.34683000	2.40833800
H	1.26024300	1.03881700	4.06708400
H	2.50607600	-1.02727000	4.86781000
H	2.00875800	-3.21767000	3.74689300
H	0.31547700	-3.26119600	1.89029600
N	-0.06417500	-1.24067700	1.99926400
C	-0.61956200	1.09825400	2.09021000
O	-0.42572600	2.23567400	2.55483600
N	-1.50348700	0.71989700	1.14751900
C	-0.45390600	-3.27682100	-0.92347800
O	0.19569700	-2.51736400	-1.69218200
O	-1.12288500	-2.93129500	0.10669700
C	-0.46538000	-4.75648600	-1.26314400
H	-0.67751800	-5.35855600	-0.37828200
H	0.48416700	-5.05079200	-1.71351300
H	-1.25721600	-4.93644100	-1.99838400
C	-2.46325800	1.73473800	0.71403100
C	-2.18572200	2.42181900	-0.65706000
H	-2.41814300	2.52711100	1.47622600

C	-0.68464800	2.75537800	-0.88678000
H	-2.52694000	1.75752700	-1.45411900
H	-0.63850600	3.77543000	-1.29395600
H	-0.16912800	2.82377500	0.07340400
C	-3.89082900	1.17541100	0.79065100
O	-4.77160900	1.35626900	-0.02696900
O	-4.08925200	0.49770100	1.93876200
C	-3.00616400	3.72441100	-0.74940800
H	-2.66287300	4.45137700	-0.00259800
H	-4.06979200	3.53971900	-0.59463300
H	-2.88248800	4.18151100	-1.73723100
C	-5.39244500	-0.08571900	2.08655200
H	-5.39266700	-0.55990800	3.06808700
H	-5.56304900	-0.82805100	1.30287800
H	-6.16997200	0.68025600	2.02926700
C	-2.84702300	-0.79114400	-1.80078000
O	-1.82814800	-0.48731000	-2.47324500
O	-2.87238800	-1.02063100	-0.54216900
C	-4.18958200	-0.86960100	-2.49703500
H	-4.07525400	-0.72755100	-3.57171100
H	-4.83763600	-0.09434000	-2.07730600
H	-4.65572500	-1.83746500	-2.29198100
Pd	0.19805800	-0.36749000	-1.62354500
C	0.08590100	1.89898200	-1.89029700
H	-0.56129000	1.47992800	-2.66988100
H	0.85816600	2.45548600	-2.41303300
C	2.05384900	-0.09116200	-0.93507800
C	1.89556100	1.18235300	-0.90124400
C	3.08428300	-1.05754000	-0.55275000
C	3.24088400	-2.27450200	-1.23772000
C	3.96541900	-0.75038100	0.50241200
C	4.26374100	-3.15351600	-0.88245500
H	2.55021100	-2.52369500	-2.03403800
C	4.98049200	-1.63661700	0.85509200
H	3.84752700	0.18653400	1.03747000
C	5.13507100	-2.84060200	0.16281200
H	4.37950000	-4.08742900	-1.42531000
H	5.65464800	-1.38501500	1.66917400
H	5.92910900	-3.52939000	0.43702500
C	2.53557600	2.45343300	-0.59924100
C	2.37792900	3.07373900	0.65457900
C	3.37060700	3.05292900	-1.56276600
C	3.05445000	4.26197900	0.93328600
H	1.71802100	2.64053200	1.39961400
C	4.04421700	4.23655700	-1.27264200
H	3.49359600	2.57347100	-2.52933700
C	3.88661500	4.84440100	-0.02392300
H	2.92305900	4.73447900	1.90219300
H	4.69304000	4.68408700	-2.01984900
H	4.40919000	5.77004200	0.19950500
Ni	-1.39168800	-1.10513200	0.60877100

6-Ni

Thermal correction to Gibbs Free Energy= 0.503885 Hartree

SCF Done: E(RM06) = -2134.96177579 Hartree

0 1

C	0.04451800	0.07516900	2.68138700
C	0.91759400	0.14184700	3.76158000
C	1.44945600	-1.04391200	4.26362200
C	1.08824900	-2.25313100	3.66751700
C	0.21240000	-2.24040000	2.58513700
H	1.13795200	1.11894400	4.17564000
H	2.13077300	-1.02896900	5.10901700
H	1.47544100	-3.19925600	4.02899500
H	-0.11098300	-3.14631400	2.08971600
N	-0.29125100	-1.09310600	2.10331500
C	-0.61883100	1.28978300	2.10576100
O	-0.34711800	2.41370900	2.55825000
N	-1.47839500	0.97655400	1.11302500
C	-0.68864400	-3.29890500	-0.62294700
O	0.12967900	-2.71539100	-1.39454000
O	-1.45597500	-2.75607600	0.23347400
C	-0.76419100	-4.80876500	-0.75006900
H	-1.17725600	-5.25610700	0.15506400
H	0.22282600	-5.21870100	-0.97144300
H	-1.42439700	-5.05086800	-1.58980100
C	-2.27595700	2.09133700	0.59192500
C	-1.77246200	2.72692400	-0.73943800
H	-2.21117600	2.87939500	1.35666700
C	-0.22266600	2.89076700	-0.75528100
H	-2.08007200	2.07023400	-1.56224700
H	0.01435100	3.90612200	-1.08935800
H	0.15561400	2.82297200	0.26373800
C	-3.75937000	1.70958900	0.52920800
O	-4.51772800	1.94672400	-0.39029100
O	-4.15833300	1.13631200	1.68132700
C	-2.44274500	4.09661100	-0.95752000
H	-2.15113400	4.79876700	-0.16676900

H	-3.52991800	4.01298100	-0.96832600
H	-2.12450400	4.52585200	-1.91372700
C	-5.52878100	0.71174500	1.71249900
H	-5.69619900	0.33064500	2.71996500
H	-5.69255900	-0.07695000	0.97396000
H	-6.19996500	1.54774800	1.50062600
C	-3.03896600	-0.79683300	-1.77150300
O	-2.03212400	-0.90375500	-2.51560900
O	-3.01633900	-0.67389400	-0.49534500
C	-4.41996500	-0.78402700	-2.39124300
H	-4.35627000	-0.88685100	-3.47450400
H	-4.91004900	0.15649300	-2.12307200
H	-5.01696200	-1.59977200	-1.97247400
Pd	-0.00135700	-0.65056500	-1.70415300
C	0.55453400	1.91384700	-1.68072000
H	5.54909700	-3.54601400	0.67645700
C	2.96570400	2.10330400	-0.75466600
C	3.00105700	2.84825400	0.43671000
C	4.04355100	2.21155500	-1.64778100
C	4.08596200	3.68007200	0.71933300
H	2.18508500	2.77070800	1.14951900
C	5.12777700	3.04172000	-1.36186000
H	4.02749800	1.63562000	-2.56882800
C	5.15084100	3.78064900	-0.17775600
H	4.09806800	4.24831600	1.64527400
H	5.95395600	3.11026600	-2.06411300
H	5.99390600	4.42834900	0.04552400
Ni	-1.54934400	-0.87667500	0.65295300
H	-0.20607700	1.19015500	-2.09906300
H	0.85985200	2.42469100	-2.60323700
C	1.79036400	-0.11341600	-1.03923800
C	1.80105900	1.22860100	-1.09834000
C	2.80867800	-1.05571800	-0.56130500
C	3.18550700	-2.16222100	-1.34596500
C	3.43041500	-0.88011700	0.69006200
C	4.16697900	-3.04698000	-0.90504300
H	2.69854500	-2.32036600	-2.30235000
C	4.40999400	-1.76969400	1.12916500
H	3.14724300	-0.03538100	1.30859100
C	4.78399400	-2.85498200	0.33385900
H	4.45098500	-3.88927200	-1.52978200
H	4.88506200	-1.61177000	2.09366200

8. Cartesian coordinates of structures in Figure S1

$[Pd(OAc)_2]_3$

Thermal correction to Gibbs Free Energy= 0.241572 Hartree

SCF Done: E(RM06) = -1754.444965 Hartree

0 1

Pd	-1.05859700	-1.51250700	0.00121100
C	1.15783100	-2.36264100	-1.87212500
O	0.07520200	-2.65066600	-1.28123300
O	1.92088000	-1.37587700	-1.64106200
C	1.54409000	2.12450600	1.87720400
O	0.52524100	2.59400700	1.28898000
O	2.13034500	1.02450000	1.64119100
C	2.15115200	2.98825600	2.96347000
H	2.69252900	2.37019600	3.68059900
H	2.86102800	3.68098600	2.49889500
H	1.37556100	3.57173300	3.46106200
C	1.61032700	-3.31960500	-2.95554300
H	0.74812900	-3.77550800	-3.44360300
H	2.23766300	-2.80019600	-3.68092900
H	2.20536400	-4.11280300	-2.49034300
Pd	-0.78244900	1.67371100	-0.00202600
C	1.47059900	2.18187500	-1.87385300
O	2.26202900	1.38713600	-1.28539800
O	0.23502300	2.34959400	-1.64063600
C	1.06621300	-2.39930600	1.87871700
O	-0.17990500	-2.34961300	1.64630800
O	1.98380500	-1.75757600	1.28660200
C	1.50809800	-3.35611900	2.96664900
H	2.40530900	-2.98056700	3.46016400
H	0.70346100	-3.50833200	3.68692200
H	1.74500100	-4.32034200	2.50433000
C	2.07112500	3.05335400	-2.95747400
H	2.90409500	2.54045300	-3.43956500
H	1.30857600	3.32719000	-3.68766400
H	2.44917300	3.97087200	-2.49392500
Pd	1.84257500	-0.16064000	0.00016000
C	-2.62063500	0.27906300	1.87036200
O	-2.51769500	-0.83801100	1.28223500
O	-1.95827300	1.33519600	1.63563100
C	-3.67493400	0.37483700	2.95383400
H	-3.78912300	-0.58628300	3.45671000
H	-3.41535400	1.15789200	3.66710300
H	-4.63012400	0.63581900	2.48576600
C	-2.61954000	0.17535900	-1.87971600
O	-2.14909400	-0.97863200	-1.64186800
O	-2.32692700	1.25943200	-1.29384200
C	-3.67246300	0.25785600	-2.96551300
H	-4.65738000	0.13366500	-2.50274700
H	-3.63972300	1.23285900	-3.45298100
H	-3.53078400	-0.54412000	-3.69101600

1

Thermal correction to Gibbs Free Energy= 0.251892 Hartree

SCF Done: E(RM06) = -841.1069043 Hartree

0 1

C	2.59887200	-0.20253200	-0.24316200
C	3.66984800	-0.93916200	-0.75255300
C	4.96026500	-0.58115600	-0.36830800
C	5.12727400	0.49285800	0.50517200
C	3.99107400	1.16689900	0.95928100
H	3.46819600	-1.76195000	-1.42783000
H	5.81952300	-1.12922100	-0.74268400
H	6.11344300	0.80640700	0.83122700
H	4.08480000	2.00936000	1.64102500
N	2.74813000	0.83322400	0.59809900
C	1.19255800	-0.57577600	-0.65031400
O	0.97031400	-1.51187400	-1.42242400
N	0.22838700	0.19582500	-0.09824400
H	0.51112400	0.97047600	0.49191100
C	-1.17734100	0.02091900	-0.41011700
C	-1.81972800	-1.14275700	0.41756400
H	-1.26726400	-0.24039200	-1.46948900
C	-3.19614000	-1.55976600	-0.13522400
C	-3.70233500	-2.89633600	0.41975400
H	-3.93279400	-0.77404600	0.06909100
H	-3.12814500	-1.63468100	-1.22805600
H	-4.64899900	-3.17763500	-0.05262500
H	-2.98382200	-3.70146900	0.22890000
H	-3.87616300	-2.85402800	1.49953400
C	-1.85712000	1.36464300	-0.17375400
O	-1.41471500	2.23695000	0.54786200
O	-3.01592800	1.46433400	-0.84411200
C	-3.74966500	2.69083100	-0.64551700
H	-4.64846200	2.59367200	-1.25239800
H	-3.15437000	3.54562800	-0.97285200
H	-4.00752000	2.81552900	0.40820200

H	-1.13214600	-1.98079300	0.24741900
C	-1.84229500	-0.83008200	1.91960100
H	-2.12756100	-1.71269300	2.49818200
H	-2.55758600	-0.03329400	2.15505000
H	-0.85685400	-0.51097600	2.27127000

TS1

Thermal correction to Gibbs Free Energy= 0.43127 Hartree

SCF Done: E(RM06) = -2010.722943 Hartree

0 1

Pd	-2.12877900	-1.15915600	0.53161200
O	-1.36455600	0.13512200	-2.33041800
C	-2.32274200	-0.70478200	-2.37335300
O	-2.74600800	-1.43369000	-1.43121000
C	-3.00804800	-0.86043000	-3.71834900
H	-4.01065200	-1.26897400	-3.58975700
H	-3.04560900	0.09866600	-4.23785700
H	-2.42021500	-1.55283400	-4.33029100
Pd	0.06361100	0.23981200	-0.74026300
C	-4.62512100	0.03941800	1.09827400
O	-3.42548000	0.38275300	0.68132400
O	-4.96823900	-1.09876700	1.39915800
C	-5.58075100	1.22546900	1.17069800
H	-6.54142300	0.90303500	1.57383100
H	-5.16024100	2.01289300	1.80320600
H	-5.72874000	1.64759000	0.17145600
C	-0.49272900	-0.24530900	2.73160000
O	0.27414700	0.25866200	1.87043500
O	-1.56765700	-0.89136500	2.48642200
C	0.22961900	-2.69026000	-0.43879800
O	-0.77029800	-2.72659700	0.32493200
O	0.67543100	-1.66340500	-1.06519900
C	0.98596900	-3.97696200	-0.68248900
H	0.56833100	-4.46376300	-1.57053300
H	2.04081600	-3.76707500	-0.86583600
H	0.86264800	-4.64955500	0.16715300
C	-0.12827800	-0.12277100	4.20166600
H	0.28292600	0.86908500	4.40261200
H	-0.99133900	-0.31363100	4.83986400
H	0.64830000	-0.86025600	4.43152400
C	0.72606600	3.02348900	-0.24935800
C	0.56220300	4.39211400	-0.06421500
C	-0.73041500	4.91222700	-0.00667100
C	-1.81406800	4.04770200	-0.14525300
C	-1.58520100	2.68852100	-0.35507700
H	1.44273000	5.01678200	0.02608100
H	-0.88699100	5.97498400	0.14610500
H	-2.83480500	4.40887500	-0.09361300
H	-2.39138900	1.96831300	-0.44063000
N	-0.33497800	2.20212600	-0.40922500
C	2.10559500	2.42719300	-0.34601700
O	3.01973500	3.06153300	-0.84330200
C	3.54840100	0.46363600	-0.01028700
C	4.07475800	-0.08256200	1.33943700
H	4.22508500	1.26211000	-0.33722400
C	5.60226900	-0.30955700	1.26739400
H	3.90981200	0.74009800	2.05167000
C	6.25763500	-0.56472500	2.62941100
H	5.80679000	-1.14675700	0.59267100
H	6.06752000	0.57744000	0.81643600
H	5.90892000	-1.49619300	3.08532300
C	3.55086700	-0.54410500	-1.17141500
O	4.06625600	-1.64251000	-1.14936300
O	2.97716300	-0.00813100	-2.26025700
C	3.31598000	-1.31699600	1.85536800
H	3.51400800	-2.18468700	1.22236900
H	2.23727000	-1.14407500	1.88572200
H	3.63336100	-1.55674800	2.87405900
C	2.91553900	-0.85702500	-3.42472300
H	2.49758100	-0.23526400	-4.21518600
H	2.26467500	-1.70854600	-3.22044000
H	3.91434100	-1.20661600	-3.69356900
H	7.34453800	-0.64045400	2.52380300
H	6.04873000	0.25058800	3.33216900
N	2.21904800	1.11069500	0.11589800
H	1.71303900	0.91050900	0.99284600

2

Thermal correction to Gibbs Free Energy= 0.375068 Hartree

SCF Done: E(RM06) = -1781.694835 Hartree

0 1

C	0.42635300	3.04102700	0.34518200
C	0.21001300	4.34731100	0.76895700
C	-0.92562700	5.02053500	0.32269500
C	-1.80937000	4.36777700	-0.53888600
C	-1.53332900	3.06011100	-0.92842000
H	0.94208600	4.79365400	1.43165200
H	-1.11974300	6.04062100	0.63863400

H	-2.70250300	4.85735000	-0.91034800
H	-2.17165400	2.49925000	-1.60012300
N	-0.43762700	2.42348700	-0.48511400
C	1.63569600	2.25219100	0.76631400
O	2.49807000	2.78176500	1.48037100
N	1.63917300	0.98745600	0.28374000
Pd	0.09268400	0.51381300	-0.93865000
C	-2.37524100	-0.54228100	-2.43088100
O	-2.70906900	-1.37549700	-1.52974100
O	-1.45789800	0.32710400	-2.36119200
C	-3.15169400	-0.62493900	-3.72938600
H	-4.13593400	-1.06391800	-3.56427000
H	-2.59633800	-1.26329700	-4.42486800
H	-3.24034700	0.36567200	-4.17859600
C	2.82085400	0.17200400	0.56482200
C	2.64927100	-0.90222500	1.67894900
H	3.57932300	0.88395800	0.92246500
C	1.77755900	-0.37279800	2.83591200
H	2.14311500	-1.77260400	1.24174100
C	1.53060800	-1.39252000	3.95359400
H	2.24597400	0.52973600	3.25051400
H	0.80782000	-0.06577300	2.43446800
H	2.44907700	-1.66453600	4.48311900
C	3.40166000	-0.41093900	-0.73074100
O	3.86270100	-1.52797600	-0.86353600
O	3.41246700	0.50447800	-1.71887800
C	4.03380200	-1.34995300	2.18271000
H	4.53796400	-0.52484300	2.70185600
H	4.66839700	-1.68298100	1.36054800
H	3.94410700	-2.17975100	2.88916300
C	3.94771500	0.05273600	-2.97482800
H	3.88678800	0.90976500	-3.64491600
H	3.35357200	-0.77860500	-3.36096800
H	4.98510600	-0.27005800	-2.85846100
H	0.83458700	-0.98611000	4.69490200
C	0.44991300	-2.50269700	-0.97722000
O	-0.54565700	-2.75135500	-0.22498500
O	0.81431300	-1.37112600	-1.41700800
C	1.28960300	-3.68802300	-1.40103600
H	1.14257200	-3.85782900	-2.47255200
H	1.00263600	-4.58375200	-0.85083600
H	2.34301700	-3.44774200	-1.23980100
Pd	-1.95005800	-1.39413900	0.37007900
C	-2.67380400	-0.47779400	2.53021600
O	-3.28132500	-0.18112200	1.44167200
C	-3.13852600	0.04299500	3.85330000
H	-2.28081400	0.23209900	4.50191900
H	-3.76726500	-0.71503400	4.33272100
H	-3.72743800	0.95129700	3.71667000
O	-1.66517000	-1.26031300	2.43897700
H	1.08408300	-2.31091500	3.55633300

TS2-δ

Thermal correction to Gibbs Free Energy= 0.374968 Hartree

SCF Done: E(RM06) = -1781.661777 Hartree

0 1

C	-1.03580000	2.69805900	-0.32086800
C	-1.87926600	3.79723200	-0.20419100
C	-3.09145500	3.79141800	-0.89160200
C	-3.42454500	2.68530100	-1.67735400
C	-2.53588100	1.61596000	-1.75104200
H	-1.55612700	4.62109400	0.42118600
H	-3.76808500	4.63715800	-0.81986400
H	-4.35624400	2.64435100	-2.23040100
H	-2.72749000	0.72723600	-2.34110700
N	-1.37289300	1.63732000	-1.08075700
C	0.29519100	2.63195300	0.37749100
O	0.66615700	3.57469800	1.08999700
N	0.96582200	1.48231800	0.12947800
Pd	0.03007200	0.16403700	-1.09112300
C	-1.56114300	-2.24887300	-1.91271400
O	-1.48688100	-2.75206900	-0.75282400
O	-1.13330800	-1.11015400	-2.29252400
C	-2.20688900	-3.10960200	-2.98269300
H	-2.72122600	-2.48452900	-3.71482100
H	-2.89582000	-3.82675800	-2.53541200
H	-1.41901400	-3.66246000	-3.50557700
C	2.28865900	1.33924300	0.72964200
C	2.40198700	0.36800500	1.94572200
H	2.54500200	2.34410000	1.09561900
C	1.16687900	0.38234200	2.88693200
H	2.53333300	-0.64571900	1.56044800
C	0.22993200	-0.85010100	2.82150900
H	1.54038800	0.40688200	3.91916900
H	0.59991000	1.30959400	2.76263900
H	0.82786000	-1.75867400	2.73683800
C	3.33938200	1.02113200	-0.34341200
O	4.24613000	0.21851000	-0.22915100

O	3.18078600	1.79466800	-1.43242600
C	3.66653400	0.72250400	2.75367400
H	3.56360300	1.71029900	3.21940800
H	4.55772800	0.72576900	2.12362000
H	3.82282100	-0.00770700	3.55483800
C	4.13695100	1.58479400	-2.48656200
H	3.86965800	2.29333000	-3.26984400
H	4.07257300	0.55988500	-2.85895900
H	5.15191900	1.77363000	-2.12855100
H	-0.34614700	-0.95454100	3.74859400
C	1.83525000	-2.16510300	-0.46340400
O	1.12693600	-2.53572800	0.52459100
O	1.60291200	-1.18296400	-1.23139000
C	3.09991100	-2.94715800	-0.73886800
H	3.17673100	-3.81156100	-0.07989800
H	3.95012700	-2.27590300	-0.58690900
H	3.10870000	-3.26810900	-1.78388600
Pd	-0.74435300	-1.75908900	0.95417500
C	-2.84884800	-0.09627400	1.90968900
O	-2.69818800	-1.24098100	1.37184500
C	-4.25375000	0.43013200	2.07463900
H	-4.40412500	1.25207900	1.36746000
H	-4.37808700	0.83365200	3.08190200
H	-4.98823100	-0.35116100	1.88199000
O	-1.87471800	0.61607400	2.29450800
H	-0.83052700	-0.21923400	2.23956300

3-δ

Thermal correction to Gibbs Free Energy= 0.496511 Hartree

SCF Done: E(RM06) = -2091.83529 Hartree

0 1

C	-2.67650100	2.29680100	-0.41404700
C	-3.12365300	3.58224600	-0.70010100
C	-2.83490000	4.61059000	0.19603400
C	-2.10468000	4.32285100	1.35057200
C	-1.68541000	3.01457700	1.57751900
H	-3.68958000	3.73172800	-1.61222800
H	-3.17691500	5.62213000	0.00056600
H	-1.86068800	5.09416700	2.07233400
H	-1.12861800	2.71553100	2.45760300
N	-1.96902600	2.03589600	0.70432400
C	-2.95526900	1.13161800	-1.32777800
O	-3.55621000	1.31995400	-2.39497300
N	-2.47201400	-0.03838400	-0.85057800
Pd	-1.57404600	0.05099000	0.97135000
C	0.54261400	0.19255900	3.04684000
O	1.40770100	-0.11986400	2.19965500
O	-0.71291000	0.37973300	2.83436200
C	0.97617300	0.35454800	4.49600700
H	2.01848700	0.67370500	4.54547800
H	0.89222300	-0.61730500	4.99475500
H	0.33219900	1.06065400	5.02329400
C	-2.66981000	-1.23077800	-1.66592900
C	-1.36582200	-1.87607500	-2.22845800
H	-3.28863000	-0.90728100	-2.51507100
C	-0.26127100	-0.84757300	-2.58820500
H	-0.97062400	-2.53365800	-1.45350400
H	-0.14347300	-0.81958500	-3.68304500
H	-0.56572300	0.16674100	-2.31477100
C	-3.51612000	-2.26826200	-0.91288900
O	-3.31585200	-3.46793500	-0.90071700
O	-4.56414800	-1.69513800	-0.29457300
C	-1.69582400	-2.75395400	-3.44897400
H	-2.09768800	-2.14628000	-4.26929900
H	-2.42466600	-3.52749300	-3.19913300
H	-0.79044400	-3.24969600	-3.81649300
C	-5.42006000	-2.59262500	0.43353500
H	-6.21303000	-1.97058400	0.84753700
H	-4.86112800	-3.08271200	1.23412100
H	-5.83659800	-3.35329000	-0.23131000
C	-0.63899100	-2.81306800	1.08604200
O	0.44692100	-2.57155100	0.49679300
O	-1.56628600	-1.97406800	1.33758100
C	-0.93088700	-4.23970700	1.50644400
H	-0.04808900	-4.86760300	1.38598200
H	-1.74386500	-4.61554400	0.87762600
H	-1.27264700	-4.26083900	2.54428300
Pd	1.25302300	-0.62226500	-0.03093600
C	1.11979000	-1.15048400	-2.00865100
H	1.34921700	-2.22075300	-1.99635900
H	1.90524700	-0.60863600	-2.53844900
C	3.27520500	0.04360700	-0.33879900
C	2.54399600	1.05566300	-0.49239700
C	4.43464300	-0.80544300	-0.32084500
C	4.36544500	-2.15754400	0.06385300
C	5.68013100	-0.26084600	-0.70046400
C	5.51620900	-2.94284500	0.06719800
H	3.40871200	-2.57745900	0.35667100

C	6.82346500	-1.05414600	-0.68976900
H	5.73751000	0.77964000	-1.00435900
C	6.74621800	-2.39663900	-0.30715100
H	5.45121900	-3.98513600	0.36469400
H	7.77658700	-0.62480200	-0.98422300
H	7.64031800	-3.01287800	-0.30221900
C	2.26709600	2.45195000	-0.70992000
C	3.28279200	3.39843000	-0.45717800
C	1.02712900	2.89935200	-1.19869400
C	3.05795700	4.75130300	-0.69501600
H	4.23920200	3.06094300	-0.07056400
C	0.81302100	4.25481200	-1.44152100
H	0.24389300	2.17594000	-1.39328400
C	1.82444900	5.18481400	-1.19031400
H	3.84789600	5.46927900	-0.49479600
H	-0.14683800	4.58315800	-1.82818400
H	1.65435500	6.24053900	-1.37960200

TS3-δ

Thermal correction to Gibbs Free Energy= 0.497773 Hartree

SCF Done: E(RM06) = -2091.801292 Hartree

0 1

C	-0.26361900	0.60685300	2.88928200
C	0.41742300	1.01999300	4.02951700
C	1.30052800	0.13410400	4.64508800
C	1.47702100	-1.13918700	4.10051200
C	0.76558800	-1.49000600	2.95582700
H	0.22858900	2.02126700	4.39871100
H	1.84247600	0.42948000	5.53806000
H	2.15323800	-1.85783900	4.54962100
H	0.84757100	-2.46211500	2.48409500
N	-0.07922000	-0.62533000	2.37327700
C	-1.22723000	1.50866300	2.16282200
O	-1.39886500	2.67055200	2.56129600
N	-1.79858900	0.91462000	1.09135700
Pd	-1.24696200	-1.01065300	0.74464200
C	0.16075200	-3.37505900	-0.43843400
O	0.59910000	-2.68682900	-1.39984900
O	-0.52562900	-2.96698900	0.55781300
C	0.50752200	-4.85447700	-0.45025300
H	1.58816300	-4.96474100	-0.31350400
H	0.25501100	-5.28071800	-1.42444500
H	-0.01770500	-5.39106100	0.33996900
C	-2.71168800	1.71674200	0.28532000
C	-2.17770400	2.09764600	-1.13066600
H	-2.86869800	2.64588700	0.85189300
C	-0.66265700	2.45216500	-1.14086100
H	-2.33262100	1.23305400	-1.77727700
H	-0.55351600	3.51607100	-1.38728400
H	-0.24644100	2.35381600	-0.13686500
C	-4.08961300	1.04388900	0.20465100
O	-4.78067500	0.95958300	-0.79250600
O	-4.48638000	0.59373500	1.40881100
C	-2.99242300	3.26792800	-1.71238400
H	-2.87031000	4.16924400	-1.09881500
H	-4.05485400	3.02673600	-1.77017000
H	-2.64323000	3.50673700	-2.72308800
C	-5.77163300	-0.05072300	1.43507600
H	-5.93475100	-0.33738900	2.47352600
H	-5.76584300	-0.93290600	0.79080000
H	-6.55403700	0.63419800	1.09881600
C	-2.54949700	-1.32673600	-1.94534500
O	-1.52587700	-0.93400800	-2.56217700
O	-2.68191900	-1.42166500	-0.67637800
C	-3.78670400	-1.69146400	-2.74254000
H	-3.56787200	-1.70753600	-3.81037500
H	-4.55868500	-0.94575100	-2.52959100
H	-4.16523300	-2.66491900	-2.42005100
Pd	0.45324100	-0.55174800	-1.66713900
C	0.18144900	1.66181500	-2.14594900
H	-0.41445600	1.14316200	-2.90567400
H	0.89517400	2.26729500	-2.69492900
C	2.27944200	-0.08649500	-1.00408000
C	2.04772800	1.16953000	-1.12321700
C	3.35050800	-0.95826400	-0.52056900
C	3.62497100	-2.19146800	-1.13688500
C	4.14685300	-0.54648000	0.56548400
C	4.67930300	-2.98426600	-0.68418200
H	3.00104200	-2.52193800	-1.95825500
C	5.19438400	-1.34739400	1.01665400
H	3.94165300	0.40462200	1.04636800
C	5.46537500	-2.56815400	0.39304200
H	4.88674000	-3.93077200	-1.17520100
H	5.80163600	-1.01583800	1.85419200
H	6.28316800	-3.19041700	0.74481100
C	2.54436800	2.51007200	-0.86458600
C	2.46739500	3.04799100	0.43387000
C	3.14648000	3.26987500	-1.88573600

C	2.98914600	4.31321500	0.70311700
H	1.99657800	2.47146100	1.22340000
C	3.66782200	4.53112600	-1.60870700
H	3.21574500	2.85704500	-2.88771300
C	3.58818200	5.05665200	-0.31539700
H	2.92154400	4.71906700	1.70790900
H	4.13933800	5.10420600	-2.40137500
H	3.99154400	6.04245400	-0.10400300

4-δ

Thermal correction to Gibbs Free Energy= 0.500947 Hartree

SCF Done: E(RM06) = -2091.84787 Hartree

0 1

C	-0.97009500	-0.42500900	3.08389900
C	-0.53971900	-0.58051200	4.39681000
C	0.10496500	-1.76166000	4.75927000
C	0.30132000	-2.75190100	3.79510000
C	-0.15229400	-2.53237500	2.49715400
H	-0.72406800	0.23117800	5.09077400
H	0.44886100	-1.91048700	5.77796600
H	0.79697000	-3.68563500	4.03577700
H	-0.04322000	-3.26486100	1.70706100
N	-0.76839200	-1.38832600	2.16071600
C	-1.64415800	0.83547400	2.61076300
O	-1.76351100	1.79758300	3.38133200
N	-2.02199500	0.77408100	1.31286900
Pd	-1.61914900	-0.96444900	0.34789500
C	-0.16918500	-3.12562100	-1.25030600
O	0.65840700	-2.32578500	-1.78075100
O	-1.11690900	-2.84898800	-0.45023100
C	-0.00808000	-4.58749300	-1.63011400
H	1.05110900	-4.83991900	-1.70810400
H	-0.46364800	-4.74296000	-2.61374600
H	-0.50685400	-5.23432100	-0.90757300
C	-2.49393400	2.00436500	0.68859400
C	-1.50232100	2.59826900	-0.36248300
H	-2.61029800	2.72859600	1.50731900
C	-0.02666200	2.44664800	0.07783200
H	-1.64728500	2.01957800	-1.28142400
H	0.26990800	3.31919900	0.67143600
H	0.07559800	1.57969400	0.72999500
C	-3.89801800	1.81808200	0.09667500
O	-4.27790300	2.25224600	-0.97404700
O	-4.70145000	1.15695400	0.94756100
C	-1.83087300	4.06830900	-0.67371900
H	-1.73909100	4.68459600	0.22896800
H	-2.84229600	4.17442900	-1.06760600
H	-1.13404900	4.46849000	-1.41878700
C	-6.04493100	0.93583700	0.48592600
H	-6.55644900	0.42941400	1.30385800
H	-6.03857300	0.30728400	-0.40761900
H	-6.53543100	1.88447100	0.25472400
C	-2.55267800	-0.39171500	-2.46226800
O	-1.38838100	-0.32918400	-2.92974400
O	-2.86844400	-0.55253900	-1.23165100
C	-3.73262900	-0.23730900	-3.40192500
H	-3.39541600	-0.15289400	-4.43508300
H	-4.28689000	0.66041600	-3.11223400
H	-4.40623400	-1.09214300	-3.29558400
Pd	0.47259900	-0.23618900	-1.74820800
C	0.96386600	2.24363400	-1.09489800
H	0.41358300	1.71477300	-1.92812000
H	1.20528800	3.18292200	-1.60659800
C	2.19985900	0.15976300	-0.85100300
C	2.26132500	1.50191600	-0.71894400
C	3.11305600	-0.90716900	-0.43525400
C	3.70115800	-1.78195700	-1.36888600
C	3.39495400	-1.09498000	0.93290700
C	4.56400300	-2.79266400	-0.94864300
H	3.47856800	-1.65804200	-2.42379600
C	4.25482600	-2.11013100	1.34886300
H	2.94257800	-0.42806300	1.66046500
C	4.84421100	-2.96157100	0.41032000
H	5.01806300	-3.45143200	-1.68348100
H	4.46816000	-2.23446500	2.40696100
H	5.51571400	-3.75115000	0.73512800
C	3.43075700	2.29938800	-0.26791200
C	3.25727800	3.58441900	0.28158800
C	4.75043100	1.82723200	-0.41717800
C	4.34932100	4.35205000	0.68741100
H	2.26044900	3.99224700	0.40530000
C	5.83975000	2.59508300	-0.01335000
H	4.92324200	0.85756300	-0.86838800
C	5.64727500	3.86109600	0.54613400
H	4.18095600	5.33640700	1.11514000
H	6.84539900	2.20607900	-0.14674000
H	6.49826600	4.45880200	0.85939400
